# Use and validity of child neurodevelopment outcome measures in studies on prenatal exposure to psychotropic and analgesic medications – A systematic review

**DOI:** 10.1371/journal.pone.0219778

**Published:** 2019-07-11

**Authors:** Sarah Hjorth, Rebecca Bromley, Eivind Ystrom, Angela Lupattelli, Olav Spigset, Hedvig Nordeng

**Affiliations:** 1 PharmacoEpidemiology and Drug Safety Research Group, Department of Pharmacy, Faculty of Mathematics and Natural Sciences, University of Oslo, Oslo, Norway; 2 Division of Evolution and Genomic Science, School of Biological Sciences, Faculty of Biology, Medicine and Health, University of Manchester, Manchester Academic Health Science Centre, Manchester, England; 3 Royal Manchester Children's Hospital, Central Manchester University Hospitals NHS Foundation Trust, Manchester, England; 4 Department of Mental Disorders, Norwegian Institute of Public Health, Oslo, Norway; 5 PROMENTA Research Center, Department of Psychology, University of Oslo, Oslo, Norway; 6 Department of Clinical Pharmacology, St. Olav's University Hospital, Trondheim, Norway; 7 Department of Clinical and Molecular Medicine, Norwegian University of Science and Technology, Trondheim, Norway; 8 Department of Child Health and Development, Norwegian Institute of Public Health, Oslo, Norway; IRCCS E. Medea, ITALY

## Abstract

In recent years there has been increased attention to child neurodevelopment in studies on medication safety in pregnancy. Neurodevelopment is a multifactorial outcome that can be assessed by various assessors, using different measures. This has given rise to a debate on the validity of various measures of neurodevelopment. The aim of this review was twofold. Firstly we aimed to give an overview of studies on child neurodevelopment after prenatal exposure to central nervous system acting medications using psychotropics and analgesics as examples, giving special focus on the use and validity of outcome measures. Secondly, we aimed to give guidance on how to conduct and interpret medication safety studies with neurodevelopment outcomes. We conducted a systematic review in the MEDLINE, Embase, PsycINFO, Web of Science, Scopus, and Cochrane databases from inception to April 2019, including controlled studies on prenatal exposure to psychotropics or analgesics and child neurodevelopment, measured with standardised psychometric instruments or by diagnosis of neurodevelopmental disorder. The review management tool Covidence was used for data-extraction. Outcomes were grouped as motor skills, cognition, behaviour, emotionality, or “other”. We identified 110 eligible papers (psychotropics, 82 papers, analgesics, 29 papers). A variety of neurodevelopmental outcome measures were used, including 27 different psychometric instruments administered by health care professionals, 15 different instruments completed by parents, and 13 different diagnostic categories. In 23 papers, no comments were made on the validity of the outcome measure. In conclusion, establishing neurodevelopmental safety includes assessing a wide variety of outcomes important for the child’s daily functioning including motor skills, cognition, behaviour, and emotionality, with valid and reliable measures from infancy through to adolescence. Consensus is needed in the scientific community on how neurodevelopment should be assessed in medication safety in pregnancy studies.

Review registration number: CRD42018086101 in the PROSPERO database.

## Introduction

Traditionally, studies on medication safety in pregnancy have mainly focused on immediate pregnancy outcomes such as the risk of malformations, low birth weight, and prematurity. In recent years, there has been an increased call for studies on the longer-term safety of prenatal exposure to medications, including studies on child neurodevelopment [1,2].

Psychotropic and analgesic medications share the biological plausibility to affect child neurodevelopment if used prenatally, as they can pass the placenta and bind to targets in the developing brain [3,4]. For these relatively common medications, even small increases in risk of neurodevelopmental delays in offspring could have public health impact [5]. Analgesics have a high frequency of use, with mild analgesics as the most common over the counter medication group in pregnancy with frequencies from 50% to over 70% [6,7]. There has been a marked increase in the use of selective serotonin reuptake inhibitor (SSRI) antidepressants in pregnancy over the last ten years, with the most recent prevalence data ranging from 2–4% in Europe [8,9] to 8% in North America [10]. Other psychotropics are less frequently used in pregnancy, but are important to study given the potential negative impact of a suboptimally treated maternal disorder on the health of mother and child [11,12].

Neurodevelopment comprises a wide range of traits and includes intelligence, language and motor skills, and attentional and executive functioning [13], which all are important for everyday life. When measuring neurodevelopment, some studies use the presence or absence of medical diagnoses, while others use psychometric instruments (questionnaires or tests) completed by parents, teachers, or health care professionals. Recent initiatives have suggested that it is important to consider a spectrum of neurodevelopment, not just diagnostic categories [14]. The question of how to measure neurodevelopmental outcomes in **medication safety studies** was recently raised in an editorial by our research group [2]. There we further call for consensus on how to conduct neurodevelopmental safety studies as part of a future pharmacovigilance framework [2].

Attempts have been made to summarise the literature on specific medication classes as the antidepressants [15], and paracetamol in relation to attention deficit hyperactivity disorder (ADHD, corresponding to the ICD-10 diagnosis hyperkinetic disorder) and autism spectrum disorder (ASD) [16]. A review on antidepressants chose not to present pooled effect estimates given the heterogeneity of the outcome measures [15], whereas a review on paracetamol presented pooled effect estimates for risk of ASD and ADHD, despite clear heterogeneity of outcome measures [16]. In summaries of the evidence, there has been little discussion about the validity and reliability of outcome measures.

Independent of which outcome measures are used, these should be reliable and valid in order for research data to be of value. Evaluations of reliability and validity are important for both medical diagnoses [17] and psychometric instruments [18,19], as both methods to some extent rely on subjective assessments [20]. Reliability is the degree to which the assessment is free from measurement error [18] (see also S1 File for definitions of different types of validity and reliability). Low reliability in an outcome measure mainly introduces random error and can therefore affect the precision of the results. This is particularly problematic in studies with smaller sample sizes. Validity is defined as the extent to which the outcome measure truly measures the construct (e.g. shyness) it is intended to measure [19]. An outcome measure that is not valid in the context where it is used, will give systematically erroneous results, which is problematic regardless of sample size.

To our knowledge, the use and validity of neurodevelopmental outcome measures in studies on medication safety in pregnancy have not yet been systematically evaluated. Hence the primary aim of this review was to provide an overview of the use and validity of child neurodevelopment outcome measures employed in completed medication safety studies on prenatal exposure to psychotropic or analgesic medications. The secondary aim was aid researchers and clinicians in conducting and interpreting studies on maternal prenatal use of psychotropics and analgesics, and child neurodevelopment.

## Methods

A systematic review was conducted in the MEDLINE, Embase, PsycInfo, Scopus, Cochrane, and Web of Science databases from inception to January 17^th^ 2018. The search was updated on April 30^th^ 2019. Reference lists of relevant reviews and included studies were screened to ensure complete coverage of the published literature. The search strategies were developed by the first author and research librarians, with inputs from all authors. Search terms and an example of the search strategy for the MEDLINE database can be found in S2 File. This systematic review was registered in the PROSPERO database (registration number CRD42018086101) and reported according to Preferred Reporting Items for Systematic Reviews (PRISMA).

Studies were considered eligible for inclusion if they fulfilled the criteria for participants, exposures, comparators, outcomes, and study design as described below. Participants were children born to mothers who used psychotropic or analgesic medication in pregnancy. For this review, children were defined as individuals under the age of 18 years. Assessments of neurodevelopment before the child was one month old were not considered, as we wished to exclude transient effects of prenatal exposure to medication. Exposures were maternal antenatal use of analgesic medication (ATC-codes N02 and M01A), antipsychotic medication (ATC-code N05A), anxiolytic medication (ATC-code N05B), hypnotic and sedative medication (ATC-code N05C), or antidepressants (ATC-code N06A) [21]. Antiepileptic medication (ATC-code N03) was not included in the review, as it has been thoroughly investigated in recent reviews [22,23]. Comparators were children born to mothers who did not use the specified medications in pregnancy. Outcomes were all neurodevelopment outcomes that had been assessed either by psychiatric diagnoses, or by standardised psychometric instruments filled in by parents, teachers, or health care professionals. In order to provide an overview, we divided the neurodevelopment outcomes in the following domains:

- Motor skills: Including ICD-10 code F82, specific developmental disorder of motor skills [24]
- Cognition: Including ICD-10 codes F70-79, mental retardation, F80, specific developmental disorder of speech and language, F81, specific developmental disorder of scholastic skills, and F84, pervasive developmental disorders (ASD)
- Behaviour: Including ICD-10 codes F90, hyperkinetic disorders, and F91, conduct disorders
- Emotionality: Including ICD-10 codes F30-39, mood disorders, F40-49, neurotic, stress-related and somatoform disorders (including anxiety), and F93, emotional disorders with onset specific to childhood
- Other: Including sleep disorders (ICD-10 code F51), and tic disorders (ICD-10 code F95)

For all ICD-10 codes, corresponding DSM-5 codes were likewise eligible. Autosomal genetic disorders (Rett syndrome, ICD-10 code F84.2) and unspecific disorders (Other childhood disintegrative disorder, ICD-10 code F84.3) were excluded. As the population for this review was children, we also excluded mental disorders that have their onset in adolescence [25]: bipolar disorder (ICD-10 code F31), schizophrenia (ICD-10 codes F20-29), and substance use disorder (ICD-10 codes F10-19). Randomised controlled trials (RCTs), cohort studies, register-based studies, and case-control studies were considered eligible for inclusion, whereas non-original studies (eg. reviews and editorials), original studies without a comparator group, cross-sectional studies, ecological studies, and animal studies were excluded. No date restrictions were applied, but for resource reasons, the search was limited to peer-reviewed publications in English, French, Italian, Spanish, or one of the Scandinavian languages.

References were imported to the systematic review data management platform Covidence [26]. Title and abstract screening, full text screening, and data extraction were all performed independently by two reviewers (SH and AL). Disagreement was solved by involving a third (HN) and, in case of doubt, a fourth reviewer (RB). When necessary, the authors of the original studies were contacted to provide additional information. Data items extracted from included studies were study design, inclusion and exclusion criteria, study population, duration of follow-up, definition of exposure in pregnancy (including whether gestational age at birth was known, or duration of gestation was estimated), timing, duration and dose of medication, outcome measure used, validity and reliability of outcome measure, covariates and how these were handled, study power, statistical analysis, and effect size. The data extraction forms were developed a priori with inputs from all authors.

Risk of bias in individual studies was assessed using Grading of Recommendations Assessment, Development and Evaluation (GRADE) guidelines [27]. In addition to the items specified in the GRADE guidelines (failure to develop appropriate eligibility criteria, flawed measurement of both exposure and outcome, failure to adequately control confounding, and incomplete follow-up) [27], we assessed how missing data was handled in the studies. The risk of bias assessment was used in determining whether there were other explanations for heterogeneity between study findings than the psychometric properties of the chosen outcome measure. The psychometric properties of the outcome measures in the included studies were assessed on the domains internal consistency, inter-rater and test-retest reliability, construct, content and criterion validity (umbrella term for concurrent and predictive validity) as defined by the COnsensus-based Standards for the selection of health Measurement Instruments (COSMIN) group and following their recommendations [28]. Risk of publication bias for each medication group was assessed according to GRADE guidelines [29]. Due to the expected heterogeneity between the studies in terms of age of the child at measurement and method of assessment, no meta-analysis was planned. However, to ease comparisons of results from different papers, effect sizes (Cohen’s d) [30] were calculated using the metaeff package [31] for Stata [32]. Traditionally, a Cohen’s d with an absolute value of 0.2 is considered a small effect, 0.5 a medium effect and 0.8 or above a large effect [33]. The decision to calculate Cohen’s d was made post hoc as we became aware of how many different effect measures were used in the different studies.

Data was grouped by type of assessment (diagnoses or psychometric instruments), assessor for psychometric instruments (health care professional, parents, or teachers), and by age of the child.

## Results

**Fig 1:**
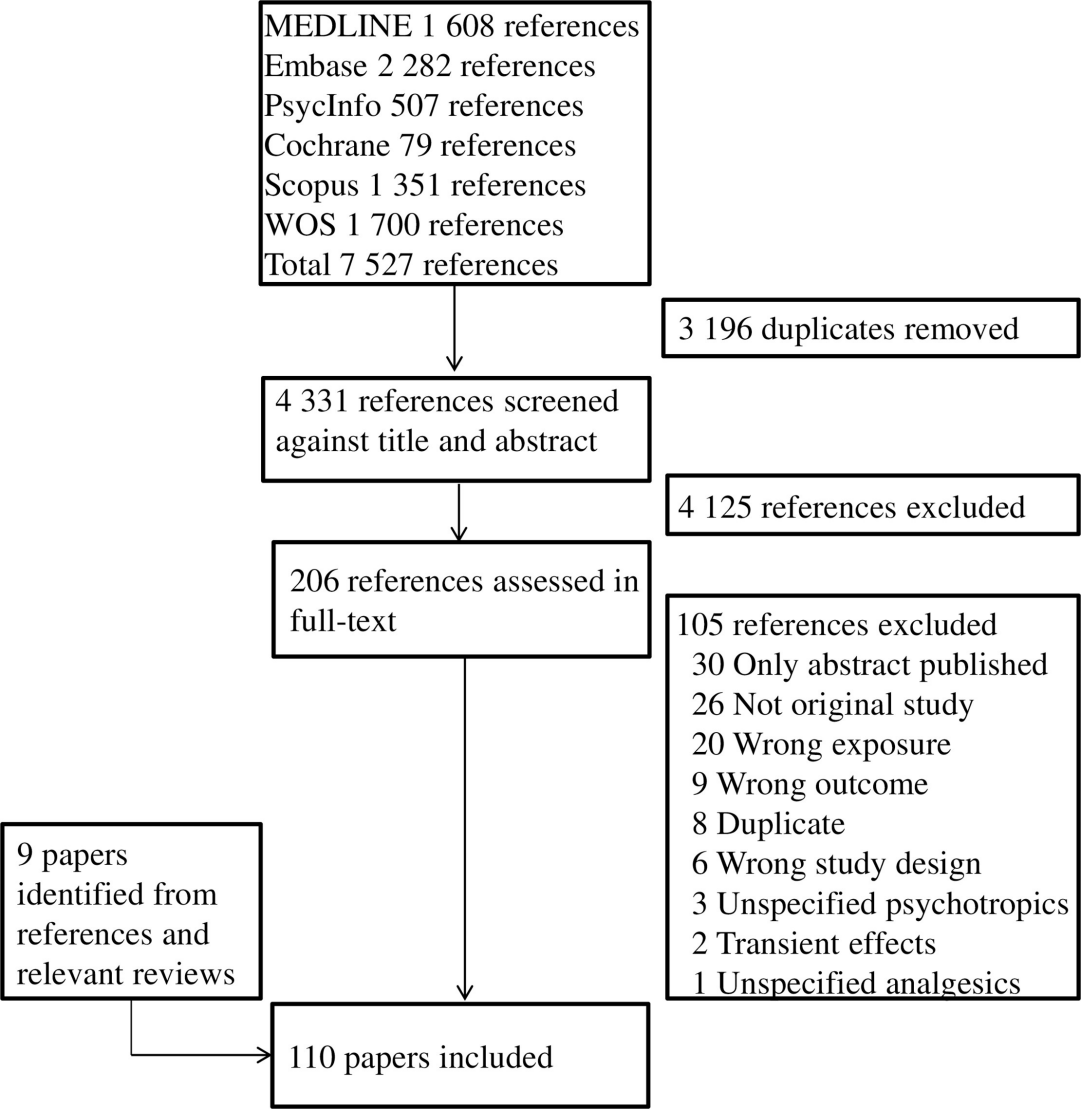
PRISMA flowchart. WOS: Web of Science.

The literature search yielded 7,527 studies. After removal of duplicate records, 4,331 studies were left for title and abstract screening. Of these, 206 were relevant for full text assessment, and 101 were eligible for inclusion. See Fig 1 for PRISMA flowchart. A further 9 studies [34–42] were identified from reference lists of included studies and relevant reviews. Of the eligible papers, 82 focused on psychotropics (S1, S2, S5 and S6 Tables) and 29 on analgesics (S3 and S7 Tables). Some papers studied more than one medication group. Across all 110 papers, 26 papers used information from databases or national health registries. Neurodevelopment was assessed using 27 different psychometric instruments completed by health care professionals, 15 different psychometric instruments completed by parents, and five different psychometric instruments completed by teachers, not counting different versions of the same instrument (S4 Table). In addition, 13 different diagnostic categories were used. The most commonly used psychometric instrument completed by health care professionals was the Bayley Scales of Infant Development, used in 17 papers. The most common psychometric instrument completed by parents was the Childhood Behaviour Checklist, used in 22 papers. The most common diagnostic category was ASD (ICD-10 code F84) [24], used in 18 papers. However, the outcome measures differed for psychotropics and analgesics. For psychotropics, the most common outcome measure was diagnosis of ASD, used in 16 papers, whereas the most common outcome measures for analgesics were the Ages and Stages Questionnaire and the Child Behaviour Checklist, both used in six papers.

In the following, the use of outcome measures will be described by medication group. Only results for the oldest age band are presented here (and in S1–S3 Tables), if a paper had assessed children at multiple time points using the same outcome measure.

### Antidepressants

**Fig 2:**
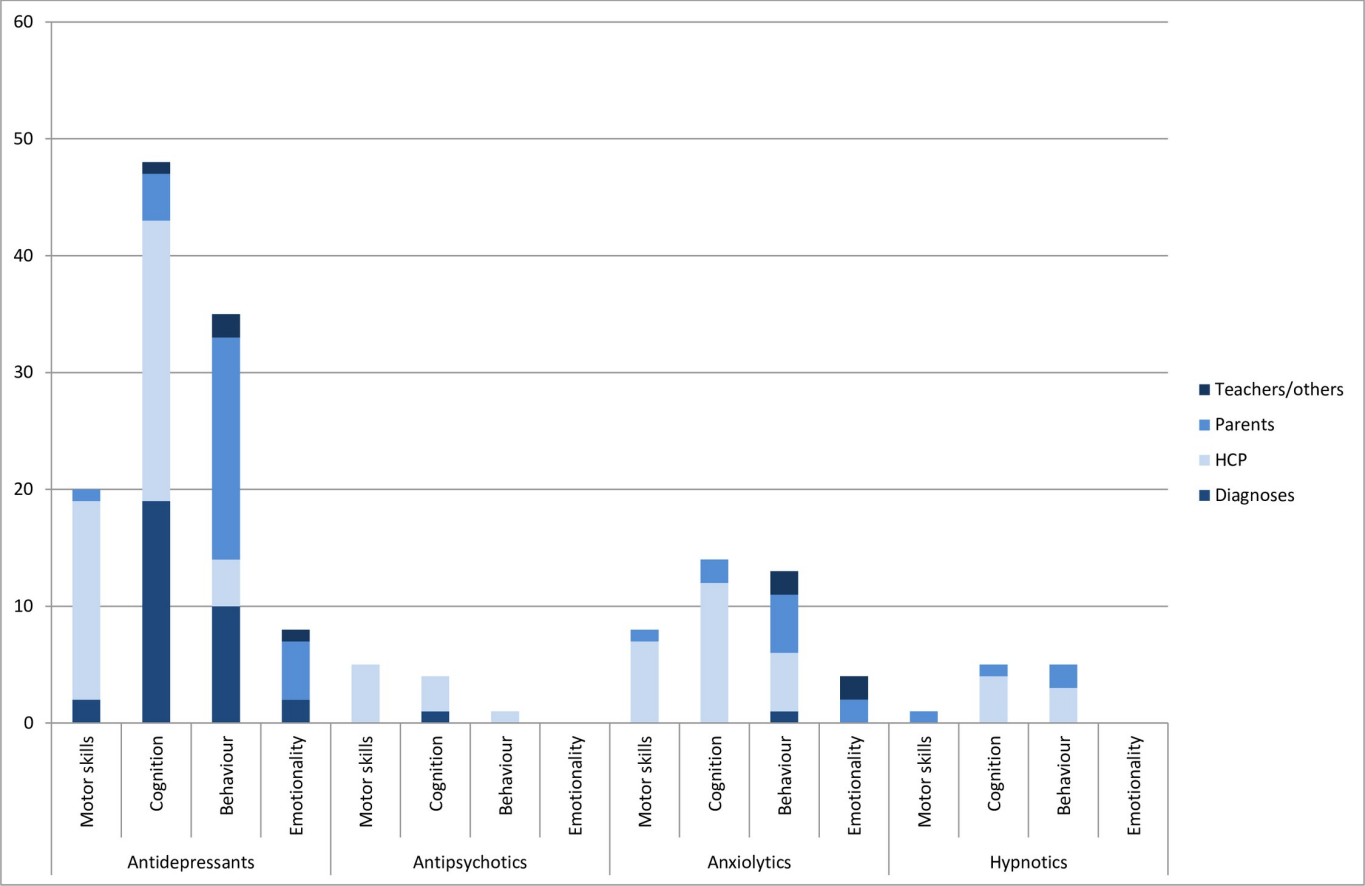
Domains of neurodevelopment evaluated and data sources used in medication safety papers on psychotropics. Some papers had outcomes from more than one domain and assessments from more than one type of assessor. HCP: Health care professionals.

Antidepressants was the most studied medication group with 66 papers [34, 36–38, 41,43–103]. Children had been assessed from the age of one month to 19 years. Diagnostic codes were used in 23 papers, while psychometric instruments were used in the remaining 43 papers (S1 Table). Most work has been done in the cognitive domain, where focus has been divided between intelligence (IQ), language and risk of ASD diagnosis, and most assessments have been done by health care professionals using psychometric instruments (Fig 2). Contrary to other medications, for antidepressants an additional domain of neurodevelopment other than motor skills, cognition, behaviour, and emotionality was assessed, as two papers reported parent assessed sleep problems.

### Anxiolytics

Twenty papers studied neurodevelopment after prenatal exposure to anxiolytics [35,38,40,56,69,79–81,87,92,94,104–112], with ten of the papers published ten or more years ago. Follow-up was available from two months to 15 years (S2 Table). Most papers had used assessment from a health care professional, and only one paper had investigated risk of psychiatric diagnosis [56]. Most work has been done on the cognitive domain; the least has been done on emotionality (Fig 2).

### Antipsychotics

Antipsychotics were investigated in seven papers [69,72,73,81,94,113,114]. Children had been followed from two months to 18 years. All papers had used psychometric instruments completed by health care professionals, except one that used ASD diagnosis [71] (Fig 2). Most papers, five of seven, studied motor skills. Only one paper had investigated a behavioural outcome [113] and none had investigated emotionality.

### Hypnotics and sedatives

Six papers studied hypnotics and sedatives [94,104,109,115–117], and three of these papers looked at overdoses taken for suicide attempts. Follow-up was available from 1 year to 5 years. Three papers did not specify age at follow-up, but other papers from the same study have assessed toddlers. Most papers, four of six, had used psychometric instruments completed by health care professionals (Fig 2). Cognition was studied in four, and behaviour in five papers. One paper investigated motor skills [108], none studied emotionality.

### Paracetamol

**Fig 3:**
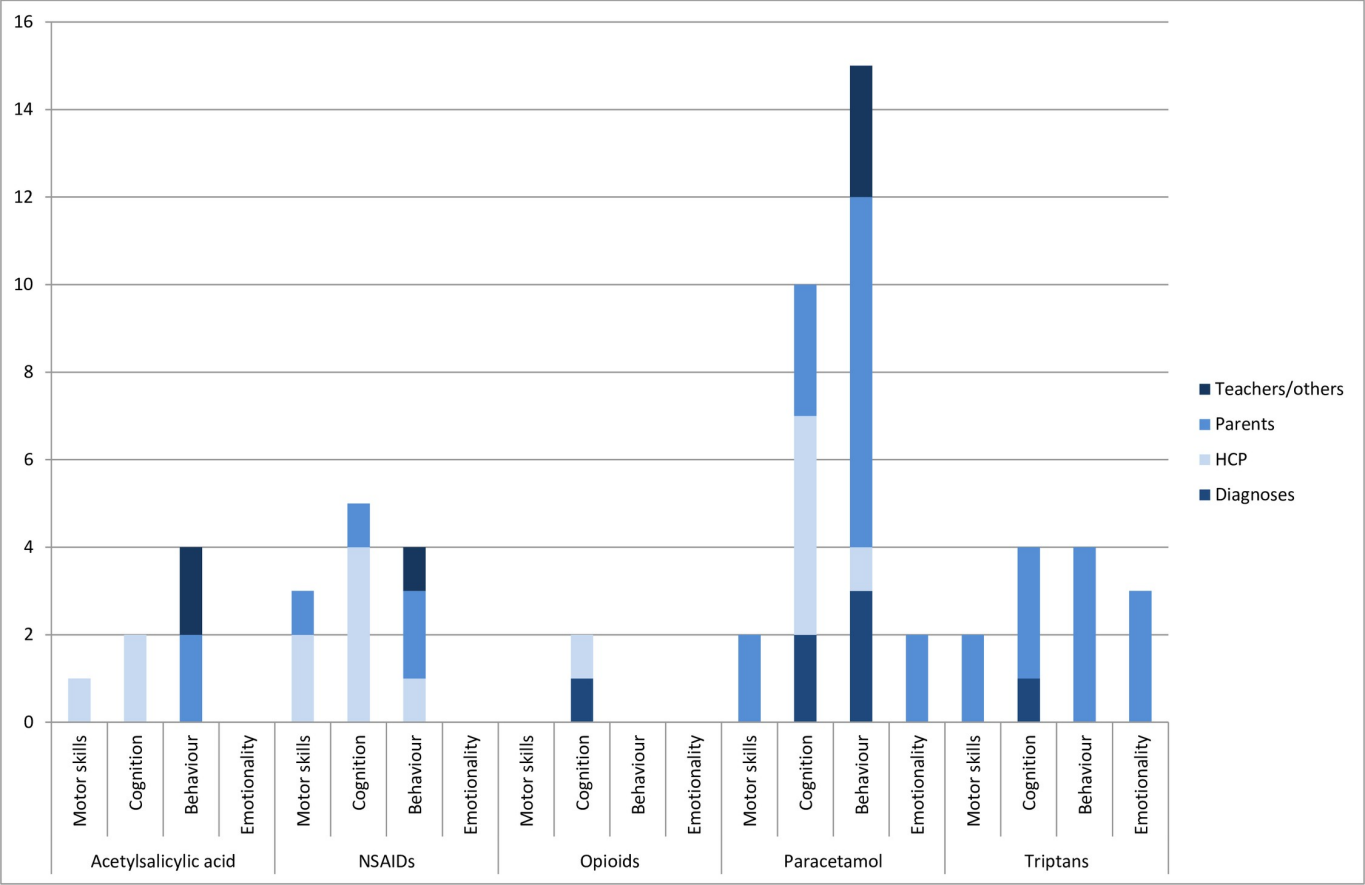
Domains of neurodevelopment evaluated and data sources used in medication safety papers on analgesics. Some papers had outcomes from more than one domain and assessments from more than one type of assessor. HCP: Health care professionals.

Due to a surge in papers since 2010, paracetamol was the most investigated analgesic with 18 papers [39,42,72,118–132]. Studies had follow-up between 18 months and 18 years (S3 Table). Most papers studied cognition and behaviour, with motor skills and emotionality investigated by two papers each (Fig 3). The most common data source was parent reporting.

### NSAIDs

Five papers investigated NSAIDs [120,133–136]. Three of the papers were more than ten years old. Children were followed from 6 months to 6 years. Three papers studied exposure to indomethacin for treatment of preterm contractions, so the populations consisted of a higher proportion of children born prematurely than the general population. Four papers reported assessments by health care professionals using psychometric instruments, two papers used parent reporting, and one teacher reporting [135]. The papers covered motor skills, cognition and behaviour domains of neurodevelopment, but not emotionality (Fig 3).

### Acetylsalicylic acid

Of the five papers on acetylsalicylic acid [39,127,130,137,138], three were more than 20 years old. Children were assessed from 4 to 11 years of age. Three papers used assessments by health care professionals using psychometric instruments. The newest paper used parent and teacher reporting [130]. One paper investigated the motor -, two the cognition -, and two the behaviour domain of neurodevelopment (Fig 3).

### Triptans

In four papers from the same research group [139–142], the same cohort of children was followed from 18 months to 5 years of age. All domains of neurodevelopment were assessed and assessors were the children’s parents (Fig 3). A fifth paper from a different context had follow-up until age 18 and assessed risk of ASD diagnosis [72].

### Analgesic opioids

Many papers have been written on use of illicit opioids, but for this review only analgesic opioids were included, yielding two papers [128,143] (Fig 3). In one paper, children’s language development was assessed at 3 years of age by the parents, and in the other, the risk of diagnoses of ASD and developmental delay were assessed in pre-schoolers.

### Validity and reliability of neurodevelopmental outcome measures

**Fig 4:**
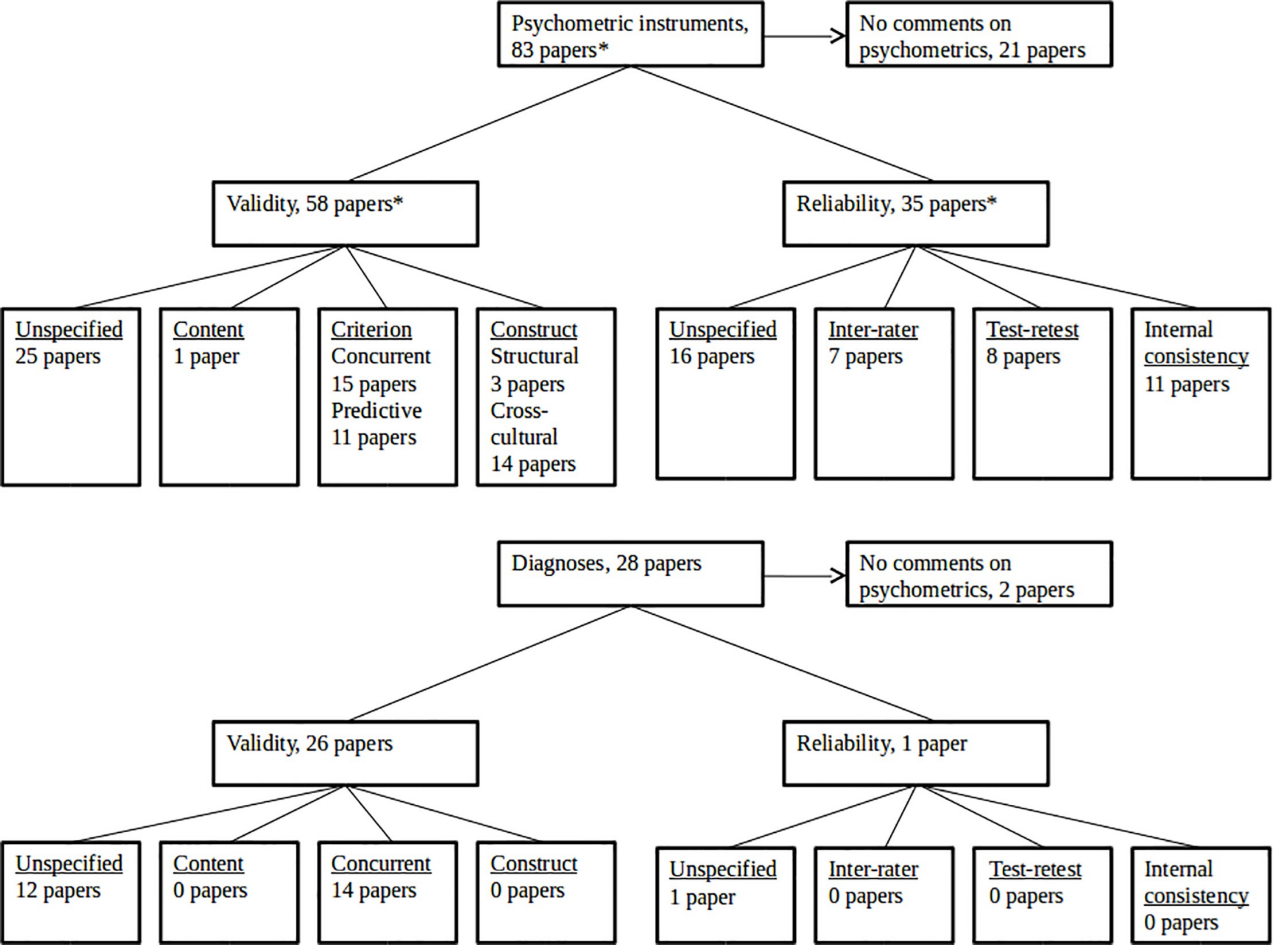
Psychometric properties of the neurodevelopment outcome measure mentioned in medication safety papers on psychotropics and analgesics. *Some papers commented on both validity and reliability, and those papers that commented on specific types of validity or reliability could comment on more than on type. One study used both diagnoses and psychometric instruments. Therefore the numbers add up to more than the total number of papers.

In 23 of the 110 eligible papers, no comment was made on either reliability or validity of any of the chosen outcome measures (Tables 1–3). The majority, 60 papers, commented qualitatively on reliability and/or validity of at least one of their chosen outcome measures, for instance by writing that the outcome measure was validated in their country. The remaining 27 papers also provided at least one quantitative measure of reliability and/or validity, such as Cronbach’s α for internal consistency. Of the papers that commented on specific types of validity and/or reliability, most, 28 papers, commented on concurrent validitywhile content validity was only mentioned in one paper (Fig 4, see Tables 1–3 for more detail). In 37 papers, it was not mentioned what type of validity the authors commented on, but it was rather stated, for example, that the outcome measure was well-validated. Reliability was mentioned in 36 papers, and of these 17 did not specify what type of reliability that was referred to. We have provided an overview of the reliability and validity of the used outcome measures based on information from the psychometric literature (See S4 Table). In the following, the reporting of validity and reliability of outcome measures will be described by medication group.

**Table 1:** Validity and reliability of outcome measures as reported by the authors of the study, papers on antidepressants.

		Psychometric properties of outcome measure	
Reference	Outcome	Reliability	Validity
**Assessment using psychometric instruments**			
**i. Assessment by health care professionals**			
***Infant (<2 years)***			
Suri 2011 [99]	BNBAS	Assessors were trained and certified to 0.90 reliability	Sensitive to medication effects. Limited normative base. Results that can be influenced by numerous subtle factors
Mortensen 2003 [81]	Boel test	Not mentioned	Test was performed at children’s home, not under standard settings in which the test was developed. Surroundings could be associated with both exposure and outcome
Batton 2013 [44]	Bayley Infant Neurodevelopment Screener	Not mentioned	Validated using clinical and normative standardisation samples
Weikum 2013a [101]	BSID, unspecified edition	Not mentioned	Not mentioned
Gustafsson 2018 [61]	BSID-II	Administrators had completed several practice administrations with repeated feedback prior to engaging in study administration of the BSID	Not mentioned
Oberlander 2004 [87]	BSID-II	Not mentioned	Not mentioned
Reebye 2002 [92]	BSID-II	Not mentioned	Not mentioned
Reebye 2012 [38]	BSID-II	Not mentioned	Not mentioned
Santucci 2014 [93]	BSID-II	Good reliability for infants from 1 to 42 months	Good concurrent validity for infants from 1 to 42 months
Austin 2013 [43]	BSID-III	BSID is considered gold standard. Good reliability and internal consistency	Good validity. Used US norms. BSID is more predictive, the older the child
Hanley 2013 [64]	BSID-III	Not mentioned	Not mentioned
Heikkinen 2002 [67]	Gesell Development scales	Psychometric properties not mentioned (Comment from review authors: Not mentioned which psychometric instrument is used, we have taken this information from the 2003 paper)	
Heikkinen 2003 [36]	Gesell Development scales	Not mentioned	Not mentioned
Johnson 2012 [73]	Infant Neurological International Battery	Interrater reliability 0.97, test-retest 0.95	Sensitivity 90%, specificity 83%, PPV 79%, NPV 93%
de Vries 2013 [53]	Psychomotor assessment according to Prechtl	Interrater reliability of general movement assessment is high (89% to 93%)	Abnormal general movement quality is highly predictive of later neurological impairment, but might not be sensitive enough to detect minor neurological sequelae. The Motor Optimality Score and the concurrent repertoire might be more revealing. Monotonous movement is related to minor neurological dysfunction in preterm infants
***Preschool (2–5 years)***			
Nulman 2002 [84]	BSID-II, Reynell developmental language scale, MSCA	Not mentioned	Not mentioned
Nulman 1997 [83]	BSID-II	Not mentioned	Not mentioned
Casper 2003 [49]	BSID-II	The two psychologist assessors were reliability certified on the BSID-II annually	Not mentioned
Batton 2013 [44]	BSID-III	Not mentioned	Not mentioned
Galbally 2011 [57]	BSID-III	Test-retest reliability for total score is 0.9 at 12 months	Regarded as the reference standard in the assessment of infant and toddler development. Only moderate predictive validity
Hurault-Delarue 2016 [69]	Compulsory medical exam	Not mentioned	Not mentioned
Schechter 2017 [94]	DAS	Fidelity checks every 6-months by a licensed clinical psychologist	Standardised, age-normed, well-validated measure of cognitive ability
Johnson 2016 [74]	DAS-II, Test of early language development, 3^rd^ edition (TELD-3)	Not mentioned	DAS-II: Measure is normalised. TELD-3: Not mentioned
Galbally 2015 [58]	Movement ABC, WPPSI-III	WPPSI, see validity. Motor ABC, reliability not mentioned	WPPSI is regarded as the gold standard for assessing cognitive ability. Motor ABC, validity not mentioned
Mattson 2002 [79]	WPPSI-R	Not mentioned	Not mentioned
***School child (6–12 years)***			
Nulman 1997 [83]	Reynell developmental language scale, MSCA	Not mentioned	Not mentioned
El Marroun 2017 [55]	SON-R (shortened), NEPSY-II	SON-R: Reliability was 0.73 for Mosaics and 0.71 for Categories. NEPSY-II: Not mentioned	SON-R: Validated in Dutch. Correlation to full scale, r = 0.86. NEPSY-II: Not mentioned
Hermansen 2016 [68]	NEPSY-II (shortened), WPPSI-R	Not mentioned	Not mentioned
Nulman 2012 [85]	WPPSI-III	Not mentioned	Child IQ levels were considerably higher than the normative mean of the general population. High IQs may reflect the Flynn effect. Mothers who contacted Motherisk may have had higher IQs than those who did not. Canadian norm for full-scale IQ is five points higher than the usual norm
Nulman 2015 [86]	WPPSI-III	Not mentioned	Not mentioned
***Adolescent (13–18 years)***			
Mattson 2002 [79]	WISC-III	Not mentioned	Not mentioned
**ii. Assessment by parents**			
***Infant (<2 years)***			
Brandlistuen 2015 [46]	CBCL	Reliability was adequate, Cronbach‘s α 0.62	The subset of items used in the MoBa study was found to be representative, with a correlation of 0.92 with the full scale
Reebye 2002 [92]	Early infant temperament questionnaire	Not mentioned	Not mentioned
Netsi 2015 [82]	Infant Characteristic Questionnaire, Brief Infant Sleep Questionnaire	Measures are reliable	Questionnaires relied heavily on maternal report and are subject to maternal bias
Nulman 1997 [83]	Toddler temperament scale	Not mentioned	Not mentioned
Nulman 2002 [84]	Toddler temperament scale	Not mentioned	Not mentioned
***Preschool (2–5 years)***			
Handal 2016a [62]	ASQ	Not mentioned	Validation study was done. For gross motor the estimated Spearman correlation was 0.25 with Mullen scores. For fine motor the correlation was 0.40
El Marroun 2017 [55]	BRIEF	Good test-retest reliability	Good content validity
Galbally 2015 [58]	CBCL, BRIEF	CBCL, see validity. BRIEF, reliability not mentioned	CBCL is reliable, valid and widely used. BRIEF, validity not mentioned
Brandlistuen 2015 [46]	CBCL	Reliability was adequate, Cronbach‘s α 0.74	The subset of items used in the MoBa study was found to be representative, with a correlation of 0.92 with the full scale
Hanley 2015 [65]	CBCL	Not mentioned	Maternal report poses some risk of differential outcome reporting
Johnson 2016 [74]	CBCL	Not mentioned	CBCL is not a diagnostic tool, but children scoring high on the PDD subscale are more likely to evidence behaviours commonly associated with ASD
Misri 2006 [80]	CBCL	Test-retest reliability r = 0.85, interrater reliability r = 0.61	Not mentioned
Nulman 2002 [84]	CBCL	Not mentioned	Not mentioned
Oberlander 2007 [88]	CBCL	Not mentioned	Widely used and well validated. Close relationship between maternal depression and maternal report of child behaviour. Increased level of aggression could represent lower maternal thresholds for tolerating preschool behaviour
Oberlander 2010 [89]	CBCL	Not mentioned	Widely used and well validated
Lupattelli 2018 [76]	CBCL, EAS	CBCL: Not mentioned in the article, but in the supplement internal consistency is reported quantitatively for each subscale. EAS: Moderate internal consistency of Norwegian version. In the supplement internal consistency is reported quantitatively for each subscale	Due to parent reporting misclassification cannot be ruled out, but was probably non-differential. CBCL: Widely used and validated. Predictive validity for adolescent psychiatric disorders showed a sensitivity of 0.71 and a specificity of 0.92. EAS: The short version used was highly correlated to full scale
Handal 2016b [63]	Intelligibility/Complexity of 3-year-old Children’s Utterances	Not mentioned	Validation study showed that children categorised by their mothers as having no language delay achieved a higher score on the communication domain of the Vineland Adaptive Behavior Scale than the children categorised as having severe language delay by mothers (Comment from review authors: Vinelands is a structured interview of parents by the HCP)
Skurveit 2014 [96]	Intelligibility/Complexity of 3-year-old Children’s Utterances	Not mentioned	Parental self-report is generally a good measure of early expressive vocabulary, especially for severe language delay. The validity of the language grammar rating scale has been described earlier
Pedersen 2013 [90]	SDQ	Not mentioned	Validated in Danish. Not intended to predict underlying psychiatric disorder. Relatively low sensitivity based on single informants
***School child (6–12 years)***			
Hutchison 2019 [70]	BRIEF	Not mentioned	Maternal perception of her child may have been negatively affected by depression, yet study using performance based measures of executive function found similar results
Hermansen 2016 [68]	CBCL	See validity	Widely used and standardised. Excellent reliability and validity
Nulman 1997 [83]	CBCL	Not mentioned	Not mentioned
Nulman 2012 [85]	CBCL, CPRS	Not mentioned	Maternal perception of her child may have been negatively affected by depression and its associated anxiety and stress
Nulman 2015 [86]	CBCL, CPRS-R	Not mentioned	Severity of maternal depression may influence responses when filling out questionnaires. Bias minimised as mothers evaluate both her exposed and unexposed children simultaneously (Comment from review authors: the mother still knows that one child was exposed and the other was not)
El Marroun 2014 [54]	CBCL and Social responsiveness scale (SRS)	Correlations between the CBCL measurements at different ages fell in the expected range, based on a mean correlation (r = 0.60)Crohnbach’s α indicated high inter-item reliability for the SRS (a = 0.79)	CBCL: Validated in Dutch. Good predictive validity to identify preschoolers at risk of autism spectrum disorder. Scales could not be normalised, so scores were dichotomised. The SRS correlated well with the pervasive developmental problems scale of the CBCL, r = 0.59
Hanley 2015 [65]	HBQ-P	See validity	Maternal report poses some risk of differential outcome reporting. The HBQ-P has strong psychometric properties
Weikum 2013b [102]	HBQ-P	See validity	The HBQ-P has strong psychometric properties. Mental health scales discriminate groups of children with and without signs of early psychopathology
Grzeskowiak 2016 [60]	SDQ	Not mentioned	Validated in Danish. Excellent discrimination for the identification of emotional (AUC 0.80) and behavioural (AUC 0.89) disorders
**iii. Assessment by teachers/others**			
***Preschool (2–5 years)***			
Johnson 2016 [74]	CBCL (other caregiver)	Not mentioned	CBCL is not a diagnostic tool, but children scoring high on the PDD subscale are more likely to evidence behaviours commonly associated with ASDs
Misri 2006 [80]	CBCL (teacher)	Test-retest reliability r = 0.85, interrater reliability r = 0.61	Not mentioned
Oberlander 2007 [88]	CBCL (teacher)	Not mentioned	Widely used and well validated. Close relationship between maternal depression and maternal report of child behaviour. Increased level of aggression could represent lower maternal thresholds for tolerating preschool behaviour
**Assessment using medical diagnosis**			
Boukhris 2017 [34]	ADHD	Not mentioned	Diagnosis defined from hospital diagnosis or redemption of a prescription for ADHD medication. Diagnoses of ADHD in the cohort were not validated. Sensitivity analysis on children with a diagnosis confirmed by neurologists and psychiatrists was consistent with those of the main analyses
Figueroa 2010 [56]	ADHD	Not mentioned	ADHD identified from diagnoses or treatment from practice, not from any formalised test or direct observation. This could lead to false-positive and false-negative errors. The young age when ADHD was diagnosed represents a limitation. Possible that only the most severe cases of ADHD were identified or that other behavioural problems that resemble ADHD are included
Laugesen 2013 [37]	ADHD	Not mentioned	Detection bias could have led to an overestimation of the association. Children with ADHD were identified based on hospital diagnoses and drug prescriptions. Patients with ADHD diagnosed by private psychiatrists or general practitioners and not prescribed drug treatment would be misclassified as not having ADHD
Man 2017 [78]	ADHD	Not mentioned	The registry contains information from publicly funded healthcare medical records. Does not include data from private medical practitioners or hospitals
Castro 2016 [50]	ASD and ADHD	Not mentioned	ICD-9 codes have a high sensitivity and specificity for ASD and ADHD versus generally healthy control. Case definition previously validated and included scores from autism diagnostic observation scale
Clements 2015 [51]	ASD and ADHD	Not mentioned	For ASD diagnosis in registry, sensitivity was 1.00, specificity 0.91. For ADHD, sensitivity was 0.84, specificity 0.90
Sujan 2017 [98]	ASD and ADHD	Not mentioned	Previous research has validated the diagnoses in used registries. Associations were estimated excluding offspring with diagnoses before age 2 years to address concerns about validity of early neurodevelopmental diagnoses. This did not markedly alter results
Wibroe 2017 [103]	ASD and ADHD	Not mentioned	Not mentioned
Malm 2016 [77]	ASD, depression, anxiety, ADHD	Not mentioned	Quality of the registry has been validated and is good for psychiatric diagnoses
Liu 2017 [75]	ASD, F30-39, F40-49, F70-79, F90-99	Not mentioned	Cannot rule out detection bias, but similar associations were observed for all disorders, irrespective of age at onset. Cases were redefined as at least two hospital contacts for psychiatric disorders, but similar results were obtained
Boukhris 2016 [45]	ASD	Not mentioned	Diagnoses of ASD in the cohort were not validated. Sensitivity analysis on children with a diagnosis of ASD confirmed by neurologists and psychiatrists was consistent with those of the main analyses
Brown 2017 [48]	ASD	Not mentioned	ASD was defined as 2 or more outpatient diagnoses by either a paediatrician or psychiatrist, 1 or more diagnoses in hospital databases, after the age of 2 years. A similar definition using US insurance data had a positive predictive value of 87.4%
Croen 2011 [52]	ASD	Not mentioned	Validation study of diagnoses in the registry against the Autism Diagnostic Interview–Revised and the Autism Diagnostic Observation Schedule–Generic; 94% of cases met criteria for ASD on both instruments, and 100% on at least one. A full review of diagnostic information recorded in cohort medical records demonstrated that at least 90% of ASD cases in the cohort meet DSM-IV criteria
Gidaya 2014 [59]	ASD	Not mentioned	Register diagnosis of childhood autism was confirmed 94% of the time with an additional 3% classified with another ASD in validation study
Hviid 2013 [71]	ASD	Not mentioned	A previous study showed that 94% of children registered with autism spectrum disorder diagnoses met diagnostic criteria in chart review. Not all children in the study have been followed throughout childhood. Some may receive a diagnosis of ASD at older ages. Detection bias cannot be ruled out
Janecka 2018 [72]	ASD	Not mentioned	Not mentioned
Rai 2017 [91]	ASD	Not mentioned	Previous validation studies found high validity of the diagnoses recorded in the registers
Sorensen 2013 [97]	ASD, infantile autism	Not mentioned	The quality of the infantile autism diagnosis in the registry has been validated. 94% met the criteria for correct diagnosis
Viktorin 2017b [41]	ASD	Not mentioned	The ASD diagnoses in the register have previously been validated
Harrington 2014 [66]	ASD, developmental delay (DD)	Not mentioned	Diagnoses of ASD and DD were confirmed by trained clinicians using validated standardised instruments. Controls were screened and reclassified if appropriate
Brown 2016 [47]	Disorders of speech/language, motor skills, and scholastic skills	Not mentioned	Diagnostic data were based on specialised health services rather than primary care. Some proportion of children with mild dysfunction may have been missed
Simon 2002 [95]	Developmental delay of motor skills, developmental delay of speech	Not mentioned	Diagnosis required both a physician diagnosis and confirmation by a formal developmental evaluation. Examinations at outpatient paediatric visits are relatively crude screening measures. Use of these data may reduce bias in ascertainment of developmental delay, but it sacrifices sensitivity by limiting analyses to abnormalities detected during routine medical care
Viktorin 2017a [100]	Intellectual disability	Not mentioned	Children with ID without clinical care are not captured, so the prevalence estimate of ID in the study may be an underestimate of the true prevalence in the population. Detection bias cannot be ruled out

ADHD: Attention Deficit Hyperactivity Disorder, ASD: Autism Spectrum Disorder, ASQ: Ages and Stages Questionnaire, BNBAS: Brazelton Neonatal Behavoiral Assessment Scale, BRIEF: Behaviour Rating Inventory of Executive Function, BSID: Bayley Scales of Infant Development, CBCL: Child Behaviour Checklist, CPRS: Conners Parent Rating Scale, DAS: Differential ability scales, EAS: Emotionality, Activity, Sociability Temperament Survey, HBQ-P: MacArthur Health and Behaviour Questionnaire, MSCA: McCarthy’s scales of children’s abilities, SDQ: Strengths and Difficulties Questionnaire, SON-R: Snijders–Oomen Niet-verbale intelligentie Test–Revisie, WISC: Wechsler Intelligence Scale for Children, WPPSI: Wechsler Preschool and Primary Scale of Intelligence.

**Table 2:** Validity and reliability of outcome measures as reported by the authors of the study, papers on psychotropics except antidepressants.

			Psychometric properties of outcome measure	
Reference	Exposure	Outcome measure	Reliability	Validity
**Assessment using psychometric instruments**				
**i. Assessment by health care professionals**				
***Infant (<2 years)***				
Platt 1989 [114]	Antipsychotics	BSID	Not mentioned	Measures obtained at different ages may differ in sensitivity. The categorical measure reported was not used in original examination, but derived by combining relevant motor items which showed some variance in study population, and dichotomising resulting scores to maximise drug effects
Peng 2013 [113]	Antipsychotics	BSID-III	Not mentioned	The scale is widely used and has potential to provide clinically relevant information on early neurodevelopment
Johnson 2012 [73]	Antipsychotics	Infant Neurological International Battery	Interrater reliability 0.97, test-retest 0.95	Sensitivity 90%, specificity 83%, PPV 79%, NPV 93%
Mortensen 2003 [81]	Antipsychotics, anxiolytics	Boel test	Not mentioned	Test was performed at children’s home, not under standard settings in which the test was developed. Surroundings could be associated with both exposure and outcome
Oberlander 2004 [87]	Anxiolytics (clonazepam combined with SSRI)	BSID-II	Not mentioned	Not mentioned
Reebye 2002 [92]	Anxiolytics (clonazepam combined with SSRI)	BSID-II	Not mentioned	Not mentioned
Reebye 2012 [38]	Anxiolytics (clonazepam combined with SSRI)	BSID-II	Not mentioned	Not mentioned
Viggedal 1993 [40]	Anxiolytics	Griffiths’ mental development scale I, Neuropsychological assessment	Griffiths’: Not mentioned. Neuropsychological assessment: Deviations from normal activity and attention was diagnosed when present at two independent observations	Griffiths’: The high IQ in the reference group is considered normal as the average IQ nowadays is about 110. Neuropsychological assessment: Not mentioned
Gidai 2008a [105]	Anxiolytics (alprazolam)	Hungarian development test, Behavioural style questionnaire	Not mentioned	Not mentioned
Gidai 2008b [106]	Anxiolytics (medazepam)	Hungarian development test, Behavioural style questionnaire	Not mentioned	Not mentioned
Gidai 2008c [107]	Anxiolytics (chlordiazepoxide)	Hungarian development test, Behavioural style questionnaire	Not mentioned	Not mentioned
Timmermann 2008b [112]	Anxiolytics (meprobamate)	Hungarian development test, Behavioural style questionnaire	Not mentioned	Not mentioned
Laegreid 1992 [108]	Anxiolytics	Touwen Neurologic Assessment, Clinical neurologic assessment	Not mentioned	The test showed a fair differentiation and an evident developmental sequence
Petik 2008a [115]	Hypnotics (glutethimide)	Hungarian development test, Behavioural style questionnaire	Not mentioned	Not mentioned
Petik 2008b [116]	Hypnotics (Amobarbital)	Hungarian development test, Behavioural style questionnaire	Not mentioned	Not mentioned
Timmermann 2008a [117]	Hypnotics (barbital, hexobarbital, butobarbital)	Hungarian development test, Behavioural style questionnaire	Not mentioned	Not mentioned
***Preschool (2–5 years)***				
Hurault-Delarue 2016 [69]	Antipsychotics, anxiolytics and hypnotics	Compulsory medical exam	Not mentioned	Not mentioned
Schechter 2017 [94]	Antipsychotics, anxiolytics, hypnotics	DAS	Fidelity checks were done approximately every 6-months by a licensed clinical psychologist	The DAS is a standardised, age-normed, well-validated measure of cognitive ability
Hartz 1975 [35]	Anxiolytics (meprobamate, chlordiazepoxide)	Stanford Binet Intelligence Scale	Not mentioned	Not mentioned
Mattson 2002 [79]	Anxiolytics	WPPSI-R	Not mentioned	Not mentioned
***School child (6–12 years)***				
Platt 1989 [114]	Antipsychotics	Paediatric neurologic assessment	Not mentioned	Not mentioned
***Adolescent (13–18 years)***				
Mattson 2002 [79]	Anxiolytics	WISC-III	Not mentioned	Not mentioned
**ii. Assessment by parents**				
***Infant (<2 years)***				
Reebye 2002 [92]	Anxiolytics (clonazepam combined with SSRI)	Early Infancy Temperament Questionnaire	Not mentioned	Not mentioned
***Preschool (2–5 years)***				
Lupattelli 2019 [109]	Anxiolytics, hypnotics	ASQ, CPRS-R	ASQ: Internal consistency was 0.6 to 0.7. CPRS-R: Internal consistency 0.9	Widely used and validated. In the supplement, the authors present associations between ASQ and diagnosis of motor delay (Beta 1.96 to 4.48) or language impairment (Beta 2.06 to 2.45), and between CPRS-R and parental ADHD symptoms (Beta 0.12 to 0.30 for paternal symptoms and 0.41 to 0.76 for maternal symptoms)
Brandlistuen 2017 [104]	Anxiolytics, hypnotics	CBCL (shortened)	Not mentioned	Validated, representative of full scale, the domains predict later psychopathology. High factor loadings
Misri 2006 [80]	Anxiolytics (clonazepam combined with SSRI)	CBCL	Test-retest reliability r = 0.85, interrater reliability r = 0.61	Not mentioned
Odsbu 2015 [110]	Anxiolytics	Intelligibility/Complexity of 3-year-old Children’s Utterances	Not mentioned	Parental self-report is a good measure of early expressive vocabulary, especially for severe language delay. Validity of the language grammar rating scale in the cohort has been described previously
***School child (6–12 years)***				
Radojčić 2017 [111]	Anxiolytics	CBCL	Cronbach's alphas for all scales were the same in 6 year-old children and in older children, indicating that problems were reliably measured in children older than 6 years of age	Validated in the Netherlands
**iii. Assessment by teachers/others**				
***Preschool (2–5 years)***				
Misri 2006 [80]	Anxiolytics (clonazepam combined with SSRI)	CBCL	Test-retest reliability r = 0.85, interrater reliability r = 0.61	Not mentioned
***School child (6–12 years)***				
Radojčić 2017 [111]	Anxiolytics	CBCL	Cronbach's alphas for all scales were the same in 6 year-old children and in older children, indicating that problems were reliably measured in children older than 6 years of age	Validated in the Netherlands
**Assessment using medical diagnosis**				
Figueroa 2010 [56]	Anxiolytics	ADHD	Not mentioned	ADHD identified from diagnoses or treatment from practice, not from any formalised test or direct observation. This could lead to false-positive and false-negative errors. The young age when ADHD was diagnosed represents a limitation. It is possible that only the most severe cases of ADHD were identified or that other behavioural problems that resemble ADHD are included
Janecka 2018 [72]	Lithium	ASD	Not mentioned	Not mentioned

ADHD: Attention Deficit Hyperactivity Disorder, ASD: Autism Spectrum Disorder, ASQ: Ages and Stages Questionnaire, BSID: Bayley Scales of Infant Development, CBCL: Child Behaviour Checklist, CPRS:R: Conners Parent Rating Scale, revised, DAS: Differential ability scales, NPV: Negative predictive value, PPV: Positive predictive value, WISC: Wechsler Intelligence Scale for Children, WPPSI: Wechsler Preschool and Primary Scale of Intelligence.

**Table 3:** Validity and reliability of outcome measures as reported by the authors of the study, papers on analgesics.

			Psychometric properties of outcome measure	
Reference	Exposure	Outcome measure	Reliability	Validity
**Assessment using psychometric instruments**				
**i. Assessment by health care professionals**				
***Infants (<2 years)***				
Salokorpi 1996 [136]	Indomethacin	Autti-Rämö neurodevelopmental test battery	Not mentioned	Standardised for Finnish children
Amin 2008 [134]	Indomethacin	BSID-II, mental development index	Not mentioned	Not mentioned
Al-Alaiyan 1996 [133]	Indomethacin	Gesell development scales, revised	Not mentioned	Not mentioned
Avella-Garcia 2016 [118]	Paracetamol	BSID, unspecified ed.	Not mentioned in the paper, but in supplement. Cronbach’s α 0.70 (good to moderate)	Not mentioned
***Preschool (2–5 years)***				
Barr 1990 [137]	ASA	Items from Gross Motor Scale (University of Oregon Medical School), Gesell–and Bayley Scales, Wisconsin Fine Motor Steadiness Battery, and Halstead Reitan Neuropsychological Battery	Monthly reliability checks revealed good interrater reliability	For three variables, the best predictors were exam conditions. None of these measures are normally used with children this young
Klebanoff 1988 [138]	ASA	Stanford Binet Intelligence Scale	3 months test-retest, and in addition inter-rater, reliability was 0.83	Test focuses on verbal abilities. Highly correlated to school performance
Streissguth 1987 [39]	ASA	WPPSI	Not mentioned	IQ scores by age 4 years have a good predictive validity for later intellectual function
Avella-Garcia 2016 [118]	Paracetamol	McCarthy Scales of Children’s Abilities, CAST*	McCarthy Scale of Children’s Abilities: Cronbach’s α 0.90. CAST: Cronbach’s α 0.64 (good to moderate)	Only mentioned for CAST: Sensitivity 100%, specificity 97% for ASD
Bornehag 2018 [119]	Paracetamol	Swedish language development scale†	Not mentioned	Validated in Sweden
Liew 2016b [124]	Paracetamol	TEACh-5	The authors do not specify the reliability or validity but refers readers interested in psychometric properties to two papers on the psychometrics of the instrument	
Liew 2016c [125]	Paracetamol	WPPSI-R, shortened	Not mentioned	Full-scale IQ was derived from the selected items. Danish WPPSI-R norms were not available, so Swedish norms were used. Therefore the distribution of IQ scores in the sample does not have a mean of 100 and SD of 15
***Child (6–12 years)***				
Markovic 2019 [135]	NSAIDs	SON-R	Reliability of the tests used in the study was 0.73 and 0.71	Correlation to full scale was r = 0.86. Validated in Dutch
Laue 2019 [121]	Paracetamol	WISC-IV	Not mentioned	WISC is objective and less biased than parental report
**ii. Assessment by parents**				
***Infants (<2 years)***				
Vlenterie 2016 [42]	Paracetamol	Motor milestone questionnaire, ASQ, CBCL, EAS	Motor milestone is believed to be objective and therefore a reliable maternal report on motor development. ASQ: Not mentioned. CBCL: Not mentioned. EAS: The short form was as reliable and precise as the full scale	Motor milestones: Not mentioned. ASQ: Validated in Norway. CBCL and EAS: Parent-reported behaviour outcomes can suffer from differential misclassification
Wood 2016a [140]	Triptans	ASQ, CBCL, EAS	ASQ: The questions had excellent test–retest reliability and agreement between parents and professional examiners. CBCL: Not mentioned. EAS: In a Norwegian sample, internal consistency (α) within each scale ranged from 0.48 to 0.79	The ASQ is predictive of school performance. The short version has been validated in Norway and in young children. CBCL: The shortened CBCL has been validated in Norway and in young children. Parents are better reporters of externalising symptoms, whereas children are better reporters of internalising symptoms. EAS has been validated in children as young as those studied
***Preschool (2–5 years)***				
Markovic 2019 [135]	NSAIDs	CBCL	Internal consistency (α) 0.68	Good validity, validated in Dutch. The subscales had good fit in international studies in diverse societies
Brandlistuen 2013 [120]	NSAIDs and paracetamol	Motor milestone questionnaire, ASQ, CBCL, EAS	Maternal reports of gross motor milestone attainment have been reported to be highly reliable. ASQ: Not mentioned. CBCL: Not mentioned. EAS: The short form was as reliable and precise as the full scale	Motor milestones: Not mentioned. ASQ: Validated in Norway. Average factor loading 0.61 for fine motor and 0.75 for gross motor items, adequate reliability. Average factor loading 0.82 for communication, good reliability. (Comment from review authors: According to COSMIN principles, factor loadings are considered part of construct validity). CBCL: Correlation to CBCL full scale was 0.92. Average factor loading 0.58 for externalising and 0.52 for internalising behaviour scales, adequate reliability. EAS: Factor loading for emotionality 0.71, activity 0.68, for sociability 0.58, for shyness 0.69
Liew 2014 [122]	Paracetamol	SDQ	Reliable screening tool	With the cut-off used, the scale has high specificity for ADHD-like behaviours and 17% of children with problems on the SDQ have received a diagnosis of HKD
Liew 2016b [124]	Paracetamol	BRIEF	The authors do not specify the reliability or validity but refers readers interested in psychometric properties to two papers on the psychometrics of the instrument	
Skovlund 2017 [128]	Paracetamol and opioids	Intelligibility/Complexity of 3-year-old Children’s Utterances & ASQ	Not mentioned	Parental report is considered a valid measure and validation against clinical assessment has been described for the instrument in the cohort
Wood 2016b [141]	Triptans	CBCL	Not mentioned	The shortened CBCL has been validated in Norway. The domains used, have been shown to predict later psychopathology in children and adolescents
Wood 2016c [142]	Triptans	ASQ, EAS	Not mentioned	The ASQ is predictive of school performance. This short version has been validated in Norway. EAS: Not mentioned
Harris 2018 [139]	Triptans	ASQ, CBCL, EAS	Not mentioned in the article, but in the supplement internal consistency is reported quantitatively for each subscale	Not mentioned
***Child (6–12 years)***				
Stergiakouli 2016 [129]	Paracetamol	SDQ	SDQ is a validated and reliable screening instrument	
Tovo-Rodrigues 2018 [131]	Paracetamol	SDQ	Not mentioned	Validated for a Brazilian population and for the studied age group
Thompson 2014 [130]	Paracetamol and ASA	SDQ, CPRS:R-L (only for paracetamol)	SDQ has a test-retest reliability of 0.62 after 4 to 6 months. Internal consistencies of the subscales range from 0.62 to 0.75	Self-reported problem behaviour has been shown to be a more valid indicator of mental and physical health than parent-reported problems
Ruisch 2018 [127]	Paracetamol and ASA	Development and Well-Being Assessment	Inter-rater differences between maternal and teacher assessments could reflect different behaviours in different settings or bias in the assessment	Good validity
**iii. Assessment by teachers/others**				
***Preschool (2–5 years)***				
Avella-Garcia 2016 [118]	Paracetamol	California Preschool Social Competence Scale, ADHD, DSM-IV form list	California Preschool Social Competence Scale: Cronbach’s α 0.89. ADHD, DSM-IV form list: Cronbach’s α 0.90	Not mentioned
Liew 2016b [124]	Paracetamol	BRIEF	The authors do not specify the reliability or validity but refers readers interested in psychometric properties to two papers on the psychometrics of the instrument	
***Child (6–12 years)***				
Markovic 2019 [135]	NSAIDs	CBCL	Not mentioned	Not mentioned
Thompson 2014 [130]	Paracetamol and ASA	SDQ (children)	SDQ has a test-retest reliability of 0.62 after 4 to 6 months. Internal consistencies of the subscales range from 0.62 to 0.75	Self-reported problem behaviour has been shown to be a more valid indicator of mental and physical health than parent-reported problems
Ruisch 2018 [127]	Paracetamol and ASA	Development and Well-Being Assessment	Inter-rater differences between maternal and teacher assessments could reflect different behaviours in different settings or bias in the assessment	Good validity
**Assessment by medical diagnosis**				
Rubenstein 2019 [143]	Opioids	Developmental delay and ASD	Not mentioned	All children in the study were screened for ASD and had general developmental evaluations by a clinician. Children with positive screenings had comprehensive ASD evaluations
Janecka 2018 [72]	Triptans and paracetamol	ASD	Not mentioned	Not mentioned
Liew 2016a [123]	Paracetamol	Infantile autism and ASD	Not mentioned	Diagnoses of ASD were ascertained from the general and psychiatric hospital registries in Denmark using standardised diagnostic criteria. Diagnoses of infantile autism in the psychiatric registry have previously been shown to have high validity
Liew 2014 [122]	Paracetamol	HKD	Not mentioned	Children who received diagnoses solely prior to 5 years of age were not considered as having HKD due to higher diagnostic uncertainty at younger ages. 79% of all children diagnosed with HKD had redeemed medications at least twice
Liew 2019 [126]	Paracetamol	ADHD	Diagnoses of ADHD were ascertained through maternal report. This method is reliable	In a validation study, all girls with maternal report of ADHD scored above 90% on ADHD Rating Scale-IV, as did 64% of boys. ADHD prevalence in the study was comparable to estimates by Centers for Disease Control and Prevention
Ystrom 2017 [132]	Paracetamol	ADHD	Not mentioned	Children who received diagnosis before 3 years of age were excluded. Cases were children with one or more diagnoses from specialist health care

*Structured interview of parents by the health care professional

† Mixture of parental questionnaires and nurse observation

ADHD: Attention Deficit Hyperactivity Disorder, ASA: Acetylsalicylic acid, ASD: Autism Spectrum Disorder, ASQ: Ages and Stages Questionnaire, BRIEF: Behaviour Rating Inventory of Executive Function, BSID: Bayley Scales of Infant Development, CAST: Childhood Autism Spectrum Test, CBCL: Child Behaviour Checklist, CPRS:R-L: Conners’ Parent Rating Scale, revised, long format, EAS: Emotionality, Activity, Sociability Temperament Survey, HKD: Hyperkinetic disorder, SDQ: Strengths and Difficulties Questionnaire, SON-R: Snijders–Oomen Niet-verbale intelligentie Test–Revisie, TEACh-5: Test of everyday attention, 5 years, WISC: Wechsler Intelligence Scale for Children, WPPSI: Wechsler Preschool and Primary Scale of Intelligence.

### Antidepressants

In 66 papers on antidepressants, 53 had comments on validity and/or reliability of at least one of their outcome measures. It was most common to report on concurrent validity, done in 22 papers, or not to specify the type of validity that was commented on, which was the case for another 22 papers. One paper mentioned content validity, and none of the papers mentioned structural validity.

### Anxiolytics

Ten of the 20 papers on anxiolytics had comments on validity and/or reliability of at least one of their outcome measures. The type of validity that was mentioned most often was construct validity in the form of correlation to the full scale of the instrument. None of the papers mentioned content validity.

### Antipsychotics

Of seven papers on antipsychotics, five had comments on validity and/or reliability of at least one of their outcome measures. However, four of these did not mention what type of validity their comment regarded. None of the papers mentioned construct validity, content validity or internal consistency.

### Hypnotics and sedatives

Half of the six papers on hypnotics and sedative commented on validity and/or reliability of at least one of their outcome measures. No type of validity or reliability was mentioned in more than one paper. None of the papers mentioned content validity, inter-rater–or test-retest reliability.

### Paracetamol

All but one of the 18 papers on paracetamol had comments on validity and/or reliability of at least one of their outcome measures. Eight papers did not specify what type of validity the comment regarded. None of the papers mentioned content validity.

### NSAIDs

Three of five papers on NSAIDs had comments on validity and/or reliability of at least one of their outcome measures. All three mentioned cross-cultural validity (a subtype of construct validity), none mentioned criterion validity, content validity, inter-rater–or test-retest reliability.

### Acetylsalicylic acid

All five papers commented on validity and/or reliability of at least one of their outcome measures. In three papers, the type of validity was not specified, yet four of the papers commented on a specified type of reliability. None of the papers mentioned construct validity or content validity.

### Triptans

Of five papers on triptans, four had comments on validity and/or reliability of at least one of their outcome measures. Most frequently mentioned was predictive validity and cross-cultural validity. None of the papers mentioned structural validity or content validity.

### Opioids

Both papers on opioids commented on the validity of at least one of their outcome measures, one on concurrent validity, the other did not specify type of validity. None of the papers commented on reliability, construct validity or content validity.

## Discussion

In the 110 papers on neurodevelopment after prenatal exposure to psychotropics (66 papers on antidepressants, 27 papers on other psychotropics) or analgesics (29 papers) identified from a systematic review of the literature, 47 different psychometric instruments and 13 different diagnostic categories were used to measure neurodevelopment. Twenty-three papers did not mention the reliability or validity of any of the neurodevelopment outcome measures. Among the papers that did mention psychometric properties, 37 papers did not specify on what type of validity they were commenting.

### Strengths and limitations

Strengths of this review include a comprehensive search in six different databases, an interdisciplinary research team, compliance with PRISMA guidelines, and assessment of study eligibility and quality, as well as data extraction, done in double.

Limitations include that only published papers in predefined languages were included in the review, though no papers in other languages that fulfilled the remaining eligibility criteria turned up in our search. Further, search strategy could have been optimised by inclusion of the names of maternal illnesses that are indications for use of the studied medications. Publication bias could not be ruled out for most of the medication groups. Although this may affect the interpretation of effect sizes and risks of using the medications in pregnancy, we consider it unlikely to affect how authors report the psychometric properties of the instruments they use. Finally, the reviewers were not blinded to study authors when assessing study eligibility and quality. This could be a limitation as many of the included studies were done in our research group. However, the use of predefined criteria for eligibility and quality assessment should decrease the risk of bias.

### Points for consideration

There are many factors influencing study design in this area, however a lack of current consensus leads to incompatible data across studies which undoubtedly prolongs the period of time it takes to confirm safety or risks to the foetus. Based on the systematic literature review and our experiences as researchers and clinicians, we provide five points that should be considered for the conduct and interpretation of studies on maternal prenatal use of medications, and child neurodevelopment.

#### A wide variety of outcomes must be assessed to establish neurodevelopmental safety

The human brain is complex and its functions are diverse. Whilst there is comorbidity between neurodevelopmental disorders [24,144], all domains of neurodevelopment (e.g. IQ, language efficiency, attention etc.) are important for children’s daily living, and it should be considered that they may be differentially impacted upon by teratogen exposure. Therefore a call for complete consensus on how to measure neurodevelopment, for instance by selecting one domain of neurodevelopment as the priority in medication safety research, is not reasonable. A complete consensus on choice of outcome measure may also be difficult to obtain, as a single outcome measure does not exist which can reliably measure all diverse aspect of neurodevelopment, and different populations may require different measures due to variables such as age and geographical region. All outcome measures have strengths and weaknesses that we will elaborate on below, and validation of psychometric instruments and diagnoses is done using other psychometric instruments or diagnostic categories as references, making the discussion of validity relativistic. Despite these challenges, to be able to build on each other’s research and pool results in meta-analyses, it is necessary that some agreement should be reached regarding a core outcome set for teratology studies investigating neurodevelopment, so studies assessing the same domain of neurodevelopment and using the same data source also would use compatible outcome measures and report results in a uniform manner.

#### Previous literature, both animal and human, should inform choice of outcomes to be measured

In order to bring about a more uniform approach to the study of central nervous system acting medications in pregnancy and the potential impact on the developing brain, new research should select primary outcomes guided by previous literature from both animal and human studies. Reviews of the pre-clinical research, or knowledge of the literature on in-vitro or animal studies, are necessary along with those of the already available human literature. Prenatal exposure to the antiepileptic medication valproate demonstrates this point. Despite early case reports noting impaired human neurodevelopment alongside major congenital malformations, Pregnancy Registers around the world were established to assess major congenital malformation risk but not neurodevelopment [145]. Further, an early review of the pre-clinical research data would have added further weight for the requirement to study neurodevelopment following valproate exposure with the same gravitas as major congenital malformations. As early as 1996 an association between valproate and ASD like behaviours was noted [146], whilst ASD in exposed children was not the focus of a prospective investigation until much later [22].

#### Data sources have different strengths and weaknesses and should complement each other

Standardised psychometric instruments completed blinded to exposure status by health care professionals are considered the gold standard to assess certain areas of neurodevelopment, e.g. Bayley Scales of Infant Development to assess early development [57], or Wechsler Preschool and Primary Scale of Intelligence to assess child IQ [58]. Such clinical assessments are often detailed, providing comprehensive information on a number of neurodevelopmental outcomes. A strength of assessment by health care professionals for research purposes is that blinding of the assessors can reduce unconscious bias. It is therefore important that blinding takes place when possible and that authors state whether the assessors were blinded when reporting a study. In this review, blinding status was reported in half of the 52 papers that used assessments by health care professionals (S5–S7 Tables). A limitation to the use of licenced psychometric instruments is costliness, training of assessors, and the amount of time spent on each assessment. In addition, families will be required to give up time for the assessment. Therefore we see in this review that studies using psychometric instruments completed by health care professionals often are small in size and in some cases only powered to detect the largest of group differences. In small studies, where random error may impact results, it is important to report on the reliability of the outcome measure used. For all psychometric instruments, the concurrent and predictive validity should be considered, as well as content validity to enable clinicians and researchers to determine whether the symptoms or traits evaluated by the scales are in fact clinically relevant to the specific population being investigated.

There are limited opportunities for health care professionals to measure child behaviour and emotionality in addition to cognitive development, as a clinic will rarely provide natural settings to observe these domains. In addition, emotionality is dependent on both situation and relation to the assessor. Therefore these domains will often rely on parent, teacher, or child reporting. In settings where few children are looked after in day care centres, parents will often be the only ones who see preschool children on a sufficiently regular basis to provide assessments of behaviour or emotionality.

Often less burdensome for families, and, in some cases, available to research groups that do not have access to licenced psychologists, psychometric instruments completed by parents or teachers can be used in large samples. There are two main weaknesses in parent reporting. One is the lack of blinding to exposure status, which is particularly important for medications that have received media attention as having unfavourable effects on the developing brain. The other is specific to psychotropics, namely distortion bias, the influence of maternal mood on the assessment. Whether maternal mood will affect reporting is at present disputed [64]. Some studies indicate that mothers with no emotional disorders will underrate child problems, while mothers with emotional disorders will overrate [147,148], whereas other studies do not find clinically significant effects of maternal emotional disorder [149,150].

Teacher reporting may be blinded to exposure status. However, the expectations of children in a classroom setting may be different from what is expected from children elsewhere. One review concluded that teacher reporting results in a higher prevalence of ADHD than if the disease is classified according to diagnostic criteria [151]. In addition, not all domains of neurodevelopment may be assessed with equal ease in a classroom setting. Hence in a meta-analysis of 119 studies, parent reporting of emotional problems correlated better than teacher reporting with children’s own assessments [152]. When parent and teacher reporting only show moderate correlations, it is important to consider that they represent assessment of the child in very different settings. Some children have problems at school that are not present in the home-environment. Hence one assessment is not necessarily more correct than the other. In both parent and teacher reporting, the concurrent and predictive validity should be considered, as well as content validity to enable clinicians and researchers to determine whether the symptoms or traits evaluated by the scales are in fact clinically relevant problems.

When using the presence or absence of a diagnosis (i.e. ASD or ADHD) to assess neurodevelopment, we clearly only examine the most affected individuals. In countries or regions with health registries, this data source is comparatively cheap and fast. However, detection bias is always possible, and blinding of assessors rating the outcome is not possible. For instance, women exposed to a suspected teratogen or women with a history of mental illness may be more likely to get their children examined for mental illnesses. Further, the presence or absence of diagnoses can be a somewhat crude measure and may differ by region, country, or version of the diagnostic manual in their criteria. Often registries contain data from public secondary services, wherefore children managed in primary care or in private hospitals may be misclassified. In addition, not all children with clinically relevant problems will fulfil the diagnostic criteria for a certain disorder. As an example, the known developmental neurobehavioural teratogen valproate increases the prevalence of ASD in children from 1.8% in general population controls to 8–15% in prenatally exposed children [22]. However, the clinical picture in these cases of ASD is atypical [153]. It is possible that the medications we investigate will increase the risk of a syndrome that is not caught by common diagnostic criteria. Lessons learned from congenital malformations show that minor deviations should not be overlooked as they might be part of specific diagnosis [154]. When using the presence or absence of diagnoses as a neurodevelopmental outcome, the authors should address both the validity of the diagnostic criteria, and the validity of the recording of diagnoses in the registries the data stem from. The specificity of diagnoses from registries can be increased by requiring that a diagnosis should be present at least twice in a child’s medical records before the child is considered as having that diagnosis [155]. This will exclude the instances where a child is evaluated to rule out a diagnosis.

As the different data sources have different strengths and weaknesses, they can be used to complement each other. So far, a minority of studies have used a mixture of data sources, and only one used assessment by both diagnoses and psychometric instruments [122]. Future studies should to a greater extent use more than one data source to measure neurodevelopment if the expertise within the research group allows. For an example of how this could be done, see the paper by Liew and colleagues [122].

In meta-analyses, the use of different data sources can be challenging. Currently, review authors differ on whether to combine outcome measures from different data sources in meta-analyses [16,156–158], or summarise the evidence qualitatively [15,159]. Until a consensus is reached on which outcome measures to use and which to combine, we would like to caution over the combining of data from different research methodologies. Further, given the diversity of neurodevelopmental outcomes and their measurement there is a requirement of in-depth knowledge of the various outcome measures, as they are often based on different constructs reflecting different neurodevelopmental domains at different ages. Finally, standard approaches to meta-analysis of data including publication bias and heterogeneity in outcome measures between studies should be taken into account per outcome using standardised methods such as funnel plots.

#### The outcome measure should be age appropriate

Standardised tests and questionnaires are often validated for a certain age group. If the outcome measure is to be used in a different age group, it should first be validated for use in that age group [19]. Researchers using diagnoses as outcomes should be aware that certain diagnoses are not valid below a certain age. For example, the American Academy of Pediatrics recommends that children should be 4 years old before DSM-IV diagnostic criteria of ADHD can be used [144]. Children should not be considered as having a diagnosis, if they only have a diagnosis recorded at an age where it is considered implausible that a correct diagnosis can be made. Length of follow-up should be guided by the average age at diagnosis for the specific disorder in the country where the research is carried out.

When planning new research on medication safety for neurodevelopment, it should also be considered that brain development continues into early adulthood [25], and that some difficulties in certain cognitive domains will not be detectable until the teenage years, when more complex cognitive processing is required [13]. For example, very different levels of inhibition or reasoning ability are expected from a 3-year-old and a 13-year-old. Longer follow-up would also allow investigation into mental disorders that may have developmental origins, but that have their onset in adolescence, such as schizophrenia. Another way to take into account the continuing development of the child brain is to investigate trajectories of development. This method is common in psychology [160], and could be employed in medication safety studies as well, if children are assessed at several points in time.

#### Reliability and validity of the outcome measure should be reported

The use of several different outcome measures across studies makes it difficult for readers to be familiar with all the different measures. This increases the responsibility of authors to provide information on validity and reliability. Many of the studies in this review were large, including several hundred exposed pregnancies, thus limiting the risk of random error. In these studies, the most important psychometric property to report is validity, as invalid measures may introduce bias.

In psychology, construct validity is often considered the most important form of validity, as there are no objective criteria or “gold standards” to compare to [161]. Quite surprisingly, only three papers mentioned structural validity, one of which provided quantitative measures, and no papers explicitly mentioned hypothesis testing. Construct validity can be evaluated using statistical methods, and therefore numbers ought to be reported.

Criterion validity can also be evaluated using statistical methods. About a third of the papers provided a comment on criterion validity for at least one of their outcome measures, however only 15 reported a quantitative measure of criterion validity. Concurrent validity, the performance of the psychometric instrument or diagnosis against a gold standard, was reported for 28 papers. Specifically for diagnoses in registry-based studies, concurrent validity can both refer to the validity of the diagnostic criteria, and to the validity of the registration of the investigated diagnoses in the particular registry the data stems from. However, only the latter was mentioned in the papers identified in this review. Predictive validity is mainly relevant to psychometric instruments, and was only mentioned by 11 papers. Predictive validity is important for measures used in children, as children may grow into or out of difficulties [22].

Only one paper mentioned content validity [55], the degree to which the questions or tasks that make up a psychometric instrument, or the criteria that make up a diagnosis, are relevant and comprehensive measures of the domain of neurodevelopment that the outcome measure is used to investigate [19]. Content validity cannot be evaluated with the use of statistical methods. As such it is more subjective than the other forms of validity, which may make authors hesitant to comment on it. However, expert group evaluation of content validity has been done for diagnostic criteria and for some psychometric instruments [162,163], and could be reported. Another option is to make the questions or tasks of an outcome measure available to readers in a supplement (if copyright allows), so readers can assess content validity.

If the content or criterion validity is low or unknown, this should be reflected in the language used in the paper. For example, if a study uses a psychometric instrument to assess the presence of ADHD, and the instrument has not shown acceptable validity when tested against diagnostic interviews for ADHD, authors should write that they have assessed “symptoms of ADHD” and not “ADHD” as such.

In studies where a psychometric instrument is used in a different language or for another population than that in which it was developed, cross-cultural validity will tell us the extent to which the instrument measures the same as the original instrument. Without validation it cannot be assumed that the outcome measure is valid in a different population from the one for which it was developed [19]. Yet, cross-cultural validity alone will not be a sufficient measure of validity, as it does not provide any information on whether the original psychometric instrument measures what is intended.

We recognise that many journals have word limits for articles, making it difficult to include detailed information on reliability and validity. However, given the importance of this information it is suggested that this is prioritised. Today many journals allow online supplements, where psychometric properties can be described. For an example of how this could be done, see the online supplement to the paper by Avella-Garcia and colleagues [118]. Many papers identified in this review only referred to the manual of the outcome measure they use, which is often not accessible to the readers.

Finally, other methodological issues than use and validity of outcome measure can introduce between-study heterogeneity and should be considered. Some of these issues are the study limitations in observational studies according to GRADE [27], as assessed in S5–S7 Tables. Other examples include choice of appropriate comparator group to handle confounding by indication of medication use [156], analyses of direct and indirect medication effects by taking into account postnatal factors [164], and analysis of medication use by timing, dose and/or duration [165]. Interested readers are referred to a recent review on these methodological issues in medication safety studies with central nervous system outcomes [166].

## Conclusions

Studies have used several outcome measures including diagnoses and psychometric instruments completed by health care professionals, parents, or teachers to assess child neurodevelopment, yet few studies reported adequately on the reliability and validity of their outcomes. In order to establish neurodevelopmental safety of prenatal exposure to a medication, it is necessary to assess several domains of neurodevelopment until adolescence using age appropriate outcome measures. For medications where an animal model exists, this should inform which outcomes are assessed first. Authors should use reliable and valid outcome measures to assess neurodevelopment. We encourage reporting on the validity and reliability of the outcome measures used. In addition, results should be interpreted in light of the reliability and validity of the outcome measure that is used. Consensus is required on which outcome measure to use for each age group and data source in each domain of neurodevelopment. Until such consensus is in place, researchers should to a larger extent combine different data sources in one study, and authors of meta-analyses should be aware that in-depth knowledge of the various outcome measures is necessary when deciding which outcomes can be combined.

## Supporting information

S1 File Definitions of different types of reliability and validity. (PDF)

S2 File Search terms and search strategy, example for the MEDLINE database. (PDF)

S3 File PRISMA checklist. (PDF)

S1 Table Study characteristics, papers on antidepressants. (PDF)

S2 Table Study characteristics, papers on psychotropics except for antidepressants. (PDF)

S3 Table Study characteristics, papers on analgesics. (PDF)

S4 Table Reliability and validity of the outcome measures used in studies on neurodevelopmental safety of prenatal exposure to psychotropics and analgesics. (PDF)

S5 Table Risk of bias assessments, papers on antidepressants. (PDF)

S6 Table Risk of bias assessments, papers on psychotropics except for antidepressants. (PDF)

S7 Table Risk of bias assessments, papers on analgesics. (PDF)
