# Supplementary material for: Use and validity of child neurodevelopment outcome measures in studies on prenatal exposure to psychotropic and analgesic medications – A systematic review
Source: PLoS One. 2019 Jul 11;14(7):e0219778. doi: 10.1371/journal.pone.0219778 (PMC6622545; doi:10.1371/journal.pone.0219778)
Supplement: S1 File — (PDF) [file pone.0219778.s001.pdf]

S1 File. Definitions of different types of reliability and validity.

Reliability: The extent to which a measurement is free from measurement error. Thus low reliability will mainly introduce random error.

Internal consistency: In psychometric instruments made up of several questions or tasks, the internal consistency is the degree to which the various questions or tasks are interrelated. This can also be seen as the extent to which a child will have the same score using different sets of questions or tasks from the same instrument. Measured with Cronbach's alpha. A value between 0.70 and 0.90 is considered acceptable.

Test-retest reliability: The degree to which the same assessor, evaluating the same child with the same outcome measure after a period of time, will obtain the same score, given that the child has not changed. Measured with Cohen's kappa for dichotomous variables, weighted Cohen's kappa for ordinal variables, and intraclass correlation coefficient for continuous variables. Cohen's kappa values below 0.4 are considered poor, values between 0.4 and 0.75 are considered fair to good and values above 0.75 are considered excellent. An intraclass correlation coefficient of more than 0.7 is considered acceptable.

Inter-rater reliability: The degree to which different assessors, evaluating the same child with the same outcome measure, will obtain the same score. Measured the same way as test-retest reliability.

Validity: The extent to which the outcome measure truly measures what it is intended to measure. As such, invalid outcome measures may introduce bias.

Construct validity: The extent to which the outcome measure provides the expected scores based on what is already known about the domain of neurodevelopment that the outcome measure is used to examine. Construct validity can be divided in *structural validity*, *hypothesis testing* and *cross-cultural validity*.

Structural validity: The degree to which the number of dimensions in the outcome measure reflects the number of dimensions in the aspect of neurodevelopment investigated. For example communication consists of multiple dimensions, speech being only one of them, so if the instrument only measures one dimension, it will not be adequate. The structural validity is tested in a factor analysis. The comparative fit index should be close to 0.95 or higher, the root mean square error of approximation close to 0.06 or lower, and the standardized root mean square residual close to 0.08 or lower.

Hypothesis testing: Based on knowledge about the aspect of neurodevelopment the outcome measure is supposed to investigate, hypotheses about the performance of the outcome measure are set up and tested. For example an outcome measure that investigates non-verbal intelligence should have a high correlation to other measures of non-verbal intelligence, low correlation with measures of language ability, and should be able to discern between children with and without intellectual disability.

Cross-cultural validity: The extent to which a translated or culturally adapted version of the outcome measure performs as the original. Mainly relevant for psychometric instruments. After forwards and backwards translation and approval from the makers of the original instrument, cross-cultural validity can be assessed in the same way as structural validity by examining the model fit indices from a factor analysis.

Content validity: The degree to which the questions or tasks that make up an outcome measures are relevant and comprehensive measures of the domain of neurodevelopment that the outcome measure is used to investigate. Content validity cannot be evaluated with the use of statistical methods, but an expert group evaluation of the outcome measure is often recommended.

Criterion validity: The degree to which the outcome measure tests the same as the "gold standard" in the area. Criterion validity can be further divided in *concurrent validity* and *predictive validity*, depending on whether the outcome measure tests the same as the gold standard at the same point in time or predicts the gold standard in the future. Predictive validity is arguably mainly relevant for psychometric instruments. Both concurrent and predictive validity are measured as sensitivity and specificity, and/or area under the receiver operator curve, if the gold

standard is dichotomous. When evaluating validity in a clinical context, it is also recommended to include positive and negative predictive values. For criterion validity, values above 0.7 are considered acceptable.

Sensitivity: The proportion of actual positives (positives if using the gold standard) that are correctly identified as such, using the outcome measure.

Specificity: The proportion of actual negatives (negatives if using the gold standard) that are correctly identified as such, using the outcome measure.

Positive predictive value: The proportion of actual positives among those identified as positive when using the outcome measure.

Negative predictive value: The proportion of actual negatives among those identified as negative when using the outcome measure.

Source: de Vet, H. C. W., Terwee, C. B., Mokkink, L. B. & Knol, D. L. *Measurement in Medicine: A Practical Guide*. (Cambridge University Press, 2011).
