# Supplementary material for: Use and validity of child neurodevelopment outcome measures in studies on prenatal exposure to psychotropic and analgesic medications – A systematic review
Source: PLoS One. 2019 Jul 11;14(7):e0219778. doi: 10.1371/journal.pone.0219778 (PMC6622545; doi:10.1371/journal.pone.0219778)
Supplement: S2 File — (PDF) [file pone.0219778.s002.pdf]

S2 File. Search terms and search strategy, example for the MEDLINE database.

This example is from a search on January 17th 2018. The search for this review was updated on April 30th 2019, so final numbers differ.

**Prenatal exposure**	**Psychotropics and analgesics**	**Neurodevelopmental outcomes**
	**MeSH terms**	
Prenatal Exposure Delayed Effects<br>Maternal Exposure	Analgesics, Non-Narcotic<br>Analgesics, Opioid<br>Acetaminophen<br>Anti-Inflammatory Agents, Non-Steroidal<br>Serotonin 5-HT1 Receptor<br>Agonists<br>Tryptamines<br>Serotonin Uptake Inhibitors<br>Anti-Anxiety Agents<br>Antipsychotic Agents<br>Hypnotics and Sedatives	Mental Disorders<br>Mood Disorders<br>Anxiety Disorders<br>Sleep Wake Disorders<br>Temperament<br>Child Development<br>Neurodevelopmental Disorders<br>Developmental Disabilities<br>Cognition Disorders<br>Intelligence<br>Motor Skills Disorders<br>Autistic Disorder<br>Autism Spectrum Disorder<br>Child Development Disorders, Pervasive<br>Child Behavior Disorders<br>Attention Deficit Disorder with Hyperactivity
	**All fields**	
Prenatal exposure*<br>Maternal exposure*<br>Pregnancy exposure*<br>Fetal exposure*<br>Foetal exposure*	Analgesic*<br>NSAID<br>Non-steroidal anti-inflammatory<br>agent*<br>Ibuprofen<br>Paracetamol<br>Acetaminophen<br>Opioid*<br>Triptan*<br>Antidepress*<br>SSRI<br>Serotonin uptake inhibitor*<br>SNRI<br>Serotonin and noradrenaline reuptake	Emotional*<br>Depression<br>Anxiety<br>Sleep disorder*<br>Neurodevelopment*<br>Developmental disabilit*<br>Cognition*<br>IQ<br>Intelligence<br>Intellectual disabilit*<br>Psychomotor<br>PDD<br>Pervasive developmental<br>disorder*

	inhibitor*<br>TCA<br>Tricyclic antidepress*<br>Anxiolytic*<br>Sedative*<br>Hypnotic*<br>Antipsychotic*	Autism<br>ASD<br>Autism spectrum disorder*<br>Behavior disorder*<br>Behaviour disorder*<br>Hyperkinetic disorder*<br>ADHD<br>“Attention deficit disorder with hyperactivity”

#	Searches	Results
1	exp Prenatal Exposure Delayed Effects/	27602
2	exp Maternal Exposure/	8075
3	Prenatal exposure*.mp.	30443
4	Maternal exposure*.mp.	9664
5	Pregnancy exposure*.mp.	350
6	Foetal exposure*.mp.	145
7	Fetal exposure*.mp.	1988
8	1 or 2 or 3 or 4 or 5 or 6 or 7	38229
9	exp Analgesics, Non-Narcotic/	333616
10	exp Analgesics, Opioid/	111724
11	exp Acetaminophen/	17829
12	exp Anti-Inflammatory Agents, Non-Steroidal/	200681
13	exp Serotonin 5-HT1 Receptor Agonists/	3564
14	exp Tryptamines/	97148
15	exp Serotonin Uptake Inhibitors/	38019
16	exp Anti-Anxiety Agents/	67748
17	exp Antipsychotic Agents/	124295
18	exp "Hypnotics and Sedatives"/	124235
19	Analgesic*.mp.	140704
20	NSAID.mp.	12687
21	Non-steroidal anti-inflammatory agent*.mp.	1442
22	Ibuprofen.mp.	14316

23	Paracetamol.mp.	11256
24	Acetaminophen.mp.	23305
25	Opioid*.mp.	109027
26	Triptan*.mp.	2134
27	Antidepress*.mp.	89724
28	SSRI.mp.	6402
29	Serotonin uptake inhibitor*.mp.	20353
30	SNRI.mp.	841
31	(Serotonin and noradrenaline reuptake inhibitor*).mp. [mp=title, abstract, original title, name of substance word, subject heading word, keyword heading word, protocol supplementary concept word, rare disease supplementary concept word, unique identifier, synonyms]	920
32	TCA.mp.	10397
33	Tricyclic antidepress*.mp.	10281
34	Anxiolytic*.mp.	14448
35	Sedative*.mp.	41982
36	Hypnotic*.mp.	38210
37	Antipsychotic*.mp.	68712
38	9 or 10 or 11 or 12 or 13 or 14 or 15 or 16 or 17 or 18 or 19 or 20 or 21 or 22 or 23 or 24 or 25 or 26 or 27 or 28 or 29 or 30 or 31 or 32 or 33 or 34 or 35 or 36 or 37	970005
39	exp Mental Disorders/	1213750
40	exp Mood Disorders/	119446
41	exp Anxiety Disorders/	79004
42	exp Sleep Wake Disorders/	84569
43	exp Temperament/	5633
44	exp Child Development/	58195
45	exp Neurodevelopmental Disorders/	181796
46	exp Developmental Disabilities/	19678
47	exp Cognition Disorders/	88273
48	exp Intelligence/	106026
49	exp Motor Skills Disorders/	2862
50	exp Autistic Disorder/	22330

51	exp Autism Spectrum Disorder/	4516
52	exp Child Development Disorders, Pervasive/	34241
53	exp Child Behavior Disorders/	21855
54	exp Attention Deficit Disorder with Hyperactivity/	28428
55	Emotional*.mp.	147059
56	Depression.mp.	378625
57	Anxiety.mp.	215726
58	Sleep disorder*.mp.	20291
59	Developmental disabilit*.mp.	22589
60	Cognition*.mp.	192517
61	IQ.mp.	21047
62	Intelligence.mp.	77185
63	Intellectual disabilit*.mp.	62346
64	Psychomotor.mp.	89902
65	PDD.mp.	3493
66	Pervasive developmental disorder*.mp.	2259
67	Autism.mp.	39839
68	ASD.mp.	19198
69	Autism spectrum disorder*.mp.	19609
70	Behavior disorder*.mp.	32501
71	Behaviour disorder*.mp.	1120
72	Hyperkinetic disorder*.mp.	452
73	ADHD.mp.	23585
74	"Attention deficit disorder with hyperactivity".mp.	28544
75	Neurodevelopment*.mp.	28040
76	39 or 40 or 41 or 42 or 43 or 44 or 45 or 46 or 47 or 48 or 49 or 50 or 51 or 52 or 53 or 54 or 55 or 56 or 57 or 58 or 59 or 60 or 61 or 62 or 63 or 64 or 65 or 66 or 67 or 68 or 69 or 70 or 71 or 72 or 73 or 74 or 75	1969036
77	8 and 38 and 76	1576
