# Supplementary material for: Use and validity of child neurodevelopment outcome measures in studies on prenatal exposure to psychotropic and analgesic medications – A systematic review
Source: PLoS One. 2019 Jul 11;14(7):e0219778. doi: 10.1371/journal.pone.0219778 (PMC6622545; doi:10.1371/journal.pone.0219778)
Supplement: S1 Table — (PDF) [file pone.0219778.s004.pdf]

S1 Table. Study characteristics, papers on antidepressants.

Reference	Country/setting	Study design	Exposure/duration	Outcome/*Name of the scale*	Sample size/exposed	Effect estimate, Exposed/unexposed Adjusted effect estimates where available	Cohen's d*
**Assessment using psychometric instruments**							
**i. Assessment by health care professional**							
Infant (<2 years)							
Suri 2011 [99]	USA/Semel Institute for Neuroscience and Human Behavior	Cohort	Any AD/at least during 2nd and 3rd trimester versus unmedicated depression	Overall development/*BNBAS*	44/31	Mean (SD)<br>*Habituation*<br>6.04 (2.12)/4.50 (1.22)<br>*Orientation*<br>6.17 (2.13)/6.87 (0.85)<br>*Inanimate auditory*<br>4.93 (2.11)/6.10 (0.74)<br>*Motor*<br>5.89 (0.71)/6.20 (0.74)<br>*Defence*<br>7.19 (0.91)/7.00 (1.16)<br>*Range of state*<br>3.14 (0.96)/3.25 (1.04)<br>*Regulation of state*<br>4.46 (1.05)/4.29 (0.95)<br>*Autonomic stability*<br>7.48 (0.75)/7.67 (0.61)<br>*Reflexes*<br>3.13 (2.45)/2.46 (1.61)	0.81 Unk<br>-0.38 Unk<br>-0.64 Unk<br>-0.43 Unk<br>0.19 Unk<br>-0.11 Unk<br>0.17 Unk<br>-0.27 Unk<br>0.30 Unk
Mortensen 2003 [81]	Denmark/ National Health Service in North Jutland County	Cohort, registry based	Any AD/any duration	Motor skills/*Boel test*	805/50	OR (95% CI) of abnormal test result<br>5.9 (1.1 to 31.0)	0.98 High
Batton 2013 [44]	USA/ Hospital based	Cohort	SSRI/at the time of first pregnancy visit and throughout pregnancy	General development/*Bayley Infant Neurodevelopment Screener*	38/19	Percentage at high risk of neurodevelopmental delay<br>21%/42%	-0.46 High
Weikum 2013a	Canada/ Reproductive mental health program	Cohort	SSRI/any. Mean duration 237 days	Cognition/*BSID, unspecified edition*	72/30	Mean (SD)‡<br>*Motor*	

[101]						62.17 (3.54)/61.60 (6.89)<br>*Mental*<br>39.47 (3.38)/40.21 (6.37)	0.10<br><br>-0.14
Gustafsson 2018 [61]	USA/Emory Women's Mental Health Program and community cohort	Cohort	Any SRI/any	Overall development /*BSID-II*	80/not reported at follow-up	*Motor skills*<br>Mean difference<br>-9.87<br>*Mental development*<br>Mean<br>90/95	 <br>-0.69<br><br><br>-†
Oberlander 2004 [87]	Canada/ Reproductive mental health program	Cohort	SSRI/any. Most started AD medication in the first trimester and continued throughout pregnancy	Overall development /*BSID-II*	51/28	Mean (SD)<br>*Motor skills*<br>91.5 (9.6)/97.0 (9.1)<br>*Mental development*<br>100.7 (6.4)/99.4 (5.6)	 <br>-0.59<br><br>0.21
Reebye 2002 [92]	Canada/ Children's and Women's Health Centre of British Columbia	Cohort	SSRI/any. Mean duration 192 days.	Overall development /*BSID-II*	47/24	Mean (SD)<br>*Motor skills*<br>106 (5.4)/101 (7.9)<br>*Mental development*<br>98 (8.1)/96 (7.5)	 <br>0.74<br><br>0.26
Reebye 2012 [38]	Canada/ Reproductive Mental Health Program	Cohort	SSRI/at least from inclusion to follow-up	Overall development /*BSID-II*	45/22	Mean (SD)<br>*Motor skills*<br>91 (9.5)/97 (8.6)<br>*Mental development*<br>100 (6.1)/99 (5.5)	 <br>-0.66<br><br>0.17
Santucci 2014 [93]	USA/not stated	Cohort	SSRI, SNRI/ any duration	Overall development /*BSID-II*	38/26	Mean (SE)<br>*Motor skills*<br>99.3 (1.7)/105.2 (3.1)<br>*Mental development*<br>98.1 (2.9)/ 100.5 (4.8)<br>Percentage with score at or above 75%<br>*Behaviour rating scale, total score*<br>72.0%/81.8%	 <br>-0.64<br><br>-0.16<br><br><br>-0.22
Austin 2013 [43]	Australia/Hospital and TIS based	Cohort	Any AD/any duration	General development/*BSID-III*	58/35	Mean (SD)<br>*Gross motor*<br>9.2 (2.6)/9.1 (2.0)<br>*Fine motor*	 <br>0.04

						12.1 (2.4)/11.8 (2.0)	0.13
						Cognition	
						11.1 (2.1)/12.6 (2.6)	-0.65
						Receptive language	
						9.5 (2.5)/10.0 (2.1)	-0.21
						Expressive language	
						9.2 (2.7)/9.7 (2.3)	-0.20
Hanley 2013 [64]	Canada/clinic based	Cohort	SSRI from conception/ any duration	Overall development/*BSID-III*	83/31	Mean (SD)‡	
						Gross motor	
						8.3 (3.3)/ 9.5 (2.8)	-0.40
						Fine motor	
						11.3 (2.2)/ 11.2 (1.7)	0.05
						Cognition	
						11.5 (2.4)/10.9 (2.1)	0.27
						Receptive language	
						10.2 (2.2)/10.2 (2.3)	0.00
						Expressive language	
						9.9 (2.1)/9.8 (1.6)	0.06
						Socio-emotional	
						8.5 (2.2)/9.6 (2.5)	-0.46
						Adaptive	
						68.4 (12.4)/73.3 (10.3)	-0.44
Heikkinen 2002 [67]	Finland/Turku university hospital	Cohort	Citalopram/ from conception and through to lactation	Overall development/*Gesell Development scales*	21/11	Number with abnormal development (N)<br>0 (11)/0 (10)	undefined
Heikkinen 2003 [36]	Finland/Turku university hospital	Cohort	Fluoxetine/from conception for most participants and through to lactation	Overall development/*Gesell Development scales*	21/11	Number with abnormal development (N)<br>0 (11)/0 (10)	undefined
Johnson 2012 [73]	USA/Emory Women's Mental Health Program and community cohort	Cohort	Any AD/any (median 40 weeks)	*Motor skills* /*Infant Neurological International Battery*	261/183	Mean (SE)<br>68.58 (0.60)/70.12 (1.03)	-0.18
de Vries 2013 [53]	The Netherlands/ Hospital and clinic based	Cohort	SSRI, initiated before pregnancy/any duration	*Motor skills* / *Psychomotor assessment*	107/63	*General movements*<br>Number with abnormal score (N)<br>6 (63)/0 (44)	0.51 High

				according to Prechtl		*Motor optimality score*<br>Median (min/max)<br>26 (7/28)/28 (21/28)	-†
Preschool (2-5 years)							
Nulman 2002 [84]	Canada/Motherisk	Cohort	Fluoxetine and TCA/throughout pregnancy	Overall development/*BSID-II*	Fluoxetine 51/33	Mean (SD)<br>*Motor skills*<br>Fluoxetine<br>97.7 (11.0)/98.3 (9.7)	-0.06
					TCA 46/28	TCA<br>100.1 (12.5)/ 98.3 (9.7)	0.16
						Mental<br>Fluoxetine<br>104.4 (15.5)/104.1 (13.7)	0.02
						TCA<br>110.9 (18.0)/ 104.1 (13.7)	0.41
				Cognition/*Reynell developmental language scale*	Fluoxetine 72/38	*Verbal comprehension*<br>Fluoxetine<br>0.2 (1.3)/0.4 (1.0)	-0.17[Unk]
					TCA 89/45	TCA<br>1.1 (0.9)/0.4 (1.0)	0.74 [Unk]
						Expressive language<br>Fluoxetine<br>-0.3 (1.1)/-0.1 (1.2)	-0.17 [Unk]
						TCA<br>0.2 (1.0)/-0.1 (1.2)	0.28 [Unk]
				McCarthy scales of children's abilities	Fluoxetine 22/6	*Global cognitive index*<br>Fluoxetine<br>108.7 (19.9)/118.4 (9.1)	-0.76
					TCA 34/18	TCA<br>117.8 (10.4)/118.4 (9.1)	-0.06
Nulman 1997 [83]	Canada/TIS	Cohort	Fluoxetine and TCA/any duration	Overall development/*BSID-II*	Fluoxetine 139/55	Mean difference (95% CI)<br>Fluoxetine<br>2.1 (-5.0 to 9.2)	0.10
					TCA 164/80	TCA<br>2.4 (-4.5 to 9.4)	0.11
Casper 2003	USA/Women's Well-	Cohort	SSRI/any duration	Overall develop-	44/31	Mean (SD)	

Formatted: English (U.K.)

[49]	ness Clinic		compared to unmedicated depression	ment/*BSID-II*		*Motor skills*<br>90.0 (11.4)/98.2 (9.1)<br>*Mental development*<br>91.0 (13.3)/94.3 (7.5)<br>*Behaviour*<br>76.0 (24.6)/89.5 (15.4)	 -0.76<br><br>-0.28<br><br>-0.60
Batton 2013 [44]	USA/ Hospital based	Cohort	SSRI/at the time of first pregnancy visit and throughout pregnancy	General development/*BSID-III*	30/15	Mean (SD)<br>*Motor skills*<br>79 (21)/75 (20)<br>*Cognition*<br>94 (15)/91 (10)	 <br>0.20<br><br>0.24
Galbally 2011 [57]	Australia/Hospital based	Cohort	Any AD/any duration	Overall development/*BSID-III*	41/19	Mean (SD)<br>*Gross motor*<br>12.89 (2.75)/14.18 (3.13)<br>*Fine motor*<br>12.84 (2.99)/14.18 (2.70)<br>*Cognition*<br>13.16 (2.97)/13.50 (2.18)<br>*Receptive language*<br>11.42 (1.46)/12.00 (2.61)<br>*Expressive language*<br>10.53 (1.43)/11.24 (2.30)<br>*Socio-emotional*<br>10.61 (2.57)/11.06 (2.67)	 <br>-0.44<br><br>-0.47<br><br>-0.13<br><br>-0.27<br><br>-0.36<br><br>-0.17
Hurault-Delarue 2016 [69]	France/ EFEMERIS database	Cohort, registry based	Any AD/any duration in 2nd or 3rd trimester	Overall development/*Compulsory medical exam*	32 476/173	Percentage with abnormal development<br>*Motor*<br>1.7%/0.4%<br>*Mental*<br>2.5%/1.0%	 <br>0.08 [High]<br><br>0.08 [High]
Schechter 2017 [94]	USA/ Emory Women's Mental Health Program and community cohort	Cohort	SSRI/any duration	Cognition/*DAS*	162/not reported	Pearson correlation<br>0.06	-†
Johnson 2016 [74]	USA/Emory Women's Mental Health Program and community	Cohort	SSRI/any duration	Cognition/*DAS-II*	178/102	Pearson correlation<br>*Cognitive functioning*<br>0.016	 <br>-†

	cohort			*Test of early language development, 3rd edition*		Mean difference (95% CI)<br>*Expressive language*<br>-0.105 (-0.190 to -0.019)	-0.37
Galbally 2015 [58]	Australia/Victorian Psychotropic Registry study	Cohort	Any AD/any duration	*Motor skills* /*Movement ABC*<br><br>Cognition/*WPPSI-III*	41/20	Mean (SD)<br>*Movement ABC*<br>80.40 (13.11)/83.43 (12.12)<br>*WPPSI-III*<br>115.40 (13.96)/115.76 (8.29)	 <br>-0.24<br><br>-0.03
School child (6-12 years)							
Nulman 1997 [83]	Canada/TIS	Cohort	Fluoxetine, TCA/any duration	Cognition/*Reynell developmental language scale*<br><br><br><br><br><br><br><br><br><br>*McCarthy scales of children's abilities*	Fluoxetine 139/55<br><br>TCA 164/80	Mean difference (95% CI)<br>*Verbal comprehension*<br>Fluoxetine<br>0.3 (-0.1 to 0.6)<br>TCA<br>0.3 (-0.1 to 0.5)<br>*Expressive language*<br>Fluoxetine<br>-0.1 (-0.4 to 0.3)<br>TCA<br>0.0 (-0.3 to 0.3)<br>*Global cognitive index*<br>Fluoxetine<br>4.7 (-4.0 to 13.4)<br>TCA<br>2.7 (-2.3 to 7.6)	 <br><br>0.29 Unk<br><br>0.31 Unk<br><br><br>-0.10 Unk<br><br>0.00 Unk<br><br><br>0.19<br><br>0.17
El Marroun 2017 [55]	The Netherlands/ Generation R	Cohort	SSRI/any duration	Cognition, *SON-R, shortened*<br><br>Overall development/*NEPSY-II*	4 661/57<br><br>1 044/49	Mean difference (95%CI)<br>*Non-verbal IQ*<br>0.87 (-2.87 to 4.61)<br>*Sensory motor*<br>0.17 (-0.09 to 0.44)<br>*Visuospatial*<br>0.02 (-0.24 to 0.27)<br>*Memory and learning*<br>0.02 (-0.24 to 0.28)<br>*Language*<br>-0.45 (-0.70 to -0.19)<br>*Attention and executive*	 <br>0.06<br><br>0.18<br><br>0.02<br><br>0.02<br><br>-0.51

						-0.05 (-0.33 to 0.23)	-0.05
Hermansen 2016 [68]	Norway/substudy in MoBa	Cohort	SSRI/any duration compared to unmedicated depression	Cognition/*WPPSI-R, shortened*	70/28	Mean (SD)<br>*Reasoning*<br>10.92 (1.83)/11.55 (2.02)<br>*Similarities*<br>11.36 (2.38)/11.48 (2.91)<br>*Vocabulary*<br>12.19 (2.40)/12.62 (2.71)<br>*Block design*<br>9.14 (3.87)/9.79 (3.40)	 <br>-0.32<br><br>-0.04<br><br>-0.17<br><br>-0.18
				NEPSY-II, shortened		*Visual attention*<br>4.52 (0.58)/4.44 (0.55)<br>*Statue*<br>3.68 (1.02)/3.69 (1.22)	 0.14<br><br>-0.01
Nulman 2012 [85]	Canada/Motherisk	Cohort	SSRI, venlafaxine/any, median 30 weeks, versus unmedicated depression	Cognition/*WPPSI-III*	116/62	Mean (SD)<br>*Full scale IQ*<br>SSRI<br>105 (13)/108 (14)<br>Venlafaxine<br>105 (14)/108 (14)	 <br><br>-0.22<br><br>-0.21
Nulman 2015 [86]	Canada/Motherisk	Cohort	SSRI /any, median 36 weeks, versus unexposed sibling	Cognition/*WPPSI-III*	90/45	Mean (SD)<br>*Full scale IQ*<br>103 (12.9)/106 (12.3)	 <br>-0.24
Adolescent (13-18 years)							
Mattson 2002 [79]	USA/ California TIS and Clinical Research Program	Cohort	Any AD /at least one 1st trimester exposure	Cognition/*WISC-III*	165/83	Mean<br>105.8/104.9	-†
**ii. Assessment by parents**							
Infant (<2 years)							
Reebye 2002 [92]	Canada/ Children's and Women's Health Centre of British Columbia	Cohort	SSRI/any. Mean duration 192 days.	Behaviour/*Early infant temperament questionnaire*	47/24	Percentage with difficult [easy] temper<br>0% [8%]/4% [17%]	 -0.30 High<br>[-0.14] High

Netsi 2015 [82]	UK/Pilot RCT on cognitive therapy	Cohort	SSRI/any duration	Behaviour/*Infant characteristic questionnaire*<br>Other/*Brief infant sleep questionnaire*	25/not reported	*Fussy-difficult*<br>Not significant, numbers not reported<br>*Sleep*<br>Not significant, numbers not reported	-†
Nulman 1997 [83]	Canada/TIS	Cohort	Fluoxetine and TCA/any duration	Behaviour/*Toddler temperament scale*	Not reported	Not significant, numbers not reported	-†
Nulman 2002 [84]	Canada/Motherisk	Cohort	Fluoxetine and TCA/throughout pregnancy	Behaviour/*Toddler temperament scale*	Not reported	Not significant, numbers not reported	-†
Preschool (2-5 years)							
Handal 2016a [62]	Norway/MoBa	Cohort	SSRI/one period or two to three periods with or without symptoms of depression/anxiety	*Motor skills /ASQ*	45 085/381	OR (95% CI)<br>*Gross motor*<br>SSRI short term, no symptoms<br>0.98 (0.90 to 1.06)<br>SSRI long term, no symptoms<br>1.52 (0.94 to 2.46)<br>SSRI short term, with symptoms<br>1.10 (0.71 to 1.72)<br>SSRI long term, with symptoms<br>1.76 (1.04 to 2.96)<br>*Fine motor*<br>SSRI short term, no symptoms<br>0.73 (0.50 to 1.08)<br>SSRI long term, no symptoms<br>1.48 (1.06 to 2.07)<br>SSRI short term, with symptoms<br>1.37 (0.92 to 2.05)<br>SSRI long term, with symptoms<br>1.47 (0.94 to 2.29)	-0.01 High<br>0.23 High<br>0.05 High<br>0.31 High<br>-0.17 High<br>0.22 High<br>0.17 High<br>0.21 High
El Marroun 2017 [55]	The Netherlands/ Generation R	Cohort	SSRI/any duration	Behaviour/*BRIEF*	3 819/44	Mean difference (95%CI)<br>3.43 (-1.29 to 8.16)	0.22 High
Galbally 2015 [58]	Australia/Victorian Psychotropic Registry study	Cohort	Any AD/any duration	Behaviour/*CBCL*	41/20	Mean (SD)<br>*Externalising*<br>9.30 (6.42)/9.25 (7.73)	0.01 High

				BRIEF		*Internalising*<br>6.05 (4.86)/6.45 (5.47)<br>*Global executive composite*<br>88.15 (16.20)/84.95 (15.88)	 -0.08 [High]<br><br>0.20 [High]
Brandlistuen 2015 [46]	Norway/MoBa	Cohort, sibling study	Any AD/any duration	Behaviour/*CBCL*	121 sibling pairs	Beta (95% CI)<br>*Externalising*<br>-0.08 (-0.44 to 0.27)<br>*Internalising*<br>0.34 (-0.01 to 0.68)	 <br>-0.06 [High]<br><br>0.25 [High]
Hanley 2015 [65]	Canada/clinic based	Cohort	SSRI from conception/mean duration spanned most or almost all of the pregnancy	Behaviour/*CBCL*	110/44	Mean (SD)<br>*Externalising*<br>45.73 (12.35)/46.67 (8.39)<br>*Internalising*<br>50.25 (12.60)/45.70 (8.20)<br>*Anxious*<br>2.45 (2.64)/1.23 (1.59)<br>*Attention problems*<br>1.68 (1.47)/1.55 (1.56)	 <br>-0.09 [High]<br><br>0.45 [High]<br><br>0.59 [High]<br><br>0.09 [High]
Johnson 2016 [74]	USA/Emory Women's Mental Health Program and community cohort	Cohort	SSRI/any duration	Behaviour/*CBCL*	178/102	OR (95% CI)<br>*PDD scale*<br>1.05 (1.01 to 1.08)	 <br>0.07 [High]
Misri 2006 [80]	Canada/ Reproductive mental health program	Cohort	SSRI/any.<br>Mean duration 191 days	Behaviour and emotionality/parents *CBCL*	27/13	Mean (SD)<br>*Internalising*<br>53.31 (10.14)/54.00 (8.91)<br>*Somatic complaints*<br>54.62 (5.78)/56.71 (7.88)<br>*Withdrawn*<br>54.92 (6.22)/58.07 (5.73)<br>*Emotionally reactive*<br>55.08 (7.21)/55.00 (5.99)<br>*Anxious/depressed*<br>56.46 (7.46)/53.00 (2.96)	 <br>-0.07 [High]<br><br>-0.30 [High]<br><br>-0.53 [High]<br><br>0.01 [High]<br><br>0.62 [High]
Nulman 2002 [84]	Canada/Motherisk	Cohort	Fluoxetine and TCA/throughout pregnancy	Behaviour/*CBCL*	Not reported	Not significant, numbers not reported	-†

Oberlander 2007 [88]	Canada/ Reproductive mental health program	Cohort	SSRI/any. Mean duration 181.5 days	Behaviour/parents *CBCL*	36/22	Mean (SD)	
						Externalising	
						49.8 (10.7)/48.4 (10.1)	0.13 [High]
						ADHD symptoms	
						52.3 (3.4)/53.1 (5.0)	-0.20 [High]
						Oppositional defiant problems	
						56.1 (8.1)/50.8 (15.6)	0.46 [High]
						Attention problems	
						51.5 (1.8)/53.8 (7.7)	-0.46 [High]
						Aggressive behaviour	
						55.6 (8.1)/53.7 (6.3)	0.25 [High]
Oberlander 2010 [89]	Canada/ Reproductive mental health program	Cohort	SSRI or SNRI from conception/any duration	Behaviour/*CBCL*	75/33	Mean (SD)	
						Externalising	
						45.6 (11.2)/45.9 (7.4)	-0.03 [High]
						Internalising	
						51.0 (9.8)/45.7 (8.7)	0.58 [High]
						Sleep problems	
						52.9 (4.4)/54.0 (4.4)	-0.25 [High]
Lupattelli 2018 [76]	Norway/MoBa	Cohort	SSRI/any in midpregnancy (week 17-28) or late pregnancy (week 29 and later)	Behaviour/*CBCL*		Beta (95% CI)	
						Externalising	
					4 101/<br>102	Mid	
						-0.20 (-0.78 to 0.38)	-0.07 [High]
					4105/88	Late	
						0.21 (-0.40 to 0.82)	0.07 [High]
						Internalising	
						Mid	
						-0.14 (-0.76 to 0.49)	-0.04 [High]
						Late	
						0.39 (-0.25 to 1.02)	0.13 [High]
				EAS (activity, sociability, shyness)		*Activity*	
						Mid	
						-0.19 (-0.56 to 0.18)	-0.10 [High]
						Late	
						0.13 (-0.31 to 0.57)	0.06 [High]
						Shyness	
						Mid	
						0.30 (-0.29 to 0.90)	0.10 [High]
						Late	

						-0.08 (-0.79 to 0.63)<br>*Sociability*<br>Mid<br>-0.47 (-1.04 to 0.10)<br>Late<br>0.25 (-0.42 to 0.92)	-0.02 [High]<br><br><br>-0.16 [High]<br><br>0.08 [High]
				Emotionality/*EAS (emotionality)*		*Emotionality*<br>Mid<br>-0.28 (-0.93 to 0.37)<br>Late<br>0.12 (-0.60 to 0.85)	 <br>-0.08 [High]<br><br>0.03 [High]
Handal 2016b [63]	Norway/MoBa	Cohort	SSRI with or without folic acid/1, 2-3 or 4-8 intervals in pregnancy (each interval is 3 weeks long).	Language/ *Intelligibility/Complexity of 3-year-old Children's Utterances*	43 322/not reported	RR (95% CI)<br>*Fairly complete sentences vs. long complicated sentences*<br>SSRI + folic acid, long<br>4.5 (2.5 to 8.0)<br>*Language delay vs. long complicated sentences*<br>SSRI + folic acid, long<br>5.7 (2.5 to 13.0)	-†
Skurveit 2014 [96]	Norway/MoBa	Cohort	SSRI/one period or two to three periods of pregnancy	Language/*Intelligibility/Complexity of 3-year-old Children's Utterances*	51 587/225, one period<br>51 523/161, two/three periods	RR (95% CI)<br>*Fairly complete vs. long complicated sentences*<br>SSRI, one period<br>1.21 (0.85 to 1.72)<br>SSRI, two/three periods<br>2.28 (1.54 to 3.38)<br>*Language delay vs. long complicated sentences*<br>SSRI, one period<br>0.86 (0.42 to 1.76)<br>SSRI, two/three periods<br>2.30 (1.21 to 4.37)	 <br><br>0.11 [High]<br><br>0.45 [High]<br><br><br>-0.08 [High]<br><br>0.46 [High]
Pedersen 2013 [90]	Denmark/substudy in the DNBC	Cohort	Any AD/any duration versus unmedicated depression	Behaviour/*SDQ*	225/127	RD (95% CI)<br>*Total difficulties*<br>-0.7 (-1.8 to 0.4)<br>*Prosocial*	 <br>-0.17 [High]

						0.1 (-0.4 to 0.5)	0.06
School child (6-12 years)							
Hutchison 2019 [70]	Canada/ Reproductive mental health program	Cohort	SSRI/any duration versus unmedicated mood disorder	Behaviour/*BRIEF*	139/51	Pearson correlation<br>-0.17	-†
Hermansen 2016 [68]	Norway/substudy in MoBa	Cohort	SSRI/any	Behaviour/*CBCL*	70/28	Mean (SD)<br>*Externalising*	
						48.00 (10.34)/49.03 (13.36)	-0.08 [High]
						Internalising	
						51.41 (10.42)/48.46 (10.42)	0.28 [High]
Nulman 1997 [83]	Canada/TIS	Cohort	Fluoxetine and TCA/any	Behaviour/*CBCL*	Not reported	Not significant, numbers not reported	-†
Nulman 2012 [85]	Canada/Motherisk	Cohort	SSRI and venlafax-ine/any, median 30 weeks	Behaviour/*CBCL*	116/62	Percentage of children with problems<br>*Externalising*	
						SSRI<br>8%/9%	-0.04 [High]
						Venlafaxine<br>8%/9%	-0.04 [High]
						Internalising	
						SSRI<br>7%/4%	0.13 [High]
						Venlafaxine<br>10%/4%	0.24 [High]
				CPRS		*Total problems*	
						SSRI<br>17%/2%	0.57 [High]
						Venlafaxine<br>8%/2%	0.30 [High]
						DSM-IV total symptoms	
						SSRI<br>12%/13%	-0.05 [High]
						Venlafaxine<br>12%/13%	-0.05 [High]
Nulman 2015	Canada/Motherisk	Cohort	SSRI /any, median 36 weeks	Behaviour/*CBCL*	90/45	Percentage of children with problems<br>*Externalising*	

[86]						11.1%/ 11.1%	0.00 [High]
						Internalising	
						11.1%/6.7%	0.16 [High]
				CPRS-R		Not significant, numbers not reported	-†
El Marroun 2014 [54]	The Netherlands/ Generation R	Cohort	SSRI/any	Behaviour/parents *CBCL*	5 600/ 69	CBCL: OR (95% CI)	
						Pervasive developmental problems	
						2.58 (1.46 to 4.54)	0.52 [High]
					4 042/ 50	*Affective problems*	
						1.37 (0.87 to 2.16)	0.17 [High]
						SRS: Beta (95% CI)	
				Social responsiveness scale (SRS)		*Autistic traits*	
						0.17 (0.10 to 0.24)	0.68 [High]
Hanley 2015 [65]	Canada/ clinic based	Cohort	SSRI from conception/mean duration spanned most or almost all of the pregnancy	Behaviour/*HBQ-P*	110/44	Mean (SD)	
						Externalising	
						0.26 (0.20)/0.29 (0.22)	-0.14 [High]
						Internalising	
						0.39 (0.28)/0.29 (0.21)	0.42 [High]
						Overanxious	
						0.46 (0.32)/0.38 (0.26)	0.28 [High]
						Inattention	
						0.53 (0.41)/0.61 (0.40)	-0.20 [High]
Weikum 2013b [102]	Canada/ Reproductive mental health program	Cohort	SSRI from conception/any duration	Behaviour/*HBQ-P*	64/26	Mean (SD)	
						Externalising	
						0.23 (0.17)/0.29 (0.22)	-0.30 [High]
						Internalising	
						0.33 (0.22)/0.33 (0.25)	0.00 [High]
						ADHD symptoms	
						0.47 (0.37)/0.67 (0.39)	-0.52 [High]
Grzeskowiak 2016 [60]	Denmark/DNBC	Cohort	Any AD/any duration compared to untreated depression	Behaviour/parents *SDQ*	441/210	RR (95% CI)	
						Total difficulties	
						0.84 (0.31 to 2.31)	-0.10 [High]
						Pro-social	
						0.19 (0.05 to 0.77)	-0.93 [High]
						Impact score	
						0.76 (0.40 to 1.46)	-0.17 [High]

iii. Assessment by teachers/others

Preschool (2-5 years)							
Johnson 2016 [74]	USA/Emory Women's Mental Health Program and community cohort	Cohort	SSRI/any duration	Behaviour/other caregiver (teacher, baby sitter, grandmother, father)<br>*CBCL*	140/not reported	OR (95% CI)<br>*PDD scale*<br>1.01 (0.98 to 1.05)	-†
Misri 2006 [80]	Canada/ Reproductive mental health program	Cohort	SSRI/any.<br>Mean duration 191 days	Behaviour and emotionality/teachers<br>*CBCL*	27/13	Mean (SD)<br>*Internalising*<br>53.73 (7.77)/48.69 (10.70)<br>*Somatic complaints*<br>52.91 (4.30)/50.92 (2.25)<br>*Withdrawn*<br>54.82 (4.67)/53.08 (4.35)<br>*Emotionally reactive*<br>54.64 (7.53)/55.15 (8.19)<br>*Anxious/depressed*<br>56.64 (7.31)/54.69 (7.10)	0.54 [High]<br>0.59 [High]<br>0.39 [High]<br>-0.06 [High]<br>0.27 [High]
Oberlander 2007 [88]	Canada/ Reproductive mental health program	Cohort	SSRI/any. Mean duration 181.5 days	Behaviour/teachers<br>*CBCL*	36/22	Mean (SD)<br>*Externalising*<br>49.6 (7.8)/48.2 (10.7)<br>*ADHD symptoms*<br>52.9 (4.1)/53.1 (4.7)<br>*Oppositional defiant problems*<br>54.0 (5.8)/54.5 (7.3)<br>*Attention problems*<br>52.8 (3.9)/52.3 (4.2)<br>*Aggressive behaviour*<br>53.2 (4.9)/54.2 (6.9)	0.16 [High]<br>-0.05 [High]<br>-0.08 [High]<br>0.12 [High]<br>-0.18 [High]
Assessment using medical diagnosis							
Boukhris 2017 [34]	Canada/Québec Pregnacy/Children Cohort	Cohort, registry based	SSRI, SNRI, TCA, combined therapy/any duration in 2nd or 3rd trimester	ADHD	143 466/ 1561<br>142 350/ 445	HR (95% CI)<br>SSRI<br>1.2 (0.9 to 1.6)<br>SNRI<br>1.4 (0.8 to 2.5)<br>Tricyclic AD<br>1.8 (1.0 to 3.1)	0.10 [High]<br>0.19 [High]<br>0.34 [High]

					142 132/<br>227<br><br>142 067/<br>162	Combined therapy<br>1.5 (0.8 to 2.8)	 0.23 [High]
Figueroa 2010<br>[56]	USA/MarketScan<br>claims data	Cohort, reg-<br>istry based	SSRI /any duration	ADHD	37 540/9<br>26	OR (95% CI)<br>SSRI<br>0.91 (0.51 to 1.60)	 <br>-0.05 [High]
Laugesen 2013<br>[37]	Denmark/National<br>registries	Cohort, reg-<br>istry based	SSRI, SNRI, TCA,<br>combined therapy/any	ADHD	Sibling<br>analysis<br>867<br><br><br>874 491/<br>11 721<br><br>863 533/<br>763<br><br>863 486/<br>716<br><br>863 974/<br>1 204	HR (95% CI)<br>Any AD, sibling analysis<br>0.7 (0.4 to 1.4)<br>Standard regression<br>SSRI<br>1.2 (1.0 to 1.5)<br>SNRI<br>1.0 (0.4 to 2.5)<br>TCA<br>1.1 (0.6 to 2.0)<br>Combined therapy<br>0.8 (0.4 to 1.7)	 <br>-0.20 [High]<br><br><br>0.10 [High]<br><br>0.00 [High]<br><br>0.05 [High]<br><br>-0.12 [High]
Man 2017 [78]	China/Hong Kong<br>territory wide registry	Cohort, reg-<br>istry based	SSRI /any duration	ADHD	Sibling<br>analysis<br>53 616<br><br><br>189 427/<br>425	HR (95% CI)<br>Any AD, sibling analysis<br>0.54 (0.17 to 1.74)<br>Standard regression<br>SSRI<br>1.11 (0.77 to 1.60)	 <br>-†<br><br><br>0.06 [High]
Castro 2016<br>[50]	USA/Electronic health<br>records, Massachusetts	Case-control,<br>registry<br>based	Any AD/any duration	ASD<br><br><br>ADHD	4 650/10<br>5<br><br><br>5 498/88	OR (95% CI)<br>*ASD*<br>0.90 (0.50 to 1.54)<br>*ADHD*<br>0.97 (0.53 to 1.69)	 <br>-0.06 [High]<br><br>-0.02 [High]

Clements 2015 [51]	USA/Electronic health records, Massachusetts	Case-control, registry based	Any AD/any duration	ASD<br><br><br>ADHD	5 399/120<br><br><br><br>7 874/131	OR (95% CI)<br>*ASD*<br>1.10 (0.70 to 1.70)<br>*ADHD*<br>1.81 (1.22 to 2.70)	 <br>0.05 High<br><br>0.33 High
Sujan 2017 [98]	Sweden/national registries	Cohort, registry based, sibling design	SSRI/any 1st trimester exposure	ASD<br><br>ADHD	24 969/9063	HR (95% CI)<br>0.81 (0.58 to 1.14)<br>*ADHD*<br>0.94 (0.73 to 1.22)	 -0.12 High<br><br>-0.04 High
Wibroe 2017 [103]	Denmark/national registries	Cohort, registry based	Any AD (the study reports N06 of which 99.5% is N06A)/at least two dispensations in pregnancy	PDD<br><br>ADHD	861 382/7 206	Percentage with diagnosis<br>*ASD*<br>0.2%/0.1%<br>*ADHD*<br>0.3%/0.2%	 <br>0.02 High<br><br>0.01 High
Malm 2016 [77]	Finland/National registries	Cohort, registry based	SSRI /any duration versus unmedicated psychiatric disorder	ASD, ADHD, depression, anxiety	25 380/15 729	HR (95% CI)<br>*ASD*<br>0.88 (0.65 to 1.20)<br>*Depression*<br>1.78 (1.12 to 2.82)<br>*Anxiety*<br>1.30 (0.84 to 2.01)<br>*ADHD*<br>0.98 (0.77 to 1.24)	 <br>-0.07 High<br><br>0.32 High<br><br>0.15 High<br><br>-0.01 High
Liu 2017 [75]	Denmark/National registries	Cohort, registry based	Any AD/any during 2 years before pregnancy and during pregnancy (continuers) versus any during period from 2 years to 1 month before pregnancy, but not in pregnancy (discontinuers).	Psychiatric disorders/diagnosis	47 639/17 560	HR (95% CI)<br>*ASD*<br>1.23 (1.01 to 1.51)<br>*Mood disorder*<br>2.76 (1.59 to 4.78)<br>*Neurotic, stress-related and somatoform disorder*<br>1.62 (1.36 to 1.94)<br>*Behavioural and emotional disorder*<br>1.13 (1.01 to 1.27)<br>*Mental retardation*<br>1.21 (0.78 to 1.90)	 <br>0.12 High<br><br>0.56 High<br><br>0.27 High<br><br>0.07 High<br><br>0.11 High

Boukhris 2016 [45]	Canada/Québec Pregnacy/Children Cohort	Cohort, registry based	SSRI, SNRI, TCA, combined therapy/any duration	ASD	144 507/ 1 583<br>143 371/ 447<br>143 153/ 229<br>143 091/ 167	HR (95% CI)<br>SSRI<br>2.17 (1.20 to 3.39)<br>SNRI<br>1.04 (0.20 to 5.46)<br>Tricyclic AD<br>1.03 (0.23 to 4.61)<br>Combined therapy<br>4.39 (1.44 to 13.32)	0.43 High<br>0.02 High<br>0.02 High<br>0.83 High
Brown 2017 [48]	Canada/ Ontario registries on women eligible for drug benefits	Cohort, registry based	SSRI, SNRI/2 or more consecutive filled prescriptions	ASD	620 sibling pairs<br>35 236/2 167<br>33 894/8 25	HR (95% CI)<br>AD sibling analyses<br>1.51 (0.63 to 3.60)<br>SSRI<br>1.40 (0.80 to 2.46)<br>SNRI<br>1.53 (0.58 to 4.03)	0.23 High<br>0.19 High<br>0.24 High
Croen 2011 [52]	USA/Kaiser Permanente Medical Care Program in Northern California	Case-control, registry based	SSRI or TCA/dual-action antidepressants (DAA)/any duration	ASD	SSRI: 1 784/49<br>TCA/ DAA: 1 756/21	OR (95% CI)<br>SSRI only<br>2.6 (1.3 to 5.4)<br>TCA/DAA<br>1.6 (0.5 to 4.5)	0.53 High<br>0.26 High
Gidaya 2014 [59]	Denmark/National health registry	Case-control, registry based	SSRI/any duration	ASD	57 365/4 41	OR (95% CI)<br>1.8 (1.4 to 2.3)	0.32 High
Hviid 2013 [71]	Denmark/National registries	Cohort, registry based	SSRI/any during 2 years before pregnancy and during pregnancy (continuers), or any during pregnancy only (initiators)	ASD	626 875/ not reported	HR (95% CI)<br>Continuers<br>1.08 (0.74 to 1.58)<br>Initiators<br>1.40 (0.92 to 2.13)	-†
Janecka 2018	Israel/Meuhedet health maintenance organisa-	Case-control, registry	Clomipramine/any	ASD	1 437/33	HR (95% CI)<br>2.40 (0.31 to 18.63)	0.49 High

[72]	tion	based					
Rai 2017 [91]	Sweden/<br>Stockholm youth co-<br>hort	Cohort, reg-<br>istry based	Any AD/any duration	ASD	15 667/3<br>342	OR (95% CI)<br>Illness comparator<br>1.45 (1.13 to 1.85)<br>Sibling analysis<br>1.36 (0.84 to 2.20)	 <br>0.20 High<br><br>-†
Sorensen 2013<br>[97]	Denmark/national<br>registries	Cohort, reg-<br>istry based	SSRI, SNRI,<br>TCA/any	Autism	SSRI,<br>sibling<br>6 117,<br>standard<br>regres-<br>sion<br>SNRI,<br>647 455/<br>673<br>TCA,<br>647 424/<br>642<br>654 228/<br>7 506	HR (95% CI)<br>*ASD*<br>SSRI, sibling design<br>0.9 (0.4 to 2.0)<br>Standard regression<br>SNRI<br>1.7 (0.8 to 3.5)<br>TCA<br>1.5 (0.8 to 2.9)<br>*Childhood autism*<br>Standard regression<br>SSRI<br>1.4 (0.9 to 2.0)	 <br><br>-0.06 High<br><br><br>0.30 High<br><br>0.23 High<br><br><br><br>0.19 High
Viktorin 2017b<br>[41]	Sweden/national regis-<br>tries	Cohort, reg-<br>istry based	Sertraline, citalopram,<br>fluoxetine, venlafax-<br>ine, paroxetine/at<br>least two dispensa-<br>tions versus unmedi-<br>cated women with a<br>history of mental<br>disorders	ASD	 17 101/6<br>72<br><br>17 068/6<br>39<br><br>16 756/3<br>27<br><br>16 624/1<br>95<br><br>16 537/1<br>08	RR (95% CI)<br>Sertraline<br>1.17 (0.71 to 1.95)<br>Citalopram<br>1.47 (0.92 to 2.35)<br>Fluoxetine<br>1.08 (0.53 to 2.21)<br>Venlafaxine<br>0.88 (0.32 to 2.38)<br>Paroxetine<br>1.21 (0.38 to 3.80)	 <br>0.09 High<br><br>0.21 High<br><br>0.04 High<br><br>-0.07 High<br><br>0.11 High

Harrington 2014 [66]	USA/Childhood Autism Risks from Genetics and the Environment Study	Case-control	SSRI/any duration	ASD<br><br>Developmental delay	812/38<br><br>474/18	OR (95% CI)<br>*ASD*<br>1.86 (0.76 to 4.58)<br>*DD*<br>1.44 (0.44 to 4.75)	 <br>0.34 High<br><br>0.20 High
Brown 2016 [47]	Finland/National registries	Cohort, registry based	SSRI/any duration	Motor skills disorder, speech language disorder, scholastic skills disorder	25 133/not reported	HR (95% CI)<br>*Motor skills*<br>1.18 (0.81 to 1.72)<br>*Speech/language*<br>1.20 (0.97 to 1.49)<br>*Scholastic skills*<br>1.00 (0.63 to 1.59)	 <br>0.09 High<br><br>0.10 High<br><br>0.00 High
Simon 2002 [95]	USA/Group Health Cooperative	Cohort, registry based	SSRI and TCA/any duration	Developmental delay	SSRI 370/185<br>TCA 418/209	OR (95% CI)<br>*Motor*<br>SSRI<br>3.07 (0.61 to 15.40)<br>TCA<br>1.00 (0.14 to 7.17)<br>*Speech*<br>SSRI<br>1.00 (0.14 to 7.18)<br>TCA<br>1.00 (0.14 to 7.17)	 <br><br>0.62 High<br><br>0.00 High<br><br><br>0.00 High<br><br>0.00 High
Viktorin 2017a [100]	Sweden/national registries	Cohort, registry based	SSRI/at least two dispensations	Intellectual disability	175 824/ 3 178	RR (95% CI)<br>1.48 (0.98 to 2.23)	 0.22 High

Reference numbers in brackets refer to the reference list in the article.

*Effect sizes (Cohen's d) were calculated using the metaeff package [1] for Stata [2]. [High] indicates that higher/more positive values mean a higher risk among the exposed, [Unk] means that there is not enough information in the paper or from psychometric literature to evaluate whether high or low scores are indicative of high risk in exposed. For the remaining values, a higher value means lower risk among the exposed. Traditionally, a Cohen's d with an absolute value of 0.2 is considered a small effect, 0.5 a medium effect and 0.8 or above a large effect.[3] Only results for the oldest age band are presented here, if a paper had assessed children at multiple time points using the same outcome measure.

†Not enough information to calculate Cohen's d.

‡It is unclear whether the reported results are mean (SD) or mean (SE). We have made the assumption that it is SD based on the values.

AD: Antidepressant, ADHD: Attention Deficit Hyperactivity Disorder, ASD: Autism Spectrum Disorder, ASQ: Ages and Stages Questionnaire, BNBAS: Brazelton Neonatal Behavoiral Assessment Scale, BRIEF: Behaviour Rating Inventory of Executive Function, BSID: Bayley Scales of Infant Development, CBCL: Child Behaviour Checklist, CPRS: Conners Parent Rating Scale, DNBC: Danish National Birth Cohort, DAS: Differential ability scales, HCP: Health care professional, HCP & P: Health care professional and parents, HBQ-P: MacArthur Health and Behaviour Questionnaire, MoBa: Norwegian Mother and Child Cohort, PDD: Pervasive developmental disorder, RCT: Randomised controlled trial, SDQ: Strengths and Difficulties Questionnaire, SON-R: Snijders–Oomen Niet-verbale intelligentie Test–Revisie, TCA: Tricyclic antidepressants, TIS: Teratology information service, WISC: Wechsler Intelligence Scale for Children, WPPSI: Wechsler Preschool and Primary Scale of Intelligence.

References:

1. Kontopantelis, E. & Reeves, D. METAEFF: Stata module to perform effect sizes calculations for meta-analyses. (2011).

2. StataCorp. *Stata Statistical Software: Release 15*. (StataCorp LP, 2017).

3. Fritz, C. O., Morris, P. E. & Richler, J. J. Effect size estimates: Current use, calculations, and interpretation. *J. Exp. Psychol. Gen.* **141,** 2–18 (2012).
