# Supplementary material for: Use and validity of child neurodevelopment outcome measures in studies on prenatal exposure to psychotropic and analgesic medications – A systematic review
Source: PLoS One. 2019 Jul 11;14(7):e0219778. doi: 10.1371/journal.pone.0219778 (PMC6622545; doi:10.1371/journal.pone.0219778)
Supplement: S2 Table — (PDF) [file pone.0219778.s005.pdf]

S2 Table. Study characteristics, papers on psychotropics except for antidepressants.

Reference	Country/setting	Study design	Exposure/ duration	Outcome/assessor *Name of the scale*	Sample size/exposed	Effect estimate, Exposed/unexposed Adjusted effect estimates where available	Cohen's d*
**Assessment using psychometric instruments**							
**i. Assessment by health care professional**							
Infant (<2 years)							
Platt 1989 [114]	USA/Collaborative Perinatal Project	Cohort	Antipsychotic/ long term (more than 2 months)	Motor skills /*BSID*	308/not reported	Percentage with failures on motor assessment<br>*Gross motor*<br>82.5%/69.8%<br>*Fine motor*<br>Not reported	-†
Peng 2013 [113]	China/ Second Xiangya Hospital	Cohort	Antipsychotics/ throughout pregnancy	General development/*BSID-III*	152/76	Mean (SD)	
						Motor scale<br>101.59 (8.53)/103.68 (7.19)	-0.26
						Cognitive scale<br>100.99 (8.17)/103.11 (7.84)	-0.26
						Language scale<br>97.26 (6.79)/98.18 (7.18)	-0.13
						Adaptive scale<br>99.80 (8.56)/101.24 (5.83)	-0.20
						Social-emotional scale<br>102.54 (9.72)/104.50 (8.63)	-0.26
Johnson 2012 [73]	USA/ Emory Women's Mental Health Program and community cohort	Cohort	Antipsychotics/ any (median 32 weeks)	Motor skills /*Infant Neurological International Battery*	97/21	Mean (SE)<br>63.86 (1.78)/70.12 (1.03)	-0.71
Mortensen 2003 [81]	Denmark/ National Health Service in North Jutland County	Cohort, registry based	Antipsychotics/ any duration	Motor skills / *Boel test*	818/63	OR (95% CI) of abnormal test result<br>Antipsychotics<br>4.1 (1.3 to 13.0)	0.78 High
			Benzodiazepines/any duration		837/82	Benzodiazepines<br>7.7 (3.0 to 19.3)	1.13 High

Oberlander 2004 [87]	Canada/ Reproductive mental health program at British Columbia Women's Hospital	Cohort	Clonazepam in combination with SSRI/mean duration 81 to 127 days compared to exposed to SSRI only	Cognition/*BSID-II*	46/18	Mean (SD)<br>*Motor skills*<br>93.1 (8.6)/91.5 (9.6)<br>*Mental development*<br>97.2 (4.5)/100.7 (6.4)	0.17<br>-0.61
Reebye 2002 [92]	Canada/ Children's and Women's Health Centre of British Columbia	Cohort	Clonazepam in combination with SSRI/any duration compared to exposed to SSRI only	Overall development/*BSID-II*	38/14	Mean (SD)<br>*Motor skills*<br>102 (6.3)/106 (5.4)<br>*Mental development*<br>93 (5.1)/98 (8.1)	-0.70<br>-0.70
Reebye 2012 [38]	Canada/ Reproductive Mental Health Program at St. Paul's Hospital and British Columbia's Women's Hospital	Cohort	Clonazepam in combination with SSRI/throughout pregnancy compared to exposed to SSRI only	Overall development/*BSID-II*	37/15	Mean (SD)<br>*Motor skills*<br>93 (8.6)/91 (9.5)<br>*Mental development*<br>97 (47.5)/100 (6.1)	0.22<br>-0.54
Viggedal 1993 [40]	Sweden/Gothen-burg cohort	Cohort	Benzodiazepines/more than 5 DDD in early pregnancy (not further specified)	General development/*Griffiths' mental development scale I*<br>Behaviour/*Neuropsychological assessment*	41/16	Mean (SD)<br>101.7 (7.9)/107.6 (8.3)<br>Percentage with hyperactive [inactive] symptoms<br>31% [19%]/16% [4%]<br>Percentage with short [long] attention span<br>31% [19%]/8% [4%]	-0.72<br>0.36 [High] [0.45] [High]<br>0.58 [High] [0.45] [High]
Gidai 2008a [105]	Hungary/Korányi Hospital	Cohort	Alprazolam/ high doses used for suicide attempt versus unexposed siblings	Cognition/*Hungarian development test*<br>Behaviour/*Behavioural style questionnaire*	20/9	Mean (SD)<br>96.7 (6.2)/97.3 (6.1)<br>Percentage with behavioural deviation<br>11%/27%	-0.10<br>-0.43 [High]
Gidai 2008b [106]	Hungary/Korányi Hospital	Cohort	Medazepam/ high doses used for suicide attempt versus unexposed	Cognition/*Hungarian development test*<br>Behaviour/*Behavioural style questionnaire*	19/9	Mean (SD)<br>100.0 (1.0)/98.5 (4.7)<br>Percentage with behavioural deviation<br>22%/10%	0.43<br>0.33 [High]

			siblings				
Gidai 2008c [107]	Hungary/Korányi Hospital	Cohort	Chlordiazep-oxide/high doses used for suicide attempt versus unexposed sib-lings	Cognition/*Hungarian development test*<br>Behaviour/*Behavioural style questionnaire*	52/31	Mean (SD)<br>101.4 (9.8)/95.0 (2.0)<br>Percentage with behavioural deviation<br>19%/24%	 0.83<br><br>-0.11 [High]
Timmermann 2008b [112]	Hungary/Korányi Hospital	Cohort	Meproba-mate/high doses used for suicide attempt compared to unexposed siblings	Cognition/*Hungarian development test*<br>Behaviour/*Behavioural style questionnaire*	56/31<br><br><br>54/31	Mean (SD)<br>99.2 (8.1)/97.8 (8.8)<br>Percentage with behavioural deviation<br>26%/22%	 0.17<br><br>0.10 [High]
Laegreid 1992 [108]	Sweden/Gothenburg cohort	Cohort	Benzodiazepi-nes/regular use (not defined fur-ther)	Motor skills<br>/<br>*Touwen Neurologic Assessment*<br>*Clinical neurologic assessment*	42/16	Mean (SD)<br>*Posture and motility*<br>69.56 (5.03)/72.92 (2.08)<br>Percentage with slow [stereotyped] movements<br>31% [13%]/4% [0%]<br>Percentage with normal walking pattern<br>63%/100%	 <br>-0.96<br><br>0.71 [High]<br>[0.48] [High]<br><br>-0.98
Petik 2008a [115]	Hungary/Korányi Hospital	Cohort	Glutethimide/high doses used for suicide attempt compared to un-exposed siblings	Cognition/*Hungarian development test*<br>Behaviour/*Behavioural style questionnaire*	26/14<br><br><br>22/12	Mean (SD)<br>100.4 (10.8)/98.8 (9.6)<br>Percentage with behavioural deviation<br>33%/30%	 0.16<br><br>0.07 [High]
Petik 2008b [116]	Hungary/Korányi Hospital	Cohort	Amobarbital/high doses used for suicide attempt compared to un-exposed siblings	Cognition/*Hungarian development test*<br>Behaviour/*Behavioural style questionnaire*	21/11<br><br><br>13/8	Mean (SD)<br>96.4 (8.4)/100.0 (12.2)<br>Percentage with behavioural deviation<br>40%/60%	 -0.35<br><br>-0.46 [High]
Timmermann 2008a [117]	Hungary/Korányi Hospital	Cohort	Barbital, hexobar-bital, butobarbi-tal/high doses used for suicide attempt compared	Cognition/*Hungarian development test*	 8/5<br><br>8/4	Mean (SD)<br>Barbital<br>90.8 (18.8)/95.0 (8.7)<br>Hexobarbital<br>92.5 (8.7)/96.3 (7.5)	 <br>-0.26<br><br>-0.47

			to unexposed siblings		6/3	Butobarbital<br>100.0 (0)/ 100.0 (0)	undefined
				Behaviour/ *Behavioural style questionnaire*		Percentage with behavioural deviation	
					5/3	Barbital<br>33%/100%	-2.24 [High]
					8/4	Hexobarbital<br>25%/25%	0.00 [High]
					6/3	Butobarbital<br>33%/66%	-0.71 [High]
Preschool (2-5 years)							
Hurault-Delarue 2016 [69]	France/ EFEMERIS database	Cohort, registry based	Antipsychotics/ any in 2nd or 3rd trimester	Overall development/*Compulsory medical exam*	32 373/70	Percentage with delayed development<br>Antipsychotics<br>*Motor*<br>2.1%/0.4%<br>*Mental*<br>2.1%/1.0%	 <br><br>0.10 [High]<br><br>0.06 [High]
			Anxiolytics, including hypnotics/any in 2nd or 3rd trimester		32 303/281	Anxiolytics<br>*Motor*<br>1.7%/0.4%<br>*Mental*<br>1.7%/1.0%	 <br>0.08 [High]<br><br>0.04 [High]
Schechter 2017 [94]	USA/ Emory Women's Mental Health Program and community cohort	Cohort	Antipsychotics/ any duration<br><br>Anxiolytics/ any duration<br>Hypnotics/ any duration	Cognition/*DAS*	162/not reported	Pearson correlation<br>Antipsychotics<br>-0.09<br>Anxiolytics<br>-0.19<br>Hypnotics<br>-0.04	-†
Hartz 1975 [35]	USA/Collaborative Perinatal Project	Cohort	Meprobamate, chlordiazepoxide/any duration	Cognition/*Stanford Binet Intelligence Scale*	28 453/694<br><br><br>28 453/435	Mean<br>Meprobamate<br>96.7/96.9<br>Chlordiazepoxide<br>96.7/96.9	-†
School child (6-12 years)							
Platt 1989 [114]	USA/Collaborative	Cohort	Antipsychotic/	Motor skills	308/not	Not reported, as only statistically signifi-	-†

	Perinatal Project		long term (more than 2 months)	/*Paediatric neurologic assessment*	reported	cant results are reported.	
Adolescent (13-18 years)							
Mattson 2002 [79]	USA/ California TIS and Clinical Research Program	Cohort	Benzodiazepi-nes/at least one 1st trimester exposure	Cognition/ *WISC-III*	107/25	Mean<br>100.2/105.7	-†
**ii. Assessment by parents**							
Infant (<2 years)							
Reebye 2002 [92]	Canada/ Children's and Women's Health Centre of British Columbia	Cohort	Clonazepam in combination with SSRI/any duration compared to exposed to SSRI only	Behaviour/*Early Infancy Temperament Questionnaire*	38/14	Percentage with difficult temperament<br>31%/0%	0.80 High
Preschool (2-5 years)							
Lupattelli 2019 [109]	Norway/MoBa	Cohort	Benzodiazepi-nes/any in mid-pregnancy (week 17-28) or late pregnancy (week 29 and later)	Motor skills/*ASQ*	4 183/28	Beta (95% CI)<br>*Gross motor*<br>Mid<br>-0.47 (-0.78 to -0.17)	-0.57 High
						Late<br>0.80 (0.12 to 1.48)	0.48 High
						Fine motor<br>Mid<br>-0.30 (-0.76 to 0.15)	-0.25 High
						Late<br>0.74 (-0.12 to 1.60)	0.36 High
				Language/*ASQ*		*Communication*<br>Mid<br>-0.15 (-0.43 to 0.13)	-0.20 High
						Late<br>0.06 (-0.16 to 0.27)	0.11 High
				Behaviour/*CPRS-R*		*ADHD traits*<br>Mid<br>-0.24 (-0.63 to 0.15)	-0.23 High
						Late	

						0.29 (0.02 to 0.57)	0.43 [High]
			z-hypnotics/ any in midpregnancy (week 17-28) or late pregnancy (week 29 and later)			*Gross motor*	
						Mid	
						0.03 (-0.74 to 0.80)	0.02 [High]
						Late	
						0.93 (0.28 to 1.58)	0.57 [High]
						Fine motor	
						Mid	
						0.32 (-0.68 to 1.32)	0.13 [High]
						Late	
						0.58 (-0.44 to 1.59)	0.23 [High]
						Communication	
						Mid	
						-0.27 (-0.76 to 0.23)	-0.23 [High]
						Late	
						0.49 (-0.01 to 0.98)	0.42 [High]
						ADHD traits	
						Mid	
						0.11 (-0.45 to 0.67)	0.08 [High]
						Late	
						-0.10 (-0.49 to 0.29)	-0.10 [High]
Brandlistuen 2017 [104]	Norway/MoBa	Cohort	Benzodiazepi-nes/ any duration	Behaviour/*CBCL, short-ened*	13 734/100	Beta (95% CI)<br>Benzodiazepines	
						Externalising	
						0.16 (-0.17 to 0.48)	0.10 [High]
						Internalising	
						0.26 (0.002 to 0.52)	0.20 [High]
			z-hypnotics/any			z-hypnotics	
					13 693/59	*Externalising*	
						-0.52 (-0.87 to -0.15)	-0.37 [High]
						Internalising	
						0.05 (-0.24 to 0.35)	0.04 [High]
Misri 2006 [80]	Canada/ Reproductive mental health program at British Columbia Women's Hospital	Cohort	Clonazepam in combination with SSRI/<br>mean duration 137 days compared to exposed to SSRI	Behaviour and emotion-ality/*CBCL*	22/9	Mean (SD)<br>*Internalising*	
						59.56 (11.20)/53.31 (10.14)	0.59 [High]
						Somatic complaints	
						58.78 (7.76)/54.62 (5.78)	0.63 [High]
						Withdrawn	

			only			58.44 (9.10)/54.92 (6.22)<br>*Emotionally reactive*<br>61.00 (15.17)/55.08 (7.21)<br>*Anxious/depressed*<br>59.11 (10.80)/56.46 (7.46)	0.47 [High]<br><br>0.53 [High]<br><br>0.30 [High]
Odsbu 2015 [110]	Norway/MoBa	Cohort	Benzodiazepines/one period and two/three periods	Language/ *Language competence questionnaire*	51 411/316<br><br><br><br>51 174/79	OR (95%) of low language competence<br>One period<br>1.0 (0.7 to 1.3)<br>Two/three periods<br>1.3 (0.8 to 2.3)	 <br>0.00 [High]<br><br>0.14 [High]
School child (6-12 years)							
Radojčić 2017 [111]	The Netherlands/ Generation R	Cohort	Benzodiazepines/any	Behaviour/*CBCL*	4 553/79	Beta (95% CI)<br>*ODD subscale*<br>0.23 (-0.30 to 0.76)<br>*Anxiety subscale*<br>0.35 (-0.13 to 0.83)	 <br>0.10 [High]<br><br>0.16 [High]
**iii. Assessment by teachers/others**							
Preschool (2-5 years)							
Misri 2006 [80]	Canada/ Reproductive mental health program at British Columbia Women's Hospital	Cohort	Clonazepam in combination with SSRI/ mean duration 137 days compared to exposed to SSRI only	Behaviour and emotionality/teachers *CBCL*	22/9	*Internalising*<br>52.17 (14.16)/53.73 (7.77)<br>*Somatic complaints*<br>52.33 (5.72)/52.91 (4.30)<br>*Withdrawn*<br>55.33 (5.68)/54.82 (4.67)<br>*Emotionally reactive*<br>57.50 (8.19)/54.64 (7.53)<br>*Anxious/depressed*<br>59.17 (10.40)/56.64 (7.31)	 -0.14 [High]<br><br>-0.12 [High]<br><br>0.10 [High]<br><br>0.37 [High]<br><br>0.29 [High]
School child (6-12 years)							
Radojčić 2017 [111]	The Netherlands/ Generation R	Cohort	Benzodiazepines/any duration	Behaviour/teachers *CBCL*<br><br><br><br><br>Behaviour/parent and	3 225/62<br><br><br><br><br>5 287/96	Beta (95% CI)<br>*ODD subscale*<br>0.37 (-0.07 to 0.82)<br>*Anxiety subscale*<br>0.10 (-0.25 to 0.44)<br>*Aggressive behaviour*	 <br>0.21 [High]<br><br>0.07 [High]

				teacher, *CBCL*		0.75 (-0.28 to 1.77)	0.15 [High]
Assessment using medical diagnosis							
Figueroa 2010 [56]	USA/MarketScan claims data	Cohort, registry based	Benzodiazepines, any	ADHD/diagnosis	36 925/311	OR (95% CI) 1.82 (0.86 to 3.85)	0.33 [High]
Janecka 2018 [72]	Israel/Meuhedet health maintenance organisation	Case-control, registry based	Lithium/any	ASD	1 426/21	HR (95% CI) 4.23 (0.58 to 30.9)	0.82 [High]

Reference numbers in brackets refer to the reference list in the article.

* Effect sizes (Cohen's d) were calculated using the metaeff package (1) for Stata (2). [High] indicates that higher/more positive values mean a higher risk among the exposed. For the remaining values, a higher value means lower risk among the exposed. Traditionally, a Cohen's d with an absolute value of 0.2 is considered a small effect, 0.5 a medium effect and 0.8 or above a large effect.(3) Only results for the oldest age band are presented here, if a paper had assessed children at multiple time points using the same outcome measure.

† Not enough information to calculate Cohen's d.

ADHD: Attention Deficit Hyperactivity Disorder, BRIEF: Behaviour Rating Inventory of Executive Function, ASQ: Ages and Stages Questionnaire, BSID: Bayley Scales of Infant Development, CBCL: Child Behaviour Checklist, CPRS:R: Conners' Parent Rating Scale, revised, DAS: Differential ability scales, MoBa: Norwegian Mother and Child Cohort, ODD: Oppositional defiant disorder, RCT: Randomised controlled trial, SDQ: Strengths and Difficulties Questionnaire, TIS: Teratogen information service, WISC: Wechsler Intelligence Scale for Children.

References:

1. Kontopantelis E, Reeves D. METAEFF: Stata module to perform effect sizes calculations for meta-analyses [Internet]. 2011 [cited 2018 Jul 6]. Available from: http://EconPapers.repec.org/RePEc:boc:bocode:s457072

2. StataCorp. Stata Statistical Software: Release 15. College Station, TX: StataCorp LP; 2017.

3. Fritz CO, Morris PE, Richler JJ. Effect size estimates: Current use, calculations, and interpretation. J Exp Psychol Gen. 2012;141(1):2–18.
