# Supplementary material for: Use and validity of child neurodevelopment outcome measures in studies on prenatal exposure to psychotropic and analgesic medications – A systematic review
Source: PLoS One. 2019 Jul 11;14(7):e0219778. doi: 10.1371/journal.pone.0219778 (PMC6622545; doi:10.1371/journal.pone.0219778)
Supplement: S3 Table — (PDF) [file pone.0219778.s006.pdf]

S3 Table. Study characteristics, papers on analgesics.

Reference	Country/setting	Study design	Exposure/ duration	Outcome/*Name of the scale*	Sample size/exp osed	Effect estimate, Exposed/unexposed Adjusted estimates where available	Cohen's d*
**Assessment using psychometric instruments**							
**i. Assessment by health care professional**							
Infant (<2 years)							
Salokorpi 1996 [136]	Finland/Follow-up in RCT	RCT	Indomethacin/ any duration	Overall development/*Autti-Rämö neurodevelopmental test battery*	44/25	Percentage with subnormal scores<br>*Development*<br>52%/42%<br>*Motor*<br>52%/42%<br>*Verbal*<br>20%/5%<br>*Cognition*<br>8%/5%	 <br>0.20 High<br><br>0.20 High<br><br>0.47 High<br><br>0.11 High
Amin 2008 [134]	Canada/Health Sciences Centre, Winnipeg	Cohort, registry based	Indomethacin/ any duration	Overall development/*Gesell development scales, revised*	23/12	Mean (SD)<br>*Gross motor*<br>97 (13)/97 (13)<br>*Fine motor*<br>98 (7)/99 (8)<br>*Language*<br>101 (8)/101 (6)<br>*Adaptive*<br>99 (8)/101 (6)<br>Percentage with risk of neuroabnor-mality<br>0%/9%	 <br>0.00<br><br>-0.13<br><br>0.00<br><br>-0.28<br><br><br>-0.44 High
Al-Alaiyan 1996 [133]	USA/University of Maryland Medical Centre	Cohort, registry based	Indomethacin/ any duration	Cognition/*BSID-II, mental development index*	87/29	OR 0.44 (95% CI 0.12 to 1.50)	-0.45 High
Avella-Garcia 2016 [118]	Spain/INfancia y Medio Ambiente project	Cohort	Paracetamol/ any	Overall development/*BSID, unspecified edition*	2 195/955	Beta (95% CI)<br>0.75 (-0.75 to 2.25)	 0.04

Preschool (2-5 years)							
Barr 1990 [137]	USA/ Two unspecified study hospitals	Cohort	ASA/any and frequent use (several times a week or more)	Motor skills/*items from Gross Motor Scale (University of Oregon Medical School), Gesell – and Bayley Scales, Wisconsin Fine Motor Steadiness Battery, and Halstead Reitan Neuropsychological Battery*	449/not reported	*Gross motor*<br>ASA, ever, about 1/4 of a SD lower on balance (p = .004)<br>*Fine motor*<br>ASA, frequent use, about ½ of a SD more fine motor steadiness errors (p = .001) and about 1/3 of a SD lower scores on the fine motor examiner rating (p = .047)	-*
Klebanoff 1988 [138]	USA/birth cohort	Cohort	ASA/any in the first 20 weeks of pregnancy	Cognition/ *Stanford Binet Intelligence Scale*	19 226/10 159	Mean difference (SE)<br>0.9 (0.29)	-*
Streissguth 1987 [39]	USA/birth cohort	Cohort	ASA/any duration<br>Paracetamol/any during first 5 months of pregnancy	Cognition/*WPPSI*	421/192<br><br><br>412/183	Mean difference (SE)<br>ASA<br>-2.67 (0.77)<br>Paracetamol<br>0.28 (0.54)	-*
Avella-Garcia 2016 [118]	Spain/INfancia y Medio Ambiente project	Cohort	Paracetamol/<br>any duration	Overall development/*McCarthy Scales of Children's Abilities*<br>Autism/*CAST†*	2 001/828<br><br><br><br>1 467/644	Beta (95% CI)<br>*MSCA*<br>-0.21 (-1.70 to 1.28)<br>*CAST*<br>0.08 (-0.28 to 0.44)	-0.01<br><br>0.02 [High]
Bornehag 2018 [119]	Sweden/SELMA study	Cohort	Paracetamol/any 1st trimester exposure	Language/*Swedish language development scale‡*	754/446	OR 1.26 (95% CI 0.72 to 2.19)	0.13 [High]
Liew 2016b [124]	Denmark/DNBC substudy	Cohort	Paracetamol/any duration	Behaviour/*TEACh-5*	1 491/881	Mean difference (95% CI)<br>-0.10 (-0.26 to 0.05)	-0.07
Liew 2016c [125]	Denmark/DNBC substudy	Cohort	Paracetamol/any duration	Cognition/*WPPSI-R, shortened*	1 491/881	Mean difference (95% CI)<br>-1.50 (-3.40 to 0.46)	-0.08
School child (6-12 years)							
Markovic 2019 [135]	The Netherlands/ Generation R	Cohort	NSAIDs	Cognition/*SON-R*	5 091/362	Mean difference (95% CI)<br>-0.32 (-1.82 to 1.19)	-0.02

Laue 2019 [121]	Canada/GESTE	Cohort	Paracetamol/any in third trimester as detected in meconium	Cognition/*WISC-IV*		Mean difference (95% CI)	
						Coding	
					87/32	Low level	
						1.30 (0.06 to 2.54)	0.46
					86/31	High level	
						0.74 (-0.54 to 2.01)	0.26
						Digit span	
						Low level	
						0.78 (-0.31 to 1.88)	0.31
						High level	
						-0.09 (-1.22 to 1.03)	-0.04
						Block design	
						Low level	
						1.03 (-0.22 to 2.29)	0.36
						High level	
						-0.92 (-2.20 to 0.37)	-0.32
						Information	
						Low level	
						-0.09 (-1.00 to 0.83)	-0.04
						High level	
						0.27 (-0.67 to 1.21)	0.13
						Vocabulary	
						Low level	
						0.78 (-0.35 to 1.92)	0.30
						High level	
						0.10 (-1.07 to 1.27)	0.04
**ii. Assessment by parents**							
Infant (<2 years)							
Vlenterie 2016 [42]	Norway/MoBa	Cohort	Paracetamol/ long term (28 days or more)	Motor skills/*Motor milestone questionnaire ASQ*	3 260/1 630	OR (95% CI)	
						Motor milestones	
						1.35 (1.07 to 1.70)	0.17 [High]
						Gross motor	
						1.28 (0.83 to 1.98)	0.14 [High]
						Fine motor	
						1.12 (0.91 to 1.38)	0.06 [High]
				Language/*ASQ*		*Communication*	
						1.38 (0.98 to 1.95)	0.18 [High]

				Behaviour/*CBCL*		*Externalising*<br>1.07 (0.85 to 1.35)	0.04 High
						Internalising<br>1.04 (0.80 to 1.35)	0.02 High
				EAS (activity, sociability, shyness)		*Activity*<br>1.09 (0.87 to 1.38)	0.05 High
						Sociability<br>1.18 (0.92 to 1.51)	0.09 High
						Shyness<br>1.20 (0.86 to 1.66)	0.10 High
				Emotionality/*EAS (emo-tionality)*		*Emotionality*<br>0.77 (0.55 to 1.07)	-0.14 High
Wood 2016a [140]	Norway/MoBa	Cohort	Triptans/ any dura-tion versus unmedi-cated migraine	Motor skills/*ASQ*	4 545/49 5	Percentage with subnormal scores<br>*Gross motor*<br>1.6%/2.3%	-0.05 High
						Fine motor<br>13.9%/11.5%	0.07 High
				Language/*ASQ*		*Communication*<br>3.4%/3.8%	-0.02 High
				Behaviour/*CBCL*		*Externalising*<br>11.0%/8.1%	0.09 High
						Internalising<br>8.1%/8.7%	-0.02 High
				EAS (activity, sociability, shyness)		*Activity*<br>9.8%/9.2%	-0.02 High
						Sociability<br>8.8%/9.3%	-0.01 High
						Shyness<br>4.9%/4.0%	0.04 High
				Emotionality/*EAS (emo-tionality)*		*Emotionality*<br>3.2%/5.1%	-0.10 High
Preschool (2-5 years)							
Markovic 2019 [135]	The Netherlands/ Generation R	Cohort	NSAIDs	Behaviour/*CBCL*	5 244/37 2	Mean difference (95% CI)<br>0.05 (-0.13 to 0.23)	0.03 High
Brandlistuen 2013 [120]	Norway/MoBa	Cohort, sibling controlled	Ibuprofen/any dura-tion	Motor skills/*Motor mile-stone questionnaire & ASQ*	155 sibling pairs	Beta (95% CI)<br>Ibuprofen<br>*Motor milestones*	

						0.14 (-0.03 to 0.30)	0.19 [High]
						Gross motor	
						0.02 (-0.16 to 0.21)	0.02 [High]
						Fine motor	
						0.12 (-0.02 to 0.25)	0.20 [High]
				Language/*ASQ*		*Communication*	
						0.01 (-0.16 to 0.19)	0.01 [High]
				Behaviour/*CBCL*		*Externalising behaviour*	
						0.09 (-0.03 to 0.20)	0.17 [High]
						Internalising behaviour	
						0.04 (-0.03 to 0.20)	0.08 [High]
			Paracetamol/ long term (28 days or more)	Motor skills/*Motor milestone questionnaire & ASQ*		Paracetamol<br>*Motor milestones*	
						0.26 (0.06 to 0.45)	0.32 [High]
						Gross motor	
						0.24 (0.12 to 0.51)	0.30 [High]
						Fine motor	
						0.05 (-0.12 to 0.21)	0.07 [High]
				Language/*ASQ*		*Communication*	
						0.20 (0.01 to 0.39)	0.25 [High]
				Behaviour/*CBCL*		*Externalising behaviour*	
						0.24 (0.12 to 0.37)	0.46 [High]
						Internalising behaviour	
						0.14 (0.01 to 0.28)	0.25 [High]
				EAS (activity, sociability, shyness)		*Activity*	
						0.22 (0.11 to 0.36)	0.42 [High]
						Sociability	
						-0.01 (-0.15 to 0.13)	-0.02 [High]
						Shyness	
						0.01 (-0.04 to 0.24)	0.02 [High]
				Emotionality/*EAS (emotionality)*		*Emotionality*	
						0.10 (-0.04 to 0.24)	0.17 [High]
Liew 2014 [122]	Denmark/DNBC	Cohort	Paracetamol/<br>any and long term<br>(all 3 trimesters)	Behaviour/*SDQ*	Any use	RR (95% CI)	
					40 811/2	*Total difficulties*	
					2 623	Any use	
					Long	1.13 (1.01 to 1.27)	0.07 [High]
					term	Long term	
					22 288/4	1.24 (1.03 to 1.48)	0.12 [High]

					100	*Prosocial*, Any use<br>1.00 (0.88 to 1.15)	0.00 High
Liew 2016b [124]	Denmark/DNBC substudy	Cohort	Paracetamol/any	Behaviour/*BRIEF*	1 491/881	Mean difference (95% CI)<br>*BRIEF, parent*<br>0.78 (-0.46 to 2.00)	0.07 High
Skovlund 2017 [128]	Norway/MoBa	Cohort	Paracetamol/ three periods of pregnancy	Language/ *Intelligibility/Complexity of 3-year-old Children's Utterances & ASQ*	36 572/1617	OR (95% CI)<br>Paracetamol<br>*Lower language competence*<br>1.04 (0.93 to 1.17)<br>*Lower communication skills*<br>1.16 (1.04 to 1.28)	0.02 High<br>0.08 High
			Analgesic opioids/longer term (two or three periods of pregnancy)		45 646/161	Opioids<br>*Lower language competence*<br>0.91 (0.62 to 1.35)<br>*Lower communication skills*<br>1.12 (0.82 to 1.53)	-0.05 High<br>0.06 High
Wood 2016b [141]	Norway/MoBa	Cohort	Triptans/any duration compared to unmedicated migraine	Behaviour/*CBCL*	3 687/396	RD (95% CI)<br>*Externalising*<br>0.02 (-0.01 to 0.05)<br>*Internalising*<br>-0.01 (-0.03 to 0.01)	0.07 High<br>-0.05 High
Wood 2016c [142]	Norway/MoBa	Cohort	Triptans/any duration compared to unmedicated migraine	Motor skills/A*SQ*	4 204/375	OR (95% CI)<br>*Gross motor*<br>0.45 (0.14 to 1.42)<br>*Fine motor*<br>1.25 (0.89 to 1.74)	-0.44 High<br>0.12 High
				Language/*ASQ*		*Communication*<br>0.93 (0.31 to 2.81)	-0.04 High
				Behaviour/*EAS (activity, sociability, shyness)*		Beta (95% CI)<br>*Activity*<br>0.03 (-0.11 to 0.17)<br>*Sociability*<br>0.01 (-0.13 to 0.14)<br>*Shyness*<br>0.04 (-0.06 to 0.18)	0.02 High<br>0.00 High<br>0.04 High
				Emotionality/*EAS (emo-*		*Emotionality*	

				tionality)		-0.01 (-0.13 to 0.11)	-0.01 [High]
Harris 2018 [139]	Norway/MoBa	Cohort	Triptans/any duration compared to unmedicated migraine	Language/*ASQ*<br>Behaviour/*CBCL*<br>Behaviour/*EAS (activity, sociability, shyness)*<br>Emotionality/*EAS (emotionality)*	1 826/347<br>1 797/340<br>1 825/343<br>1 844/351<br>1 841/349<br>1 828/348<br>1 828/345	RR (95% CI)<br>0.77 (0.50 to 1.18)<br>*Externalising*<br>0.68 (0.44 to 1.05)<br>*Internalising*<br>0.97 (0.68 to 1.37)<br>Beta (95% CI)<br>*Activity*<br>-0.06 (-1.35 to 1.23)<br>*Sociability*<br>1.66 (0.30 to 3.02)<br>*Shyness*<br>-0.71 (-2.08 to 0.65)<br>*Emotionality*<br>-1.02 (-2.33 to 0.29)	-0.16 [High]<br>-0.23 [High]<br>-0.02 [High]<br>-0.01 [High]<br>0.14 [High]<br>-0.06 [High]<br>-0.09 [High]
School child (6-12 years)							
Stergiakouli 2016 [129]	UK/ALSPAC	Cohort	Paracetamol/any in the past three months assessed at 18th week and 32nd week of pregnancy	Behaviour/*SDQ*	7 796/4415<br>7 796/3381	RR (95% CI)<br>*Total difficulties*<br>18th week<br>1.01 (0.79 to 1.27)<br>32nd week<br>1.37 (1.07 to 1.75)<br>*Prosocial*<br>18th week<br>1.03 (0.92 to 1.15)<br>32nd week<br>1.06 (0.94 to 1.19)	0.01 [High]<br>0.19 [High]<br>0.02 [High]<br>0.04 [High]
Tovo-Rodrigues 2018 [131]	Brazil/Pelotas birth cohort	Cohort	Paracetamol/any	Behaviour/*SDQ*	3 447/959	OR (95% CI)<br>*Total difficulties*<br>1.19 (0.94 to 1.50)	0.10 [High]

						Low prosocial score<br>0.79 (0.36 to 1.75)	-0.13 High
Thompson 2014 [130]	New Zealand/ABC study	Cohort	ASA/any duration	Behaviour/*SDQ*	614/33	Mean difference (95% CI)<br>ASA	
			Paracetamol/any duration		614/306	0.6 (-1.8 to 3.0)<br>Paracetamol	0.09 High
						Total difficulties<br>0.8 (-0.1 to 1.8)	0.13 High
						Prosocial<br>-0.1 (-0.4 to 0.3)	-0.05
				CPRS:R-L, ADHD index		*ADHD index*<br>0.5 (-1.1 to 2.1)	0.05 High
				CPRS:R-L, DSM-IV total		*DSM-IV ADHD criteria*<br>0.8 (-0.7 to 2.3)	0.08 High
				CPRS:R-L, Conners' global indices		*General problem behaviour*<br>0.1 (-1.5 to 1.8)	0.01 High
Ruisch 2018 [127]	UK/ALSPAC	Cohort	ASA/any duration	Behaviour/*Development and Well-Being Assessment*		IRR (98.3% CI)<br>ASA	
					6772/not reported	*ODD*<br>1.05 (0.84 to 1.31)	-*
					6775	*CD*<br>1.02 (0.85 to 1.23)	-*
			Paracetamol/any duration			Paracetamol	
					4652	*ODD*<br>1.00 (0.84 to 1.31)	-*
					4645	*CD*<br>1.09 (0.96 to 1.23)	-*
**iii. Assessment by teachers/others**							
Preschool (2-5 years)							
Avella-Garcia 2016 [118]	Spain/INfancia y Medio Ambiente project	Cohort	Paracetamol/ any	Behaviour/teacher *California Preschool Social Competence Scale*	1 382/828	Beta (95% CI)<br>*CPSCS*<br>-1.15 (-3.16 to 0.87)	-0.05
				ADHD, DSM-IV form list		IRR (95%CI)<br>*ADHD list*<br>1.25 (0.93 to 1.69)	0.13 High
Liew 2016b	Denmark/DNBC	Cohort	Paracetamol/any	Behaviour/teacher	1 292/77	Mean difference (95% CI)	

[124]	substudy			*BRIEF*	2	*BRIEF, teacher*<br>0.41 (-0.82 to 1.65)	0.04 [High]
School child (6-12 years)							
Markovic 2019 [135]	The Netherlands/ Generation R	Cohort	NSAIDs	Behaviour/teacher *CBCL*	3 787/27 6	Mean difference (95% CI)<br>-0.24 (-1.23 to 0.76)	-0.03 [High]
Thompson 2014 [130]	New Zealand/ABC study	Cohort	ASA/any duration	Behaviour/children *SDQ*	614/33	Mean difference (95% CI)<br>ASA<br>0.3 (-1.8 to 2.4)	0.05 [High]
			Paracetamol/any duration		614/306	Paracetamol<br>*Total difficulties*<br>1.1 (0.2 to 2.0)	0.19 [High]
						Prosocial<br>0.00 (-0.03 to 0.30)	0.00
Ruisch 2018 [127]	UK/ALSPAC	Cohort	ASA/any duration	Behaviour/teacher *Development and Well-Being Assessment*		IRR (98.3% CI)<br>ASA	
					2898/not reported	*ODD*<br>0.99 (0.64 to 1.52)	-*
					4794	*CD*<br>1.03 (0.66 to 1.61)	-*
			Paracetamol/any duration		2898	Paracetamol<br>*ODD*<br>1.25 (1.02 to 1.53)	-*
					2894	*CD*<br>1.09 (0.80 to 1.49)	-*
Assessment using medical diagnosis							
Rubenstein 2019 [143]	USA/Study to Explore Early Development	Case-control	Opioids/any	Developmental delay	2 515/not reported	OR (95% CI)<br>1st trimester<br>1.06 (0.50 to 2.28)	0.03 [High]
						2nd trimester<br>1.64 (0.87 to 3.12)	0.27 [High]
						3rd trimester<br>0.85 (0.52 to 1.41)	-0.09 [High]
				ASD		1st trimester<br>1.58 (0.85 to 2.94)	0.25 [High]
					2 946	2nd trimester<br>1.13 (0.61 to 2.12)	0.07 [High]

						3rd trimester<br>1.02 (0.66 to 1.57)	0.01 [High]
Janecka 2018 [72]	Israel/Meuhedet health maintenance organisation	Case-control, registry based	Triptans/any	ASD	1 478/73	HR (95% CI)<br>Triptans<br>0.66 (0.09 to 4.83)	-0.23 [High]
			Paracetamol/any		5 221/3 871	Paracetamol<br>0.83 (0.62 to 1.10)	-0.10 [High]
Liew 2016a [123]	Denmark/DNBC	Cohort	Paracetamol/ any duration	Infantile autism	64 322/3 6 187	HR (95% CI)<br>*Infantile autism without hyperkinetic symptoms*<br>0.98 (0.77 to 1.26)	-0.01 [High]
						Infantile autism with hyperkinetic symptoms	0.24 [High]
				ASD		1.55 (0.98 to 2.45)<br>*ASD without hyperkinetic symptoms*	0.04 [High]
						1.07 (0.92 to 1.24)<br>*ASD with hyperkinetic symptoms*	0.23 [High]
						1.51 (1.19 to 1.92)	
Liew 2014 [122]	Denmark/DNBC	Cohort	Paracetamol/ any and long term (all 3 trimesters)	Hyperkinetic disorder	64 322/3 6 187	HR (95% CI)<br>Any use<br>1.37 (1.19 to 1.59)	0.18 [High]
						Long term<br>1.61 (1.30 to 2.01)	-*
Liew 2019 [126]	USA/Nurses' Health Study II	Cohort	Paracetamol/ weekly use	ADHD	2 814/37 3	OR (95% CI)<br>1.42 (0.96 to 2.10)	0.19 [High]
Ystrom 2017 [132]	Norway/MoBa	Cohort, follow-up in registries	Paracetamol/ any in pregnancy and long term (29 days or more of use)	ADHD	93 216/4 7 601<br>46 649/1 034	HR (95% CI)<br>Any<br>1.12 (1.02 to 1.24)	0.07 [High]
						Long term<br>2.20 (1.50 to 3.24)	0.46 [High]

Reference numbers in brackets refer to the reference list in the article.

* Effect sizes (Cohen's d) were calculated using the metaeff package [1] for Stata [2]. [High] indicates that higher/more positive values mean a higher risk among the exposed. For the remaining values, a higher value means lower risk among the exposed. Traditionally, a Cohen's d with an absolute value of 0.2 is considered a small effect, 0.5 a medium effect and 0.8 or above a large effect.[3] Only results for the oldest age band are presented here, if a paper had assessed children at multiple time points using the same outcome measure.

†Not enough information to calculate Cohen's d.
‡Structured interview of parents by the health care professional.
[i] Mixture of parental questionnaires and nurse observation.
ABC study: Auckland Birthweight Collaborative study, ADHD: Attention Deficit Hyperactivity Disorder, ALSPAC: Avon Longitudinal Study of Parents and Children, ASA: Acetylsalicylic acid, ASD: Autism Spectrum Disorder, ASQ: Ages and Stages Questionnaire, BRIEF: Behaviour Rating Inventory of Executive Function, BSID: Bayley Scales of Infant Development, CAST: Childhood Autism Spectrum Test, CBCL: Child Behaviour Checklist, CD: Conduct disorder, CPRS:R-L: Conners' Parent Rating Scale, revised, long format, DNBC: Danish National Birth Cohort, EAS: Emotionality, Activity, Sociability Temperament Survey, GESTE: Gestation and the Environment, HCP: Health care professional, HCP & P: Health care professional and parents, MoBa: Norwegian Mother and Child Cohort, ODD: Oppositional defiant disorder, RCT: Randomised controlled trial, SDQ: Strengths and Difficulties Questionnaire, SELMA study: Swedish Environmental Longitudinal, Mother and child, Asthma and Allergy study, SON-R: Snijders–Oomen Niet-verbale intelligentie Test–Revisie, TEACh-5: Test of everyday attention, 5 years, WISC: Wechsler Intelligence Scale for Children, WPPSI: Wechsler Preschool and Primary Scale of Intelligence.

References:

1. Kontopantelis, E. & Reeves, D. METAEFF: Stata module to perform effect sizes calculations for meta-analyses. (2011).

2. StataCorp. *Stata Statistical Software: Release 15*. (StataCorp LP, 2017).

3. Fritz, C. O., Morris, P. E. & Richler, J. J. Effect size estimates: Current use, calculations, and interpretation. *J. Exp. Psychol. Gen.* **141,** 2–18 (2012).
