# Supplementary material for: Use and validity of child neurodevelopment outcome measures in studies on prenatal exposure to psychotropic and analgesic medications – A systematic review
Source: PLoS One. 2019 Jul 11;14(7):e0219778. doi: 10.1371/journal.pone.0219778 (PMC6622545; doi:10.1371/journal.pone.0219778)
Supplement: S4 Table — (PDF) [file pone.0219778.s007.pdf]

S4 Table. Reliability and validity of the outcome measures used in studies on neurodevelopmental safety of prenatal exposure to psychotropics and analgesics.

Outcome measure	Number of studies	Validity
**Assessments using psychometric instruments**		
**i. Assessment by health care professionals**		
Infants (<2 years)		
Autti-Rämö neurodevelopmental test battery	1	Standardised on 104 Finnish children<br>No information on reliability<br>Validity: A correlation of r=0.045, p<0.001 between language score at 18 months and verbal IQ at 5.5 years. Mental score at 18 months had a correlation of r=0.47, p<0.001 with verbal IQ at 5.5 years[1]
Bayley infant neurodevelopmental screener (BINS)	1	Standardised on 600 children and a clinical sample of 303 children<br>Reliability: Internal consistency α=0.73 to 0.85. Test-retest reliability from 0.71 at 3 months to 0.84 at 18 months<br>Validity: Correlation to the BSID-II, MDI, ranged from 0.43 at 3 months to 0.82 at 24 months. Correlation with BSID-II, PDI ranged from 0.39 at 3 months to 0.58 at 24 months[2]
Behavioural style questionnaire (Hungarian)	7	Only available in Hungarian
BNBAS	1	Information on validity not found/not publicly available
Boel test	1	Information on validity not found/not publicly available
BSID-III	4/16*	Standardised on 1700 children<br>Reliability: Internal consistency 0.91 to 0.93. Test-retest reliability 0.78 to 0.81<br>Validity: Correlation of language and cognition scales to WPPSI-III 0.79 to 0.82, correlation of motor scales with Peabody Developmental Motor Scales 0.49 to 0.57. Not intended to predict later function. Predictive validity has only been tested in preterm children and specificity was high, though sensitivity was low[3]
Gesell development scales, revised	1/3*	Information on validity not found/not publicly available
Griffiths' mental development scale I	1	Interrater reliability between 0.60 and 1.00 in 78% of cases<br>No information on validity[4]
Hungarian development test	7	Only available in Hungarian
Infant neurological international battery	1	Standardised on 308 infants<br>Reliability: Interrater reliability between 0.61 and 0.80<br>Validity: Predictive validity was between 0.40 and 0.60[5]
Psychomotor, Prechtl	1	Reliability: Inter-rater reliability 0.89 to 0.93<br>Validity: Sensitivity 0.94, specificity 0.48 in preterm and newborns to 0.82-1.00 at 3 months of age[6]
Reynell developmental language scale	2	Information on validity not found/not publicly available
Touwen Infant Neurologic Examination	1	Reliability: Interrater reliability 0.83<br>Validity: Assessment at 4, 6, 10 or 12 months predicted complex minor neurological disorder or cerebral paresis at 18 months with sensitivities of 0.83 to 0.91 and specificities 0.62 to 0.84[7]
Preschool (2 to 5 years)		
BSID-III	2/6*	Standardised on 1700 children<br>Reliability: Internal consistency 0.91 to 0.93. Test-retest reliability 0.78 to 0.81<br>Validity: Correlation of language and cognition scales to WPPSI-III 0.79 to 0.82, correlation of motor scales with Peabody Developmental Motor Scales 0.49 to 0.57. Not intended to predict later function. Predictive validity has only been tested in preterm children and specificity was high, though sensitivity was low[3]
CAST†	1	Reliability: Test-retest reliability for continuous scale: spearman's rho 0.83. Using cut-off, Cohens kappa was 0.60[8]

		Validity: Sensitivity 100%, specificity 97%, positive predictive value 50%[9]
Differential ability scales, 2nd edition	1/2*	Standardised for 3480 children<br>Reliability: Test-rest reliability range from 0.51 to 0.92 with most items in the 0.70 to 0.80 range. Interrater reliability 0.95 to 0.99<br>Validity: Numbers not reported. Moderate to high correlation with WISC-IV and WPPSI-III. Medium to low correlation with BSID-III that measures different abilities[10]
Gesell development scales, revised	0/1*	Information on validity not found/not publicly available
Halstead Reitan Neuropsychological Battery	1	Information on validity not found/not publicly available for the scale used in preschool children
McCarthy's scales of children's abilities	3	No information on reliability<br>Validity: Correlation between McCarthy's General Cognitive Index and WPPSI was 0.63 to 0.72[11]
Movement ABC-2	1	Reliability: Test-retest reliability is 0.73 to 0.84<br>Validity: Content validity has been established by expert panel. The Movement ABC-2 has not been validated against a recognised measure of motor skill, such as Peabody Developmental Motor Scales[12]
NEPSY-II	1	Standardised for 1200 children<br>Reliability: Test-retest reliability range 0.50 to 0.86<br>No information on validity[13]
Reynell developmental language scale	2	Information on validity not found/not publicly available
Stanford Binet Intelligence Scale	2	Standardised for 1400 children<br>Reliability: Internal consistency 0.84 to 0.98. Test-retest reliability 0.76 to 0.95<br>Validity: Correlation to WPPSI-R 0.83[14]
Swedish language development scale‡	1	No information on reliability<br>Validity: Tested against Reynell developmental language scale assessed by a speech and language therapist. Sensitivity 0.69, specificity 0.93. PPV 0.52, NPV 0.96[15]
TEACh-5	1	Information on the original scale: Standardised for 293 children<br>Reliability: Test-retest reliability 0.64 to 0.92 with two of ten items below 0.70<br>Validity: Test demonstrates correlations to other measures of attention and little correlation with WISC-III that measures a different construct. Has ability to distinguish between children with and without ADHD[16]<br>Information on Teach-5, an adaption of the TEACh that is used for older children: Normed for 172 Danish children<br>Reliability: Moderate test-retest reliability, low for one of the items<br>Validity: The test maps on well to the original test and is little correlated to IQ[17]
Test of early language development, 3rd edition	1	Information on validity not found/not publicly available for the third edition of the test. The original version was standardised for 1184 children<br>Reliability: Internal consistency 0.87 to 0.92. Test-retest reliability 0.72 to 0.87<br>Validity: Correlation to Preschool language scale, test of language development and items from Metropolitan achievements tests and Metropolitan readiness tests 0.46 to 0.80[18]
Wisconsin Fine Motor Steadiness Battery	1	Information on validity not found/not publicly available
WPPSI-III	3/7*	Standardised for 1700 children<br>Reliability: Internal consistency 0.75 to 0.96, median 0.88. Test-retest reliability 0.86 to 0.92[14]<br>Validity: Correlation to BSID-II and DAS above 0.80

School child (6 to 12 years)		
NEPSY-II	1	Standardised for 1200 children<br>Reliability: Test-retest reliability range 0.50 to 0.86<br>No information on validity [13]
Reynell developmental language scale	1	Information on validity not found/not publicly available
SON-R	2	Only available in Dutch
WISC-III	1/2*	No information on reliability<br>Validity: Correlation to WPPSI-III above 0.80 [14]
WPPSI-III	2/4*	Standardised for 1700 children<br>Reliability: Internal consistency 0.75 to 0.96, median 0.88. Test-retest reliability 0.86 to 0.92<br>Validity: Correlation to BSID-II and DAS above 0.80[14]
Adolescent (13 to 18 years)		
WISC-III	1	Information on validity not found/not publicly available
**ii. Assessment by parents**		
Infants (<2 years)		
ASQ	2	Reliability: Internal consistency 0.49 to 0.87, lowest at youngest age groups. Test-retest reliability 0.94, interrater reliability 0.94.<br>Validity: Compared to revised Gesell and BSID for the youngest infants, Stanford Binet for 3 year olds and McCarthy scales for 4 year olds, the sensitivity was 0.75, specificity 0.86, PPV 0.46[19]
Brief infant sleep questionnaire	1	Reliability: Test-retest reliability 0.82 to 0.95<br>Validity: Correlation to sleep monitoring device (0.23 to 0.54) and daily sleep logs (0.27 to 0.83). Discriminative validity, clinical versus nonclinical group, correct assignment in 85%[20]
CBCL	5	Standardised for 700 preschool children (1 ½ -5 years)<br>Reliability: The internal consistency coefficients for the Syndrome scales and composites ranged from 0.66 to 0.95. Test-retest reliabilities ranged from 0.74 for the Attention-Deficit/Hyperactivity Problems DSM-Oriented scale to 0.92 for the Sleep Problems scale. The interrater agreement was 0.48 to 0.67[21]<br>Validity: Concurrent validity tested against DSM-IV diagnoses in a clinical sample. For anxiety and ADHD, validity was acceptable with area under the receiver operator curve (AUC) above 0.70. For depression, validity was acceptable for distinguishing between children with and without mood disorder, but the scale showed problems in distinguishing between children with depression and anxiety (AUC 0.65)[22]
Early infancy temperament questionnaire	1	Standardised for 404 children<br>Reliability: Internal consistency 0.42 to 0.76, test-retest 0.43 to 0.87[23]<br>Validity: Seven of nine dimensions were significantly correlated to comparable dimensions of the Infant behaviour questionnaire[24]
EAS	2	Information on validity of the original scale not found/not publicly available. Data is available for a Norwegian sample<br>Reliability: Internal consistency at 18 months 0.48 to 0.71. Test stability over time, 18 months to 3 years, 0.44 to 0.60, 18 months to 50 months 0.37 to 0.50<br>No information on validity[25]
Infant characteristic questionnaire	1	Reliability: Internal consistency 0.39 for the dimension Dull to 0.79 for Fussy-Difficult. Test-retest 0.47 for Unpredictable to 0.70 for Fussy-Difficult. Mother-father agreement 0.38 for Unpredictable to 0.61 for Fussy-Difficult<br>Validity: Moderate correlations to the Toddler temperament scale[26]
Motor milestones	1	No information on reliability<br>Parental rating of children's ability to walk briefly and sustained had concordance statistics 0.86 and 0.54 with the Alberta Infant

		Motor Scale[27]
Toddler temperament scale	2	Standardised on 309 children<br>Reliability: Internal consistency α=0.70 to 0.72. Test-retest reliability 0.81<br>Validity: Correlations of test scores to maternal perception of temperamental difficulty ranged from 0.09 to 0.58 across categories of the scale[28]
Preschool (2 to 5 years)		
ASQ	5	Reliability: Internal consistency 0.49 to 0.87, lowest at youngest age groups. Test-retest reliability 0.94, interrater reliability 0.94<br>Validity: Compared to revised Gesell and BSID for the youngest infants, Stanford Binet for 3 year olds and McCarthy scales for 4 year olds, the sensitivity was 0.75, specificity 0.86, PPV 0.46[19]
BRIEF	3	Standardised for 460 children<br>Reliability: Internal consistency 0.80 to 0.90. Test-retest reliability for most scales above 0.80, with one scale in the 0.70 to 0.79 range<br>Validity: Correlation to CPRS 0.81. Also high correlation to CBCL. 81% of children with autism, and 71% of children with ADHD, scored above cut-off on BRIEF[29]
CBCL	19	Standardised for 700 preschool children (1 ½ -5 years)<br>Reliability: The internal consistency coefficients for the Syndrome scales and composites ranged from 0.66 to 0.95. For the DSM-Oriented scales, internal consistencies ranged from 0.63 to 0.86. Test-retest reliabilities ranged from 0.74 for the Attention-Deficit/Hyperactivity Problems DSM-Oriented scale to 0.92 for the Sleep Problems scale. The interrater agreement was 0.48 to 0.67[21]<br>Validity: Concurrent validity tested against DSM-IV diagnoses in a clinical sample. For anxiety and ADHD, validity was acceptable with area under the receiver operator curve (AUC) above 0.70. For depression, validity was acceptable for distinguishing between children with and without mood disorder, but the scale showed problems in distinguishing between children with depression and anxiety (AUC 0.65)[22]
CPRS	3	Standardised for 1200 children<br>Reliability: Internal consistency coefficients for the content and *DSM* scales are all ≥0.80. Test-retest reliability ≥0.70). Interrater reliability ≥0.74 for the content and *DSM* scales<br>Validity: Moderate to high correlations with analogous scales from the BASC-2-PRS, CBCL, and BRIEF. Rates of correct ADHD classification based on content and *DSM* scale elevations were 57% to 86%[21]
EAS	4	Information on validity of the original scale not found/not publicly available. Data is available for a Norwegian sample<br>Reliability: Internal consistency at 3 years 0.54 to 0.73, and at 50 months 0.50 to 0.79. Test stability over time, 3 years to 50 months 0.46 to 0.61, 18 months to 50 months 0.37 to 0.50<br>No information on validity[25]
Intelligibility/Complexity of 3-year-old Children's Utterances	3	No information on reliability<br>Validity: Correlation to the McCarthy scales 0.48 to 0.68[30]
Motor milestones	1	No information on reliability<br>Parental rating of children's ability to walk briefly and sustained had concordance statistics 0.86 and 0.54 with the Alberta Infant Motor Scale[27]
SDQ	1	Reliability: Internal consistency 0.57 to 0.85. Test-retest after 4-6 months 0.57 to 0.72<br>Validity: Score above the cut-off and any DSM-IV diagnosis. Sensitivity 47%, specificity 94%, PPV 46%, NPV 96%[31]

School child (6 to 12 years)		
BRIEF	1	Standardised for 460 children<br>Reliability: Internal consistency 0.80 to 0.90. Test-retest reliability for most scales above 0.80, with one scale in the 0.70 to 0.79 range<br>Validity: Correlation to CPRS 0.81. Also high correlation to CBCL. 81% of children with autism, and 71% of children with ADHD, scored above cut-off on BRIEF[29]
CBCL	6	Standardised for 1753 school children (6-18 years)<br>Reliability: The internal consistency 0.78 to 0.97 on the Syndrome scales, 0.72 to 0.91 on the DSM-oriented scales, and 0.63 to 0.79 on the Competence scales. Test-retest reliabilities ranged from 0.80 for the Anxiety DSM-Oriented scale to 0.93 for the DSM-Oriented Conduct Problems scale. The interrater agreement was ≥ 0.63 for all scales except Activity[21]<br>Validity: Concurrent validity tested against DSM-IV diagnoses in a clinical sample. For anxiety and ADHD, validity was acceptable with area under the receiver operator curve (AUC) above 0.70. For depression, validity was acceptable for distinguishing between children with and without mood disorder, but the scale showed problems in distinguishing between children with depression and anxiety (AUC 0.65)[22]
CPRS	3	Standardised for 1200 children<br>Reliability: Internal consistency coefficients for the content and *DSM* scales are all ≥ 0.80. Test-retest reliability ≥ 0.70). Interrater reliability ≥ 0.74 for the content and *DSM* scales<br>Validity: Moderate to high correlations with analogous scales from CBCL, and BRIEF. Rates of correct ADHD classification based on content and *DSM* scale elevations were 57% to 86%[21]
Development and Well-Being Assessment (DAWBA)	1	Standardised for 491 community children and a clinical sample of 39 children<br>No information on reliability<br>Validity: The validation study did not distinguish between parent and teacher rating, so validity presented is for both. The assessment showed discrimination between clinical and community sample (11% in community versus 92% in clinical sample received diagnosis according to the scale). Correlated well with SDQ. In the clinical sample, agreement with case notes was substantial for 49%, partial for 46% and poor for 5%[32]
HBQ-P	2	Reliability: Test-retest, community sample 0.59 to 0.94, with most in the 0.70-0.79 range. Clinical sample, 0.51 to 0.88, with most in the 0.80-0.89 range. Mother-father agreement 0.26 to 0.52 (spearmans rho)<br>Validity: Showed discrimination between clinical and community sample and between different symptom groups, numbers not available[33]
SDQ	5	Reliability: Internal consistency 0.57 to 0.85. Test-retest after 4-6 months 0.57 to 0.72<br>Validity: Score above the cut-off and any DSM-IV diagnosis. Sensitivity 47%, specificity 94%, PPV 46%, NPV 96%[31]
Social responsiveness scale	1	No information on reliability<br>Validity: Sensitivity in parent report 0.78 to 1.00, specificity 0.67 to 1.00, PPV 0.33 to 63, NPV 0.81 to 1.00[34]
**iii. Assessment by teachers or others**		
Preschool (2 to 5 years)		
ADHD, DSM-IV form list	1	Diagnostic criteria for ADHD. It is unclear what the reliability and validity is, when the form is rated by teachers
BRIEF, teacher rated	1	Standardised for 302 children<br>Reliability: Internal consistency 0.90 to 0.97. Test-retest reliability for most scales above 0.80, with one scale in the 0.70 to 0.79

		range and one in the 0.60 to 0.69 range<br>Validity: Correlation to CPRS 0.81. Also high correlation to CBCL. 81% of children with autism, and 71% of children with ADHD, scored above cut-off on BRIEF[29]
CBCL, teacher rated	2	Standardised for 1192 children<br>Reliability: Internal consistency 0.52 to 0.96. Test-retest reliability 0.57 for Anxiety problems to 0.91 for Somatic complaints. Interrater reliability had a mean of 0.62, with a range of 0.21 (Somatic Complaints) to 0.78 (Aggressive Behavior)<br>Validity: Numbers not reported. The scale can differentiate clinical from nonclinical samples, with the exception of Somatic complaints scale. Teacher ratings at age 3 predict externalizing problems at age five[21]
California Preschool Social Competence Scale	1	Properties in a Spanish sample, as used in the study<br>Reliability: Internal consistency above 0.80 in 29 of 30 items. Test-retest reliability in median 0.78<br>Validity: Correlated with child IQ[35]
School child (6 to 12 years)		
CBCL, teacher rated	3	Standardised for 2319 children<br>Reliability: Internal consistency 0.72 to 0.95. Test-retest reliability ≥0.80, except for the Withdrawn/depressed scale (0.60) and Affective problems scale (0.62). Interrater reliability 0.49 for the Competence scales, 0.60 for the Syndrome scales, and .58 for the DSM-Oriented scales<br>Validity: Numbers not reported. The scale can differentiate clinical from nonclinical samples, with the exception of Somatic complaints scale[21]
Development and Well-Being Assessment (DAWBA), teacher rated	1	Standardised for 491 community children and a clinical sample of 39 children<br>No information on reliability<br>Validity: The validation study did not distinguish between parent and teacher rating, so validity presented is for both. The assessment showed discrimination between clinical and community sample (11% in community versus 92% in clinical sample received diagnosis according to the scale). Correlated well with SDQ. In the clinical sample, agreement with case notes was substantial for 49%, partial for 46% and poor for 5%[32]
SDQ, child rated	1	Reliability: Internal consistency 0.41 to 0.81. Test-retest after 4-6 months 0.21 to 0.62<br>Validity: Score above the cut-off and any DSM-IV diagnosis. Sensitivity 23%, specificity 94%, PPV 35%, NPV 92%[31]
**Assessment by medical diagnoses [data source in brackets]**[i]		
**F30-F39 (*Mood disorders*) [**Danish national registries]	1	Reliability: Inter-rater reliability of mood disorders in the ICD-10 system is 0.77 in an adult population[36]<br>Information on validity not found
Depression [Finnish national registries]	1	Reliability: Inter-rater reliability of depression in the ICD-10 system is 0.65 to 0.69 for severe depression, 0.31 to 0.42 for mild depression in an adult population[36]<br>Information on validity not found, but a review has looked at validity of diagnoses in the Finnish Hospital Discharge Register. For psychiatric disorders the sensitivity was between 0.57 and 0.75, and PPV was 0.75 to 1.00[37]
**F40-F48 (*Neurotic, stress-related and somatoform disorder*) [**Danish national registries]	1	Reliability: Inter-rater reliability of Neurotic, stress-related and somatoform disorder in the ICD-10 system is 0.74 in an adult population [36]<br>A diagnosis of F43 has been validated. In a sample of 200 persons with diagnosis and 100 without, the PPV was 0.58 to 0.83 in an adult population[38]
Anxiety [Finnish national registries]	1	Reliability: Inter-rater reliability of anxiety in the ICD-10 system is 0.55 in an adult population [36]

		Information on validity not found, but a review has looked at validity of diagnoses in the Finnish Hospital Discharge Register. For psychiatric disorders the sensitivity was between 0.57 and 0.75, and PPV was 0.75 to 1.00[37]
**F70-F79 (*Mental retardation*) [**Danish national registries]	1	Reliability: Inter-rater reliability of mental retardation in the ICD-10 system is 0.77 in an adult population[36] Information on validity not found
**F70-F79 (*Mental retardation*) [**Swedish national registries]	1	Reliability: Inter-rater reliability of mental retardation in the ICD-10 system is 0.77 in an adult population[36] Information on validity not found
**F80-F89 (*Disorders of psychological development*)**		Information not available for specific disorders, but for ICD-10 criteria of disorders of psychological development in general, the inter-rater reliability was 0.77 in US and Canada, and 0.49 for the rest of the world[39]
Speech and language disorders [Finnish registries]	1	Reliability: See F80-F89 Information on validity not found, but a review has looked at validity of diagnoses in the Finnish Hospital Discharge Register. For psychiatric disorders the sensitivity was between 0.57 and 0.75, and PPV was 0.75 to 1.00[37]
Speech and language disorders [Group Health Cooperative]	1	In this study, all children have their diagnoses confirmed by clinical assessment
Disorders of scholastic skills [Finnish registries]	1	Reliability: See F80-F89 Information on validity not found, but a review has looked at validity of diagnoses in the Finnish Hospital Discharge Register. For psychiatric disorders the sensitivity was between 0.57 and 0.75, and PPV was 0.75 to 1.00.[37]
Disorders of motor skills [Finnish registries]	1	Reliability: See F80-F89 Information on validity not found, but a review has looked at validity of diagnoses in the Finnish Hospital Discharge Register For psychiatric disorders the sensitivity was between 0.57 and 0.75, and PPV was 0.75 to 1.00[37]
Disorders of motor skills **[**Group Health Cooperative]	1	In this study, all children have their diagnoses confirmed by clinical assessment
Autism spectrum disorders [Canada, registries in Ontario]	1	Reliability: See F80-F89 Information on validity not found, but the authors refer to a study that assessed validity of US claims data from an administrative claims database of an unspecified national health insurance plan. Chart review for 432 children. If the chart should contain ≥2 claims with a diagnosis of autism spectrum disorder, PPV was 0.61 to 0.87[40]
Autism spectrum disorders [Canada, registries in Quebec]	1	Reliability: See F80-F89 Information on validity not found
Autism spectrum disorders [Childhood Autism Risks from Genetics and the Environment Study, California]	1	In this study, all children have their diagnoses confirmed by clinical assessment
Autism spectrum disorders [Danish national registries]	5	Reliability: See F80-F89 Diagnoses of F84.0 (childhood autism) have been validated for 499 children. Diagnosis of infantile autism was confirmed in 94%. If any ASD diagnosis was considered a confirmation of the F84.0 diagnosis in the record, 97% of recorded diagnoses were confirmed[41]
Autism spectrum disorders [Finnish national registries]	1	Reliability: See F80-F89 A validation study in 95 children found a PPV of 0.96[42]
Autism spectrum disorders [Meuhedet, Israel]	1	Reliability: See F80-F89 Information on validity not found
Autism spectrum disorders [Kaiser Permanente, California]	1	Reliability: See F80-F89 The authors refer to another publication from their group, where an unpublished validation study is described. 35 children with an

		ASD diagnosis were evaluated. 54% had ASD according to DSM-IV criteria and the remaining 46% had ASD according to clinical impression (not further defined)[43]
Autism spectrum disorder [Massachusetts registries]	2	Reliability: See F80-F89<br>Information on validity not found
Autism spectrum disorder [Study to Explore Early Development]	1	In this study, all children have their diagnoses confirmed by clinical assessment
Autism spectrum disorders [Swedish national registries]	3	Reliability: See F80-F89<br>In a chart review of 177 children, diagnosis was confirmed in 96%[44]
Other developmental delay [Childhood Autism Risks from Genetics and the Environment Study, California]	1	In this study, all children have their diagnoses confirmed by clinical assessment
Developmental delay/disorder [Study to Explore Early Development]	1	In this study, all children have their diagnoses confirmed by clinical assessment
**F90-F98 (*Behavioural and emotional disorders with onset usually occurring in childhood or adolescence*)** [Danish national registries]	1	Reliability: Inter-rater reliability of behavioural and emotional disorders in the ICD-10 system is 0.74[36]<br>A diagnosis of F91 (ADHD) has been validated in 372 patients. Diagnosis was confirmed for 87%[45]
ADHD [Canada, registries in Quebec]	1	Reliability: Information on reliability specific to the ADHD diagnosis not found<br>Information on validity not found
ADHD [Danish national registries]	2	Reliability: Information on reliability specific to the ADHD diagnosis not found<br>A diagnosis of F91 (ADHD) has been validated in 372 patients. Diagnosis was confirmed for 87%[45]
ADHD [Finnish national registries]	1	Reliability: Information on reliability specific to the ADHD diagnosis not found<br>Information on validity not found, but a review has looked at validity of diagnoses in the Finnish Hospital Discharge Register. For psychiatric disorders the sensitivity was between 0.57 and 0.75, and PPV was 0.75 to 1.00[37]
ADHD [Hong Kong territorial registries]	1	Reliability: Information on reliability specific to the ADHD diagnosis not found<br>Information on validity not found
ADHD [MarketScan claims data, unclear whether DSM or ICD-codes were used]	1	Reliability: Information on reliability specific to the ADHD diagnosis not found<br>Information on validity not found
ADHD [Massachusetts registries]	2	Reliability: Information on reliability specific to the ADHD diagnosis not found<br>Information on validity not found
ADHD [Norwegian national registries]	1	Reliability: Information on reliability specific to the ADHD diagnosis not found<br>Information on validity not found
ADHD [Maternal report in Nurses' Health Study II]	1	Information on reliability not found<br>Validity: Validated for 92 children, using ADHD Rating Scale-IV. If the criterion was set as a score above 90% on the ADHD Rating Scale, PPV was 1.00 for girls and 0.64 for boys. With a criterion of a score above 80%, the PPV for boys increased to 0.81[46]
ADHD [Swedish national registries]	1	Reliability: Information on reliability specific to the ADHD diagnosis not found<br>Information on validity not found

*Including other versions of the test.

†Structured interview of parents by health care professional

‡Mixture of parental questionnaires and nurse observation

[i]All studies where it is specified which diagnostic system is used, have used ICD-codes. Therefore we report reliability of ICD-codes rather than DSM-codes.
ADHD: Attention Deficit Hyperactivity Disorder, ASD: Autism Spectrum Disorder, ASQ: Ages and Stages Questionnaire, BNBAS: Brazelton Neonatal Behavoiral Assessment Scale, BRIEF: Behaviour Rating Inventory of Executive Function, BSID: Bayley Scales of Infant Development, CAST: Childhood Autism Spectrum Test, CBCL: Child Behaviour Checklist, CPRS: Conners' Parent Rating Scale, DAS: Differential ability scales, EAS: Emotionality, Activity, Sociability Temperament Survey, HBQ-P: MacArthur Health and Behaviour Questionnaire, NPV: Negative predictive value, PPV: Positive predictive values, SDQ: Strengths and Difficulties Questionnaire, SON-R: Snijders–Oomen Niet-verbale intelligentie Test–Revisie. TEACh-5: Test of everyday attention, 5 years, WISC: Wechsler Intelligence Scale for Children, WPPSI: Wechsler Preschool and Primary Scale of Intelligence.

References specific to this supplement:

1. Autti-Rämö, I. & Granström, M. L. The effect of intrauterine alcohol exposition in various durations on early cognitive development. *Neuropediatrics* **22**, 203–210 (1991).
2. Aylward, G. P. The Bayley Infant Neurodevelopmental Screener (BINS). in *Bayley-III Clinical Use and Interpretation* 201–233 (Elsevier, 2010). doi:10.1016/B978-0-12-374177-6.10007-8
3. Anderson, P. J. & Burnett, A. Assessing developmental delay in early childhood — concerns with the Bayley-III scales. *Clin. Neuropsychol.* **31**, 371–381 (2017).
4. Smith, J. A., Bidder, R. T., Gardner, S. M. & Gray, O. P. Griffiths Scales of Mental Development and different users. *Child Care Health Dev.* **6**, 11–16 (1980).
5. Heineman, K. R. & Hadders-Algra, M. Evaluation of neuromotor function in infancy-A systematic review of available methods. *J. Dev. Behav. Pediatr. JDBP* **29**, 315–323 (2008).
6. Einspieler, C. & Prechtl, H. F. R. Prechtl's assessment of general movements: A diagnostic tool for the functional assessment of the young nervous system. *Ment. Retard. Dev. Disabil. Res. Rev.* **11**, 61–67 (2005).
7. Hadders-Algra, M., Heineman, K. R., Bos, A. F. & Middelburg, K. J. The assessment of minor neurological dysfunction in infancy using the Touwen Infant Neurological Examination: strengths and limitations. *Dev. Med. Child Neurol.* **52**, 87–92 (2010).
8. Williams, J. *et al.* The Childhood Asperger Syndrome Test (CAST): Test–retest reliability. *Autism* **10**, 415–427 (2006).
9. Williams, J. *et al.* The CAST (Childhood Asperger Syndrome Test): Test accuracy. *Autism* **9**, 45–68 (2005).
10. Beran, T. N. Elliott, C. D. (2007). Differential Ability Scales (2nd ed.). San Antonio, TX: Harcourt Assessment. *Can. J. Sch. Psychol.* **22**, 128–132 (2007).
11. Arinoldo, C. G. Concurrent validity of McCarthy's Scales. *Percept. Mot. Skills* **54**, 1343–1346 (1982).

12. Brown, T. & Lalor, A. The Movement Assessment Battery for Children—Second Edition (MABC-2): A Review and Critique. *Phys. Occup. Ther. Pediatr.* **29**, 86–103 (2009).

13. Memory assessment. in *The Oxford handbook of child psychological assessment* (eds. Saklofske, D. H., Reynolds, C. R. & Schwean, V. L.) (Oxford University Press, 2013).

14. Lichtenberger, E. O. General measures of cognition for the preschool child. *Ment. Retard. Dev. Disabil. Res. Rev.* **11**, 197–208 (2005).

15. Mattsson, C. M., Mårild, S. & Pehrsson, N. G. Evaluation of a language-screening programme for 2.5-year-olds at Child Health Centres in Sweden. *Acta Paediatr. Oslo Nor. 1992* **90**, 339–344 (2001).

16. Manly, T. *et al.* The differential assessment of children's attention: the Test of Everyday Attention for Children (TEA-Ch), normative sample and ADHD performance. *J. Child Psychol. Psychiatry* **42**, 1065–1081 (2001).

17. Underbjerg, M. *et al.* Separable Sustained and Selective Attention Factors Are Apparent in 5-Year-Old Children. *PLoS ONE* **8**, e82843 (2013).

18. Bartlett, A., Slade, D. & Bellerose, P. C. Test Review: The Test of Early Language Development (TELD). *Read. Teach.* **40**, 546–548 (1987).

19. Squires, J., Bricker, D. & Potter, L. Revision of a parent-completed development screening tool: Ages and Stages Questionnaires. *J. Pediatr. Psychol.* **22**, 313–328 (1997).

20. Sadeh, A. A Brief Screening Questionnaire for Infant Sleep Problems: Validation and Findings for an Internet Sample. *PEDIATRICS* **113**, e570–e577 (2004).

21. Frick, P. J., Barry, C. T. & Kamphaus, R. W. Parent and Teacher Rating Scales. in *Clinical Assessment of Child and Adolescent Personality and Behavior* 141–188 (Springer US, 2009). doi:10.1007/978-1-4419-0641-0_7

22. Ebesutani, C. *et al.* Concurrent Validity of the Child Behavior Checklist DSM-Oriented Scales: Correspondence with DSM Diagnoses and Comparison to Syndrome Scales. *J. Psychopathol. Behav. Assess.* **32**, 373–384 (2010).

23. Medoff-Cooper, B., Carey, W. B. & McDevitt, S. C. The Early Infancy Temperament Questionnaire. *J. Dev. Behav. Pediatr. JDBP* **14**, 230–235 (1993).

24. Worobey, J. Convergence between temperament ratings in early infancy. *J. Dev. Behav. Pediatr. JDBP* **18**, 260–263 (1997).

25. Mathiesen, K. S. & Tambs, K. The EAS temperament questionnaire--factor structure, age trends, reliability, and stability in a Norwegian sample. *J. Child Psychol. Psychiatry* **40**, 431–439 (1999).
26. Bates, J. E., Freeland, C. A. & Lounsbury, M. L. Measurement of infant difficultness. *Child Dev.* **50**, 794–803 (1979).
27. Bodnarchuk, J. L. & Eaton, W. O. Can parent reports be trusted? *J. Appl. Dev. Psychol.* **25**, 481–490 (2004).
28. Fullard, W., McDevitt, S. C. & Carey, W. B. Assessing temperament in one- to three-year-old children. *J. Pediatr. Psychol.* **9**, 205–217 (1984).
29. Sherman, E. M. S. & Brooks, B. L. Behavior Rating Inventory of Executive Function – Preschool Version (BRIEF-P): Test Review and Clinical Guidelines for Use. *Child Neuropsychol.* **16**, 503–519 (2010).
30. Dale, P. S., Price, T. S., Bishop, D. V. M. & Plomin, R. Outcomes of early language delay: I. Predicting persistent and transient language difficulties at 3 and 4 years. *J. Speech Lang. Hear. Res. JSLHR* **46**, 544–560 (2003).
31. Goodman, R. Psychometric properties of the strengths and difficulties questionnaire. *J. Am. Acad. Child Adolesc. Psychiatry* **40**, 1337–1345 (2001).
32. Goodman, R., Ford, T., Richards, H., Gatward, R. & Meltzer, H. The Development and Well-Being Assessment: Description and Initial Validation of an Integrated Assessment of Child and Adolescent Psychopathology. *J. Child Psychol. Psychiatry* **41**, 645–655 (2000).
33. Essex, M. J. *et al.* The confluence of mental, physical, social, and academic difficulties in middle childhood. II: developing the Macarthur health and Behavior Questionnaire. *J. Am. Acad. Child Adolesc. Psychiatry* **41**, 588–603 (2002).
34. Hirota, T., So, R., Kim, Y. S., Leventhal, B. & Epstein, R. A. A systematic review of screening tools in non-young children and adults for autism spectrum disorder. *Res. Dev. Disabil.* **80**, 1–12 (2018).
35. Julvez, J. *et al.* Psychometric Characteristics of the California Preschool Social Competence Scale in a Spanish Population Sample. *Early Educ. Dev.* **19**, 795–815 (2008).
36. Sartorius, N. *et al.* Progress toward achieving a common language in psychiatry. Results from the field trial of the clinical guidelines accompanying the WHO classification of mental and behavioral disorders in ICD-10. *Arch. Gen. Psychiatry* **50**, 115–124 (1993).
37. Sund, R. Quality of the Finnish Hospital Discharge Register: A systematic review. *Scand. J. Public Health* **40**, 505–515 (2012).

38. Svensson, E., Lash, T. L., Resick, P. A., Hansen, J. G. & Gradus, J. L. Validity of reaction to severe stress and adjustment disorder diagnoses in the Danish Psychiatric Central Research Registry. *Clin. Epidemiol.* 235 (2015). doi:10.2147/CLEP.S80514
39. Regier, D. A., Kaelber, C. T., Roper, M. T., Rae, D. S. & Sartorius, N. The ICD-10 clinical field trial for mental and behavioral disorders: results in Canada and the United States. *Am. J. Psychiatry* **151**, 1340–1350 (1994).
40. Burke, J. P. *et al.* Does a claims diagnosis of autism mean a true case? *Autism Int. J. Res. Pract.* **18**, 321–330 (2014).
41. Lauritsen, M. B. *et al.* Validity of Childhood Autism in the Danish Psychiatric Central Register: Findings from a Cohort Sample Born 1990–1999. *J. Autism Dev. Disord.* **40**, 139–148 (2010).
42. Lampi, K. *et al.* Brief report: validity of Finnish registry-based diagnoses of autism with the ADI-R: Validity of registry-based diagnoses of autism. *Acta Paediatr.* **99**, 1425–1428 (2010).
43. Croen, L. A., Grether, J. K., Yoshida, C. K., Odouli, R. & Van de Water, J. Maternal autoimmune diseases, asthma and allergies, and childhood autism spectrum disorders: a case-control study. *Arch. Pediatr. Adolesc. Med.* **159**, 151–157 (2005).
44. Idring, S. *et al.* Autism Spectrum Disorders in the Stockholm Youth Cohort: Design, Prevalence and Validity. *PLoS ONE* **7**, e41280 (2012).
45. Mohr-Jensen, C., Vinkel Koch, S., Briciet Lauritsen, M. & Steinhausen, H.-C. The validity and reliability of the diagnosis of hyperkinetic disorders in the Danish Psychiatric Central Research Registry. *Eur. Psychiatry* **35**, 16–24 (2016).
46. Gao, X., Lyall, K., Palacios, N., Walters, A. S. & Ascherio, A. RLS in middle aged women and attention deficit/hyperactivity disorder in their offspring. *Sleep Med.* **12**, 89–91 (2011).
