# Supplementary material for: Use and validity of child neurodevelopment outcome measures in studies on prenatal exposure to psychotropic and analgesic medications – A systematic review
Source: PLoS One. 2019 Jul 11;14(7):e0219778. doi: 10.1371/journal.pone.0219778 (PMC6622545; doi:10.1371/journal.pone.0219778)
Supplement: S5 Table — (PDF) [file pone.0219778.s008.pdf]

S5 Table: Risk of bias assessment, papers on antidepressants.

		Risk of bias assessment by outcome	
Reference	Outcome	Strengths	Limitations
**Assessment using psychometric instruments**			
**i Assessment by health care professionals**			
Infant (<2 years)			
Suri 2011 [99]	BNBAS	Appropriate eligibility criteria<br>Blinded assessment<br>High rate of follow-up<br>No missing data	Conditioned on intermediates, but no confounders
Mortensen 2003 [81]	Boel test	Appropriate eligibility criteria<br>High rate of follow-up	Assessment not blinded<br>Conditioned on intermediates, but few confounders<br>Not mentioned how missing data were handled
Batton 2013 [44]	Bayley Infant Neurodevelopment Screener	Appropriate eligibility criteria<br>High rate of follow-up	Unclear whether assessment was blinded<br>Conditioned on intermediates, but no confounders<br>Not mentioned how missing data were handled
Weikum 2013a [101]	BSID, unspecified edition	Appropriate eligibility criteria<br>High rate of follow-up<br>No missing data	Unclear whether assessment was blinded<br>No conditioning on confounders
Gustafsson 2018 [61]	BSID-II	Appropriate eligibility criteria	Unclear whether assessment was blinded<br>Conditioned on intermediates, but few confounders<br>High rate of loss follow-up<br>Not mentioned how missing data were handled
Oberlander 2004 [87]	BSID-II	High rate of follow-up<br>No missing data	Exposed were recruited in pregnancy and post partum, unexposed were only recruited post partum<br>Unclear whether assessment was blinded<br>No conditioning on confounders
Reebye 2002 [92]	BSID-II	High rate of follow-up<br>No missing data	Exposed were recruited in pregnancy and post partum, unexposed were only recruited post partum<br>Unclear whether assessment was blinded<br>No conditioning on confounders

Reebye 2012 [38]	BSID-II	High rate of follow-up<br>No missing data	Exposed were recruited in pregnancy and post partum, unexposed were only recruited post partum<br>Unclear whether assessment was blinded<br>No conditioning on confounders
Santucci 2014 [93]	BSID-II	Appropriate eligibility criteria<br>Blinded assessment<br>No missing data	No conditioning on confounders<br>Differential loss to follow-up by exposure status
Austin 2013 [43]	BSID-III	Blinded assessment<br>High rate of follow-up<br>No missing data	Exposed and unexposed are recruited from different populations<br>No conditioning on confounders
Hanley 2013 [64]	BSID-III	Appropriate eligibility criteria<br>Blinded assessment<br>High rate of follow-up	Conditioned on some, but not all, important confounders<br>Unclear how missing data were handled
Heikkinen 2002 [67]	Gesell Development scales	High rate of follow-up<br>No missing data	Exposed were recruited in pregnancy, unexposed were recruited post partum<br>Unclear whether assessment was blinded<br>Conditioned on a few, but not all, important confounders
Heikkinen 2003 [36]	Gesell Development scales	High rate of follow-up<br>No missing data	Exposed were recruited in pregnancy, unexposed were recruited post partum<br>Unclear whether assessment was blinded<br>Conditioned on a few, but not all, important confounders
Johnson 2012 [73]	Infant Neurological International Battery	Blinded assessment<br>High rate of follow-up	Exposed were recruited in pregnancy, unexposed were recruited in pregnancy and post partum<br>Conditioned on some, but not all, important confounders<br>Unclear how missing data were handled
de Vries 2013 [53]	Psychomotor assessment according to Prechtl	Appropriate eligibility criteria<br>Blinded assessment<br>High rate of follow-up	Conditioned on intermediates, but few confounders<br>Not mentioned how missing data were handled
Preschool (2-5 years)			
Nulman 2002 [84]	BSID-II, Reynell developmental language scale,	Appropriate eligibility criteria<br>Blinded assessment<br>High rate of follow-up	Conditioned on many important confounders, but also on intermediates<br>Not mentioned how missing data were handled

	MSCA		
Nulman 1997 [83]	BSID-II	Appropriate eligibility criteria<br>Blinded assessment<br>High rate of follow-up	Conditioned on many important confounders, but also on intermediates<br>Not mentioned how missing data were handled
Casper 2003 [49]	BSID-II	Appropriate eligibility criteria<br>Blinded assessment<br>High rate of follow-up<br>No missing data	No conditioning on confounders
Batton 2013 [44]	BSID-III	Appropriate eligibility criteria<br>High rate of follow-up	Unclear whether assessment was blinded<br>Conditioned on intermediates, but no confounders<br>Not mentioned how missing data were handled
Galbally 2011 [57]	BSID-III	Blinded assessment<br>High rate of follow-up	Unclear how exposed and unexposed women are matched<br>No conditioning on confounding apart from unspecified matching<br>Not mentioned how missing data were handled
Hurault-Delarue 2016 [69]	Compulsory medical exam	Appropriate eligibility criteria<br>High rate of follow-up	Unclear whether assessment was blinded<br>Conditioned on intermediates, but few confounders<br>Not mentioned how missing data were handled
Schechter 2017 [94]	DAS	Appropriate eligibility criteria<br>Blinded assessment<br>Conditioned on many important confounders<br>High rate of follow-up	Not mentioned how missing data were handled
Johnson 2016 [74]	DAS-II, Test of early language development, 3rd edition (TELD-3)	Appropriate eligibility criteria<br>Blinded assessment<br>High rate of follow-up	Conditioned on some, but not all, important confounders<br>Not mentioned how missing data were handled
Galbally 2015 [58]	Movement ABC, WPPSI-III	Blinded assessment<br>High rate of follow-up	Unclear how exposed and unexposed women were matched<br>No conditioning on confounding apart from unspecified matching<br>Not mentioned how missing data were handled
Mattson	WPPSI-R	Appropriate eligibility criteria	Conditioned on a few, but not all, important confounders

2002 [79]		Blinded assessment<br>High rate of follow-up<br>No missing data	
School child (6-12 years)			
Nulman 1997 [83]	Reynell developmental language scale, MSCA	Appropriate eligibility criteria<br>Blinded assessment<br>High rate of follow-up	Conditioned on many important confounders, but also on intermediates<br>Not mentioned how missing data were handled
El Marroun 2017 [55]	SON-R (shortened), NEPSY-II	Appropriate eligibility criteria<br>Conditioned on many important confounders<br>High rate of follow-up<br>Missing data handled by multiple imputation	Unclear whether assessment was blinded
Hermansen 2016 [68]	NEPSY-II (shortened) WPPSI-R	Blinded assessment<br>No missing data	Unclear whether exposed and unexposed were recruited from the same population<br>No conditioning on confounders<br>High loss to follow-up
Nulman 2012 [85]	WPPSI-III	Appropriate eligibility criteria<br>Blinded assessment<br>High rate of follow-up	Conditioned on some, but not all, important confounders<br>Missing data handled through group mean imputation
Nulman 2015 [86]	WPPSI-III	Appropriate eligibility criteria<br>Blinded assessment<br>High rate of follow-up	Conditioned on many, not all, important confounders through sibling analysis, but conditioned on intermediates as well<br>Not mentioned how missing data were handled
Adolescent (13-18 years)			
Mattson 2002 [79]	WISC-III	Appropriate eligibility criteria<br>Blinded assessment<br>High rate of follow-up<br>No missing data	Conditioned on a few, but not all, important confounders
**ii Assessment by parents**			
Infant (<2 years)			
Brandlistuen	CBCL	Appropriate eligibility criteria	Assessment not blinded

2015 [46]		Conditioned on many important confounders and used sibling analysis<br>High rate of follow-up	Not mentioned how missing data were handled
Reebye 2002 [92]	Early infant temperament questionnaire	High rate of follow-up<br>No missing data	Exposed were recruited in pregnancy and post partum, unexposed were only recruited post partum<br>Assessment not blinded<br>No conditioning on confounders
Netsi 2015 [82]	Infant Characteristic Questionnaire, Brief Infant Sleep Questionnaire	Appropriate eligibility criteria<br>High rate of follow-up	Assessment not blinded<br>No conditioning on confounders<br>Not mentioned how missing data were handled
Nulman 1997 [83]	Toddler temperament scale	Appropriate eligibility criteria<br>High rate of follow-up	Conditioned on many important confounders, but also on intermediates<br>Assessment not blinded<br>Not mentioned how missing data were handled
Nulman 2002 [84]	Toddler temperament scale	Appropriate eligibility criteria<br>High rate of follow-up	Conditioned on many important confounders, but also on intermediates<br>Assessment not blinded<br>Not mentioned how missing data were handled
Preschool (2-5 years)			
Handal 2016a [62]	ASQ	Appropriate eligibility criteria<br>Conditioned on many important confounders and used negative controls	Assessment not blinded<br>High rate of loss follow-up<br>Not mentioned how missing data were handled
El Marroun 2017 [55]	BRIEF	Appropriate eligibility criteria<br>Conditioned on many important confounders<br>High rate of follow-up<br>Missing data handled by multiple imputation	Assessment not blinded
Galbally	CBCL, BRIEF	High rate of follow-up	Unclear how exposed and unexposed women were matched

2015 [58]			Assessment not blinded<br>No conditioning on confounding apart from unspecified matching<br>Not mentioned how missing data were handled
Brandlistuen 2015 [46]	CBCL	Appropriate eligibility criteria<br>Conditioned on many important confounders and used sibling analysis	Assessment not blinded<br>High rate of loss follow-up<br>Not mentioned how missing data were handled
Hanley 2015 [65]	CBCL	Appropriate eligibility criteria<br>Conditioned on many important confounders<br>High rate of follow-up	Assessment not blinded<br>Not mentioned how missing data were handled
Johnson 2016 [74]	CBCL	Appropriate eligibility criteria<br>High rate of follow-up	Conditioned on some, but not all, important confounders<br>Assessment not blinded<br>Not mentioned how missing data were handled
Misri 2006 [80]	CBCL	No missing data	Exposed were recruited in pregnancy, unexposed were recruited post partum<br>Assessment not blinded<br>No conditioning on confounding<br>High and differential rate of loss follow-up
Nulman 2002 [84]	CBCL	Appropriate eligibility criteria<br>High rate of follow-up	Conditioned on many important confounders, but also on intermediates<br>Assessment not blinded<br>Not mentioned how missing data were handled
Oberlander 2007 [88]	CBCL	No missing data	Exposed were recruited in pregnancy, unexposed were recruited post partum<br>Assessment not blinded<br>No conditioning on confounding<br>High and differential rate of loss follow-up
Oberlander 2010 [89]	CBCL	Appropriate eligibility criteria<br>High rate of follow-up	Assessment not blinded<br>Conditioned on few confounders and some intermediates<br>Not mentioned how missing data were handled

Lupattelli 2018 [76]	CBCL, EAS	Appropriate eligibility criteria<br>Conditioned on many important confounders and used negative controls<br>High rate of loss to follow-up, but the authors used inverse probability of censoring weights to handle this<br>Missing data handled by multiple imputation	Assessment not blinded
Handal 2016b [63]	Intelligibility/ Complexity of 3-year-old Children's Utterances	Appropriate eligibility criteria<br>Conditioned on many, but not all, important confounders	Assessment not blinded<br>High rate of loss follow-up<br>Not mentioned how missing data were handled
Skurveit 2014 [96]	Intelligibility/ Complexity of 3-year-old Children's Utterances	Appropriate eligibility criteria<br>Conditioned on many, but not all, important confounders	Assessment not blinded<br>High rate of loss follow-up<br>Not mentioned how missing data were handled
Pedersen 2013 [90]	SDQ	Appropriate eligibility criteria<br>Conditioned on many, but not all, important confounders<br>Differential loss to follow-up, but the authors examined the potential impact of this	Assessment not blinded<br>Not mentioned how missing data were handled
School child (6-12 years)			
Hutchison 2019 [70]	BRIEF	Appropriate eligibility criteria<br>High rate of follow-up	Conditioned on one confounder and one intermediate<br>Assessment not blinded<br>Not mentioned how missing data were handled
Hermansen 2016 [68]	CBCL	No missing data	Unclear whether exposed and unexposed were recruited from the same population<br>Assessment not blinded<br>No conditioning on confounders<br>High loss to follow-up

Nulman 1997 [83]	CBCL	Appropriate eligibility criteria<br>High rate of follow-up	Conditioned on many important confounders, but also on intermediates<br>Assessment not blinded<br>Not mentioned how missing data were handled
Nulman 2012 [85]	CBCL, CPRS	Appropriate eligibility criteria<br>High rate of follow-up	Conditioned on some, but not all, important confounders<br>Assessment not blinded<br>Missing data handled through group mean imputation
Nulman 2015 [86]	CBCL, CPRS-R	Appropriate eligibility criteria<br>High rate of follow-up	Conditioned on many, not all, important confounders through sibling analysis, but conditioned on intermediates as well<br>Assessment not blinded<br>Not mentioned how missing data were handled
El Marroun 2014 [54]	CBCL and Social responsiveness scale (SRS)	Appropriate eligibility criteria<br>Conditioned on many important confounders<br>High rate of follow-up<br>Missing data handled by multiple imputation	Assessment not blinded
Hanley 2015 [65]	HBQ-P	Appropriate eligibility criteria<br>Conditioned on many important confounders<br>High rate of follow-up	Assessment not blinded<br>Not mentioned how missing data were handled
Weikum 2013b [102]	HBQ-P	Appropriate eligibility criteria<br>High rate of follow-up	Conditioned on intermediates only<br>Assessment not blinded<br>Not mentioned how missing data were handled
Grzeskowiak 2016 [60]	SDQ	Appropriate eligibility criteria<br>Conditioned on many important confounders<br>High rate of loss to follow-up, but the authors used inverse probability of censoring weights to handle this	Assessment not blinded<br>Not mentioned how missing data were handled
**iii Assessment by teachers/others**			
Preschool (2-5 years)			

Johnson 2016 [74]	CBCL (other caregiver)	Appropriate eligibility criteria<br>High rate of follow-up	Conditioned on some, but not all, important confounders<br>Assessment not blinded<br>Not mentioned how missing data were handled
Misri 2006 [80]	CBCL (teacher)	No missing data	Exposed were recruited in pregnancy, unexposed were recruited post partum<br>Unclear whether assessment was blinded<br>No conditioning on confounding<br>High rate of loss follow-up
Oberlander 2007 [88]	CBCL (teacher)	No missing data	Exposed were recruited in pregnancy, unexposed were recruited post partum<br>Unclear whether assessment was blinded<br>No conditioning on confounding<br>High and differential rate of loss follow-up
**Assessment using medical diagnosis**			
Boukhris 2017 [34]	ADHD	Appropriate eligibility criteria<br>Conditioned on many important confounders<br>High rate of follow-up	Detection bias cannot be ruled out<br>Not mentioned how missing data were handled
Figueroa 2010 [56]	ADHD	Appropriate eligibility criteria<br>Conditioned on many important confounders<br>High rate of follow-up	Detection bias cannot be ruled out<br>Not mentioned how missing data were handled
Laugesen 2013 [37]	ADHD	Appropriate eligibility criteria<br>Conditioned on many important confounders and used sibling analysis<br>High rate of follow-up	Detection bias cannot be ruled out<br>Participants with missing data were excluded, but the extent of missing data is unclear
Man 2017 [78]	ADHD	Appropriate eligibility criteria<br>Conditioned on many important confounders and used negative controls and sibling analysis<br>High rate of follow-up	Detection bias cannot be ruled out<br>Not mentioned how missing data were handled

Castro 2016 [50]	ASD and ADHD	Appropriate eligibility criteria<br>Conditioned on many important confounders<br>High rate of follow-up	Detection bias cannot be ruled out<br>Not mentioned how missing data were handled
Clements 2015 [51]	ASD and ADHD	Appropriate eligibility criteria<br>Conditioned on many important confounders and used negative controls<br>High rate of follow-up	Detection bias cannot be ruled out<br>Not mentioned how missing data were handled
Sujan 2017 [98]	ASD and ADHD	Appropriate eligibility criteria<br>Conditioned on many important confounders and used sibling analysis<br>High rate of follow-up<br>Participants with missing data were excluded, but the extent of missing data was small	Detection bias cannot be ruled out
Wibroe 2017 [103]	ASD and ADHD	Appropriate eligibility criteria<br>High rate of follow-up<br>Participants with missing data were excluded, but the extent of missing data was very small	Detection bias cannot be ruled out<br>No conditioning on confounding
Malm 2016 [77]	ASD, depression, anxiety, ADHD	Appropriate eligibility criteria<br>Routine assessments of all children limit the risk of detection bias<br>Conditioned on many important confounders<br>High rate of follow-up<br>Authors have assessed that missing data was missing completely at random	
Liu 2017	ASD, F30-39, F40-	Appropriate eligibility criteria	Detection bias cannot be ruled out

[75]	49, F70-79, F90-99	Conditioned on many important confounders and used negative controls<br>High rate of follow-up<br>Missing data handled by multiple imputation	
Boukhris 2016 [45]	ASD	Appropriate eligibility criteria<br>Conditioned on many important confounders and used negative controls<br>High rate of follow-up	Detection bias cannot be ruled out<br>Not mentioned how missing data were handled
Brown 2017 [48]	ASD	Appropriate eligibility criteria<br>Conditioned on many important confounders and used sibling analysis<br>High rate of follow-up<br>Participants with missing data were excluded, but the extent of missing data was small	Detection bias cannot be ruled out
Croen 2011 [52]	ASD	Appropriate eligibility criteria<br>Conditioned on many important confounders and used negative controls<br>High rate of follow-up	Detection bias cannot be ruled out<br>Unclear how missing data were handled
Gidaya 2014 [59]	ASD	Appropriate eligibility criteria<br>High rate of follow-up	Detection bias cannot be ruled out<br>Conditioned on some, but not all, important confounders<br>Not mentioned how missing data were handled
Hviid 2013 [71]	ASD	Appropriate eligibility criteria<br>Conditioned on many important confounders and used negative controls<br>High rate of follow-up	Detection bias cannot be ruled out

		Used group mean imputation, but the extent of missing data was small, and authors assessed the implications of using this method	
Janecka 2018 [72]	ASD	Appropriate eligibility criteria<br>High rate of follow-up	Detection bias cannot be ruled out<br>Conditioned on some, but not all, important confounders<br>Not mentioned how missing data were handled
Rai 2017 [91]	ASD	Appropriate eligibility criteria<br>Conditioned on many important confounders, and used sibling analysis and negative controls<br>High rate of follow-up<br>Participants with missing data were excluded, but the extent of missing data was small	Detection bias cannot be ruled out
Sorensen 2013 [97]	ASD, infantile autism	Appropriate eligibility criteria<br>Conditioned on many important confounders and used negative controls and sibling analysis<br>High rate of follow-up<br>Participants with missing data were excluded, but the extent of missing data was small	Detection bias cannot be ruled out
Viktorin 2017b [41]	ASD	Appropriate eligibility criteria<br>High rate of follow-up<br>Participants with missing data were excluded, but the extent of missing data was small	Detection bias cannot be ruled out<br>Conditioned on some, but not all, important confounders
Harrington 2014 [66]	ASD, developmental delay (DD)	Appropriate eligibility criteria<br>Both cases and controls were assessed for ASD, thus limiting risk of detection bias	Conditioned on some, but not all, important confounders<br>Not mentioned how missing data were handled

		High rate of follow-up	
Brown 2016 [47]	Disorders of speech/ language, motor skills, and scholastic skills	Appropriate eligibility criteria<br>Routine assessments of all children limit the risk of detection bias<br>Conditioned on many important confounders<br>High rate of follow-up	Not mentioned how missing data were handled
Simon 2002 [95]	Developmental delay of motor skills, developmental delay of speech	Appropriate eligibility criteria<br>High rate of follow-up	Detection bias cannot be ruled out<br>Conditioned on some, but not all, important confounders<br>Not mentioned how missing data were handled
Viktorin 2017a [100]	Intellectual disability	Appropriate eligibility criteria<br>High rate of follow-up<br>Participants with missing data were excluded, but the extent of missing data was small	Detection bias cannot be ruled out<br>Conditioned on some, but not all, important confounders

Reference numbers in brackets refer to the reference list in the article.

ADHD: Attention Deficit Hyperactivity Disorder, ASD: Autism Spectrum Disorder, ASQ: Ages and Stages Questionnaire, BNBAS: Brazelton Neonatal Behavoiral Assessment Scale, BRIEF: Behaviour Rating Inventory of Executive Function, BSID: Bayley Scales of Infant Development, CBCL: Child Behaviour Checklist, CPRS: Conners Parent Rating Scale, DAS: Differential ability scales, HBQ-P: MacArthur Health and Behaviour Questionnaire, MSCA: McCarthy's scales of children's abilities, SDQ: Strengths and Difficulties Questionnaire, SON-R: Snijders–Oomen Niet-verbale intelligentie Test–Revisie, WISC: Wechsler Intelligence Scale for Children, WPPSI: Wechsler Preschool and Primary Scale of Intelligence.
