# Supplementary material for: Use and validity of child neurodevelopment outcome measures in studies on prenatal exposure to psychotropic and analgesic medications – A systematic review
Source: PLoS One. 2019 Jul 11;14(7):e0219778. doi: 10.1371/journal.pone.0219778 (PMC6622545; doi:10.1371/journal.pone.0219778)
Supplement: S6 Table — (PDF) [file pone.0219778.s009.pdf]

S6 Table: Risk of bias assessment, papers on psychotropics except antidepressants.

Reference	Exposure	Outcome measure	Risk of bias assessment by outcome<br>Strengths	Limitations
**Assessment using psychometric instruments**				
**i Assessment by health care professionals**				
Infant (<2 years)				
Platt 1989 [114]	Antipsychotics	BSID	Appropriate eligibility criteria<br>High rate of follow-up	Unclear whether assessment was blinded<br>Conditioned only on parity<br>Not mentioned how missing data were handled
Peng 2013 [113]	Antipsychotics	BSID-III	Appropriate eligibility criteria<br>Blinded assessment<br>High rate of follow-up	Conditioned on few, but not all, important confounders<br>Not mentioned how missing data on covariates were handled
Johnson 2012 [73]	Antipsychotics	Infant Neurological International Battery	Blinded assessment<br>High rate of follow-up	Exposed were recruited in pregnancy, unexposed were recruited in pregnancy and post partum<br>Conditioned on some, but not all, important confounders<br>Unclear how missing data were handled
Mortensen 2003 [81]	Antipsychotics, anxiolytics	Boel test	Appropriate eligibility criteria<br>High rate of follow-up	Assessment not blinded<br>Conditioned on intermediates, but few confounders<br>Not mentioned how missing data were handled
Oberlander 2004 [87]	Anxiolytics (clonazepam combined with SSRI)	BSID-II	High rate of follow-up<br>No missing data	Exposed were recruited in pregnancy and post partum, unexposed were only recruited post partum<br>Unclear whether assessment were blinded<br>No conditioning on confounders
Reebye 2002	Anxiolytics	BSID-II	High rate of follow-up	Exposed were recruited in pregnancy and

[92]	(clonazepam combined with SSRI)		No missing data	post partum, unexposed were only recruited post partum<br>Unclear whether assessment was blinded<br>No conditioning on confounders
Reebye 2012 [38]	Anxiolytics (clonazepam combined with SSRI)	BSID-II	High rate of follow-up<br>No missing data	Exposed were recruited in pregnancy and post partum, unexposed were only recruited post partum<br>Unclear whether assessment was blinded<br>No conditioning on confounders
Viggedal 1993 [40]	Anxiolytics	Griffiths' mental development scale I<br>Neuropsychological assessment	Appropriate eligibility criteria<br>Blinded assessment<br>High rate of follow-up<br>No missing data	No conditioning on confounders
Gidai 2008a [105]	Anxiolytics (alprazolam)	Hungarian development test<br>Behavioural style questionnaire	High rate of follow-up<br>No missing data	Unexposed were siblings of exposed, but the statistical analysis did not take this into account<br>Unclear whether assessment was blinded<br>No conditioning on confounders
Gidai 2008b [106]	Anxiolytics (medazepam)	Hungarian development test<br>Behavioural style questionnaire	High rate of follow-up<br>No missing data	Unexposed were siblings of exposed, but the statistical analysis did not take this into account<br>Unclear whether assessment was blinded<br>No conditioning on confounders
Gidai 2008c [107]	Anxiolytics (chlordiazepoxide)	Hungarian development test<br>Behavioural style questionnaire	High rate of follow-up<br>No missing data	Unexposed were siblings of exposed, but the statistical analysis did not take this into account<br>Unclear whether assessment was blinded<br>No conditioning on confounders
Timmermann 2008b [112]	Anxiolytics (meprobamate)	Hungarian development test<br>Behavioural style questionnaire	High rate of follow-up<br>No missing data	Unexposed were siblings of exposed, but the statistical analysis did not take this into account<br>Unclear whether assessment was blinded

				No conditioning on confounders
Laegreid 1992 [108]	Anxiolytics	Touwen Neurologic Assessment, Clinical neurologic assessment	Appropriate eligibility criteria<br>High rate of follow-up<br>No missing data	Assessment not blinded<br>No conditioning on confounders
Petik 2008a [115]	Hypnotics (glutethimide)	Hungarian development test<br>Behavioural style questionnaire	High rate of follow-up<br>No missing data	Unexposed were siblings of exposed, but the statistical analysis did not take this into account<br>Unclear whether assessment was blinded<br>No conditioning on confounders
Petik 2008b [116]	Hypnotics (Amobarbital)	Hungarian development test<br>Behavioural style questionnaire	High rate of follow-up for Hungarian development test<br>No missing data	Unexposed were siblings of exposed, but the statistical analysis did not take this into account<br>Unclear whether assessment was blinded<br>No conditioning on confounders<br>High rate of loss to follow-up for Behavioural style questionnaire
Timmermann 2008a [117]	Hypnotics (barbital, hexobarbital, butobarbital)	Hungarian development test<br>Behavioural style questionnaire	High rate of follow-up for Hungarian development test<br>No missing data	Unexposed were siblings of exposed, but the statistical analysis did not take this into account<br>Unclear whether assessment was blinded<br>No conditioning on confounders<br>High rate of loss to follow-up for Behavioural style questionnaire
Preschool (2-5 years)				
Hurault-Delarue 2016 [69]	Antipsychotics, anxiolytics and hypnotics	Compulsory medical exam	Appropriate eligibility criteria<br>High rate of follow-up	Unclear whether assessment was blinded<br>Conditioned on intermediates, but few confounders<br>Not mentioned how missing data were handled
Schechter 2017 [94]	Antipsychotics, anxiolytics, hypnotics	DAS	Appropriate eligibility criteria Blinded assessment<br>Conditioned on many important	Not mentioned how missing data were handled

			confounders<br>High rate of follow-up	
Hartz 1975 [35]	Anxiolytics (meprobamate, chlordiazepoxide)	Stanford Binet Intelligence Scale	Appropriate eligibility criteria<br>High rate of follow-up	Unclear whether assessment was blinded<br>Conditioned on few, but not all, important confounders<br>Not mentioned how missing data were handled
Mattson 2002 [79]	Anxiolytics	WPPSI-R	Appropriate eligibility criteria Blinded assessment<br>High rate of follow-up<br>No missing data	Conditioned on a few, but not all, important confounders
School child (6-12 years)				
Platt 1989 [114]	Antipsychotic	Paediatric neurologic assessment	Appropriate eligibility criteria<br>High rate of follow-up	Unclear whether assessment was blinded<br>Conditioned only on parity<br>Not mentioned how missing data were handled
Adolescent (13-18 years)				
Mattson 2002 [79]	Anxiolytics	WISC-III	Appropriate eligibility criteria Blinded assessment<br>High rate of follow-up<br>No missing data	Conditioned on a few, but not all, important confounders
**ii Assessment by parents**				
Infant (<2 years)				
Reebye 2002 [92]	Anxiolytics (clonazepam combined with SSRI)	Early Infancy Temperament Questionnaire	High rate of follow-up<br>No missing data	Exposed were recruited in pregnancy and post partum, unexposed were only recruited post partum<br>Assessment not blinded<br>No conditioning on confounders
Preschool (2-5 years)				
Lupattelli 2019 [109]	Anxiolytics, hypnotics	ASQ, CPRS-R	Appropriate eligibility criteria<br>Conditioned on many important confounders and used negative	Assessment not blinded

			controls<br>High rate of loss to follow-up, but the authors used inverse probability of censoring weights to handle this<br>Missing data handled by multiple imputation	
Brandlistuen 2017 [104]	Anxiolytics, hypnotics	CBCL (shortened)	Appropriate eligibility criteria<br>Conditioned on many important confounders and used sibling analysis	Assessment not blinded<br>High rate of loss follow-up<br>Not mentioned how missing data were handled
Misri 2006 [80]	Anxiolytics (clonazepam combined with SSRI)	CBCL	No missing data	Exposed were recruited in pregnancy, unexposed were recruited post partum<br>Assessment not blinded<br>No conditioning on confounding<br>High and differential rate of loss follow-up
Odsbu 2015 [110]	Anxiolytics	Intelligibility/Complexity of 3-year-old Children's Utterances	Appropriate eligibility criteria<br>Conditioned on many important confounders and used negative controls	Assessment not blinded<br>High rate of loss follow-up<br>Not mentioned how missing data were handled
School child (6-12 years)				
Radojčić 2017 [111]	Anxiolytics	CBCL	Appropriate eligibility criteria<br>Conditioned on many important confounders<br>Missing data handled by multiple imputation	Assessment not blinded<br>Rate of loss to follow-up unclear
**iii Assessment by teachers/others**				
Preschool (2-5 years)				
Misri 2006 [80]	Anxiolytics (clonazepam combined with SSRI)	CBCL	No missing data	Exposed were recruited in pregnancy, unexposed were recruited post partum<br>Unclear whether assessment was blinded<br>No conditioning on confounding<br>High rate of loss follow-up

School child (6-12 years)				
Radojčić 2017 [111]	Anxiolytics	CBCL	Appropriate eligibility criteria<br>Conditioned on many important confounders<br>Missing data handled by multiple imputation	Unclear whether assessment was blinded<br>Rate of loss to follow-up unclear
**Assessment using medical diagnosis**				
Figueroa 2010 [56]	Anxiolytics	ADHD	Appropriate eligibility criteria<br>Conditioned on many important confounders<br>High rate of follow-up	Detection bias cannot be ruled out<br>Not mentioned how missing data were handled
Janecka 2018 [72]	Lithium	ASD	Appropriate eligibility criteria<br>High rate of follow-up	Detection bias cannot be ruled out<br>Conditioned on some, but not all, important confounders<br>Not mentioned how missing data were handled

Reference numbers in brackets refer to the reference list in the article.

ADHD: Attention Deficit Hyperactivity Disorder, ASD: Autism Spectrum Disorder, ASQ: Ages and Stages Questionnaire, BSID: Bayley Scales of Infant Development, CBCL: Child Behaviour Checklist, CPRS:R: Conners' Parent Rating Scale, revised, DAS: Differential ability scales, NPV: Negative predictive value, PPV: Positive predictive value, WISC: Wechsler Intelligence Scale for Children, WPPSI: Wechsler Preschool and Primary Scale of Intelligence.
