# Supplementary material for: Use and validity of child neurodevelopment outcome measures in studies on prenatal exposure to psychotropic and analgesic medications – A systematic review
Source: PLoS One. 2019 Jul 11;14(7):e0219778. doi: 10.1371/journal.pone.0219778 (PMC6622545; doi:10.1371/journal.pone.0219778)
Supplement: S7 Table — (PDF) [file pone.0219778.s010.pdf]

S7 Table: Risk of bias assessments, papers on analgesics.

Reference	Exposure	Outcome measure	Risk of bias assessment by outcome<br>Strengths	Limitations
**Assessment using psychometric instruments**				
**i Assessment by health care professionals**				
Infants (<2 years)				
Salokorpi 1996 [136]	Indomethacin	Autti-Rämö neurodevelop-mental test battery	Appropriate eligibility criteria<br>No missing data	Unclear whether assessment was blinded<br>The risk of confounding should be low given that the study was randomised<br>However, the sample was small and no data was shown to verify that randomisa-tion was successful in creating two equal groups<br>High rate of loss to follow-up
Amin 2008 [134]	Indomethacin	BSID-II, mental develop-ment index	Appropriate eligibility criteria	Unclear whether assessment was blinded<br>Conditioned on intermediates, and few confounders<br>High rate of loss to follow-up<br>Not mentioned how missing data were handled
Al-Alaiyan 1996 [133]	Indomethacin	Gesell development scales, revised	Appropriate eligibility criteria<br>High rate of follow-up<br>No missing data	Unclear whether assessment was blinded<br>Conditioned on intermediates, but no con-founders
Avella-Garcia 2016 [118]	Paracetamol	BSID, unspecified ed	Appropriate eligibility criteria<br>Conditioned on many important con-founders<br>High rate of follow-up	Unclear whether assessment was blinded<br>Not mentioned how missing data were handled
Preschool (2-5 years)				
Barr 1990 [137]	ASA	Items from Gross Motor Scale (University of Ore-	Appropriate eligibility criteria<br>Blinded assessment	Not mentioned how missing data on co-variates were handled

		gon Medical School), Gesell – and Bayley Scales, Wisconsin Fine Motor Steadiness Battery, and Halstead Reitan Neuropsychological Battery	Conditioned on many important confounders<br>High rate of follow-up	
Klebanoff 1988 [138]	ASA	Stanford Binet Intelligence Scale	Appropriate eligibility criteria<br>Blinded assessment<br>Differential loss to follow-up, but the authors addressed this in statistical analyses	Conditioned on some, but not all, important confounders<br>Not mentioned how missing data on covariates were handled
Streissguth 1987 [39]	ASA	WPPSI	Appropriate eligibility criteria<br>Blinded assessment<br>Conditioned on many important confounders and used negative controls	Rate of loss to follow-up unclear<br>Not mentioned how missing data on covariates were handled
Avella-Garcia 2016 [118]	Paracetamol	McCarthy Scales of Children's Abilities<br>CAST*	Appropriate eligibility criteria<br>Conditioned on many important confounders<br>High rate of follow-up	Unclear whether assessment was blinded<br>Not mentioned how missing data were handled
Bornehag 2018 [119]	Paracetamol	Swedish language development scale†	Appropriate eligibility criteria	Unclear whether assessment was blinded<br>Conditioned on some, but not all, important confounders<br>High rate of loss to follow-up<br>Participants with missing data were excluded, but the extent of missing data is unclear
Liew 2016b [124]	Paracetamol	TEACh-5	Appropriate eligibility criteria<br>Blinded assessment<br>Conditioned on many important confounders<br>High rate of follow-up<br>Missing data handled by multiple	

			imputation	
Liew 2016c [125]	Paracetamol	WPPSI-R, shortened	Appropriate eligibility criteria<br>Blinded assessment (not mentioned in this paper, but in another paper from the same study)<br>Conditioned on many important confounders<br>High rate of follow-up<br>Missing data handled by multiple imputation	
Child (6-12 years)				
Markovic 2019 [135]	NSAIDs	SON-R	Appropriate eligibility criteria<br>Conditioned on many important confounders and used negative controls<br>High rate of follow-up<br>Missing data handled by multiple imputation	Unclear whether assessment was blinded
Laue 2019 [121]	Paracetamol	WISC-IV	Appropriate eligibility criteria	Unclear whether assessment was blinded<br>Conditioned on some, but not all, important confounders<br>High rate of loss to follow-up<br>Participants with missing data were excluded, extent of missing data more than 30%
**ii Assessment by parents**				
Infants (<2 years)				
Vlenterie 2016 [42]	Paracetamol	Motor milestone questionnaire, ASQ, CBCL, EAS	Appropriate eligibility criteria<br>Conditioned on many important confounders	Assessment not blinded<br>High rate of loss to follow-up<br>Not mentioned how missing data were handled
Wood 2016a [140]	Triptans	ASQ, CBCL, EAS	Appropriate eligibility criteria<br>Conditioned on many important con-	Assessment not blinded<br>Unclear how missing data affected the

			founders<br>High rate of loss to follow-up, but the authors used inverse probability of censoring weights to handle this	results
Preschool (2-5 years)				
Markovic 2019 [135]	NSAIDs	CBCL	Appropriate eligibility criteria<br>Conditioned on many important confounders and used negative controls<br>High rate of follow-up<br>Missing data handled by multiple imputation	Assessment not blinded
Brandlistuen 2013 [120]	NSAID and paracetamol	Motor milestone questionnaire, ASQ, CBCL, EAS	Appropriate eligibility criteria<br>Conditioned on many important confounders and used sibling analysis and negative controls	Assessment not blinded<br>High rate of loss to follow-up<br>Not mentioned how missing data on covariates were handled
Liew 2014 [122]	Paracetamol	SDQ	Appropriate eligibility criteria<br>Conditioned on many important confounders<br>Missing data handled by multiple imputation	Assessment not blinded<br>High rate of loss to follow-up
Liew 2016b [124]	Paracetamol	BRIEF	Appropriate eligibility criteria<br>Conditioned on many important confounders<br>High rate of follow-up<br>Missing data handled by multiple imputation	Assessment not blinded
Skovlund 2017 [128]	Paracetamol and opioids	Intelligibility/Complexity of 3-year-old Children's Utterances & ASQ	Appropriate eligibility criteria<br>Conditioned on many important confounders and used negative controls	Assessment not blinded<br>High rate of loss to follow-up<br>Participants with missing data were excluded, more than 10% had missing data
Wood 2016b [141]	Triptans	CBCL	Appropriate eligibility criteria<br>Conditioned on many important con-	Assessment not blinded<br>High rate of loss to follow-up

			founders and used negative controls	Participants with missing data were excluded, more than 10% had missing data
Wood 2016c [142]	Triptans	ASQ, EAS	Appropriate eligibility criteria<br>Conditioned on many important confounders	Assessment not blinded<br>High rate of loss to follow-up<br>Participants with missing data were excluded, more than 10% had missing data
Harris 2018 [139]	Triptans	CBCL, ASQ, EAS	Appropriate eligibility criteria<br>Conditioned on many important confounders<br>High rate of loss to follow-up, but the authors used inverse probability of censoring weights to handle this	Assessment not blinded<br>Participants with missing data were excluded, unclear how many in the migraine sample this had missing covariate data
Child (6-12 years)				
Stergiakouli 2016 [129]	Paracetamol	SDQ	Appropriate eligibility criteria<br>Conditioned on many important confounders and used negative controls	Assessment not blinded<br>High rate of loss to follow-up<br>Not mentioned how missing data were handled
Tovo-Rodrigues 2018 [131]	Paracetamol	SDQ	Appropriate eligibility criteria<br>Conditioned on many important confounders<br>High rate of follow-up	Assessment not blinded<br>Not mentioned how missing data were handled
Thompson 2014 [130]	Paracetamol and ASA	SDQ, CPRS:R-L (only for paracetamol)	Appropriate eligibility criteria<br>Conditioned on many important confounders and used negative controls	Assessment not blinded<br>High rate of loss to follow-up<br>Not mentioned how missing data were handled
Ruisch 2018 [127]	Paracetamol and ASA	Development and Well-Being Assessment	Appropriate eligibility criteria<br>Conditioned on many important confounders	Assessment not blinded<br>High rate of loss to follow-up<br>Complete case analysis was done, unclear how many were excluded due to missing data on covariates
**iii Assessment by teachers/others**				
Preschool (2-5 years)				

Avella-Garcia 2016 [118]	Paracetamol	California Preschool Social Competence Scale<br>ADHD, DSM-IV form list	Appropriate eligibility criteria<br>Conditioned on many important confounders<br>High rate of follow-up	Unclear whether assessment was blinded<br>Not mentioned how missing data was handled
Liew 2016b [124]	Paracetamol	BRIEF	Appropriate eligibility criteria<br>Conditioned on many important confounders<br>High rate of follow-up<br>Missing data handled by multiple imputation	Unclear whether assessment was blinded
Child (6-12 years)				
Markovic 2019 [135]	NSAIDs	CBCL	Appropriate eligibility criteria<br>Conditioned on many important confounders and used negative controls<br>Missing data handled by multiple imputation	Unclear whether assessment was blinded<br>High rate of loss to follow-up
Thompson 2014 [130]	Paracetamol and ASA	SDQ (children)	Appropriate eligibility criteria<br>Conditioned on many important confounders and used negative controls	Unclear whether assessment was blinded<br>High rate of loss to follow-up<br>Not mentioned how missing data were handled
Ruisch 2018 [127]	Paracetamol and ASA	Development and Well-Being Assessment	Appropriate eligibility criteria<br>Conditioned on many important confounders	Unclear whether assessment was blinded<br>High rate of loss to follow-up<br>Complete case analysis was done, unclear how many were excluded due to missing data on covariates
**Assessment by medical diagnosis**				
Rubenstein 2019 [143]	Opioids	Developmental delay and ASD	Appropriate eligibility criteria<br>Detection bias should be low, as all children were screened<br>Participants with missing data were excluded, but the extent of missing data was small	Conditioned on some, but not all, important confounders<br>Loss to follow-up unclear

Janecka 2018 [72]	Triptans and paracetamol	ASD	Appropriate eligibility criteria<br>High rate of follow-up	Detection bias cannot be ruled out<br>Conditioned on some, but not all, important confounders<br>Not mentioned how missing data were handled
Liew 2016a [123]	Paracetamol	Infantile autism and ASD	Appropriate eligibility criteria<br>Conditioned on many important confounders<br>High rate of follow-up<br>Missing data handled by multiple imputation	Detection bias cannot be ruled out
Liew 2014 [122]	Paracetamol	HKD	Appropriate eligibility criteria<br>Conditioned on many important confounders<br>High rate of follow-up<br>Missing data handled by multiple imputation	Detection bias cannot be ruled out
Liew 2019 [126]	Paracetamol	ADHD	Appropriate eligibility criteria<br>Conditioned on many important confounders and used negative controls<br>High rate of follow-up	Detection bias cannot be ruled out, and as diagnosis was ascertained by maternal report, reporting bias could also occur<br>Complete case analysis 25% of the sample was excluded due to missing data for the fully adjusted analysis
Ystrom 2017 [132]	Paracetamol	ADHD	Appropriate eligibility criteria<br>Conditioned on many important confounders and used negative controls<br>High rate of follow-up<br>Missing data handled by multiple imputation	Detection bias cannot be ruled out

Reference numbers in brackets refer to the reference list in the article.

*Structured interview of parents by the health care professional.

† Mixture of parental questionnaires and nurse observation.

ADHD: Attention Deficit Hyperactivity Disorder, ASA: Acetylsalicylic acid, ASD: Autism Spectrum Disorder, ASQ: Ages and Stages Questionnaire, BRIEF: Behaviour Rating Inventory of Executive Function, BSID: Bayley Scales of Infant Development, CAST: Childhood Autism Spectrum Test, CBCL: Child Behaviour Checklist, CPRS:R-L: Conners Parent Rating Scale, revised, long format, EAS: Emotionality, Activity, Sociability Temperament Survey, HKD: Hyperkinetic disorder, SDQ: Strengths and Difficulties Questionnaire, SON-R: Snijders–Oomen Niet-verbale intelligentie Test–Revisie, TEACh-5: Test of everyday attention, 5 years, WISC: Wechsler Intelligence Scale for Children, WPPSI: Wechsler Preschool and Primary Scale of Intelligence.
